# Metal-free visible-light-enabled vicinal trifluoromethyl dithiolation of unactivated alkenes

**DOI:** 10.3762/bjoc.17.49

**Published:** 2021-02-24

**Authors:** Xiaojuan Li, Qiang Zhang, Weigang Zhang, Jinzhu Ma, Yi Wang, Yi Pan

**Affiliations:** 1 State Key Laboratory of Coordination Chemistry, Jiangsu Key Laboratory of Advanced Organic Materials, and Collaborative Innovation Center of Advanced Microstructures, School of Chemistry and Chemical Engineering, Nanjing University, Nanjing 210023, China; 2 School of pharmacy, Wannan Medical College, Wuhu, 241002, China

**Keywords:** alkenes, difunctionalization, metal-free, photoredox, trifluoromethylthiolation

## Abstract

The difunctionalization of alkenes involving a trifluoromethylthio group (SCF_3_) for the conversion of versatile and readily available olefins into structurally more complex molecules has been successfully studied. However, the disproportionate dithiolation of alkenes is unknown. Herein, a transition-metal-free protocol is presented for the vicinal trifluoromethylthio–thiolation of unactivated alkenes via a radical process under mild conditions with a broad substrate scope and excellent tolerance.

## Introduction

The incorporation of fluorine atoms into drug molecules will significantly enhance the physical, chemical, and biological properties of the pharmaceuticals [1–6]. Modifying drug candidates by introducing fluorine-containing (such as -CF_3_, -CF_2_H, -C_2_F_5_, -SCF_3_, -OCF_3_) moieties has become a substantial strategy for medicinal research [2,7–8]. Among the fluorinated functionalities, the trifluoromethylthio group (SCF_3_) has strong electron-withdrawing properties and a higher lipophilicity (π_R_ = 1.44), compared with CF_3_ (π_R_ = 0.88) and SCH_3_ (π_R_ = 0.61), which could improve the pharmaceuticals’ ability to cross lipid membranes [9–10]. Along these lines, the introduction of the SCF_3_ group into small molecules has attracted great attention in organofluorine methodology [11–17].

**Scheme 1:**
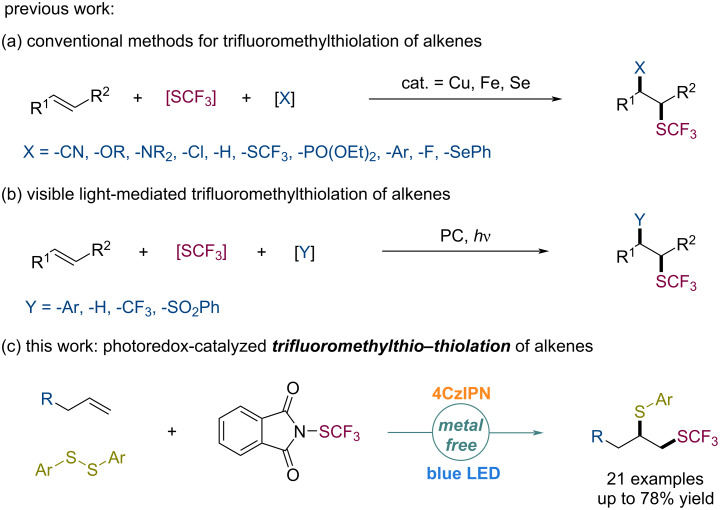
Origin of the reaction design.

The vicinal difunctionalization of olefins to introduce two functional groups across a double bond has appeared as a powerful transformation to rapidly increase molecular complexity in synthetic chemistry with improved efficiency [18–22]. Various transition-metal-mediated approaches for the trifluoromethylthio (SCF_3_) difunctionalization of alkenes, such as cyanation [23], etherification [24–27], amination [28–30], chlorination [31–32], hydrogenation [33], trifluoromethylation [34], phosphonization [35], arylation [36–38], trifluoromethylthiolation [39], fluorination [40], and selenylation [41] have been reported (Scheme 1a). However, the visible-light-induced trifluoromethylthio difunctionalization of alkenes remained underdeveloped. For instance, Magnier and co-workers have documented a practical intramolecular carbotrifluoromethylthiolation of acrylamides under irradiation of visible light [38]. In 2017, the photoredox-catalyzed intermolecular trifluoromethylthio–trifluoromethylation and thiosulfonylation reaction of unactivated alkenes have been respectively developed by Liu [34] and Xu [42]. Recently, Qing [43] and co-workers reported an efficient *anti*-Markovnikov hydrotrifluoromethylthiolation of alkenes utilizing trifluoromethanesulfonic anhydride (Tf_2_O) as a radical trifluoromethylthiolating reagent through a deoxygenative reduction and a photoredox radical pathway (Scheme 1b). The C–S bond [44–45] is an important structural motif that is widely present in natural products, drug molecules, biologically active molecules, and functional materials. However, the highly selective incorporation of two different sulfur-bearing moieties across double bonds remains challenging [42]. Herein, we describe a visible-light-enabled cascade radical difunctionalization of unactivated alkenes for the construction of partially trifluoromethylated dithioethers with a broad substrate scope and a good chemical tolerance (Scheme 1c).

## Results and Discussion

We evaluated the reaction conditions for this trifluoromethylthio–thiolation and found out that under irradiation of blue LEDs, allyl boronate **1a** (0.1 mmol), disulfide **2a** (1.0 equiv) and *N*-(trifluoromethylthio)phthalimide (Phth-SCF_3_, **3**, 1.5 equiv), the desired trifluoromethylthiolated product **4a** was obtained in 71% yield with 1,2,3,5-tetrakis(carbazol-9-yl)-4,6-dicyanobenzene (4CzIPN, 2 mol %) as the photocatalyst and KH_2_PO_4_ (10 mol %) as the base (Table 1, entry 1). The yield of **4a** was not increased when 2 equiv of K_2_HPO_4_ were used (Table 1, entry 2) and no difunctionalized product was observed with DMA as the solvent (Table 1, entry 4). The employment of KH_2_PO_4_ as base and [Ir(dF(CF_3_)ppy)_2_(dtbby)]PF_6_ as the photocatalyst furnished the product in very low yields (Table 1, entries 3 and 5). The control experiments indicated that 4CzIPN, K_2_HPO_4_ or visible-light were indispensable for the reaction to proceed (Table 1, entries 6–8).

**Table 1:** Optimization of the reaction conditions.^a^

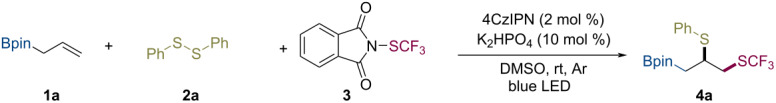		

entry	deviation from the reaction conditions	yield^b^ (%)

1	none	71
2	2.0 equiv K_2_HPO_4_	70
3	KH_2_PO_4_ instead of K_2_HPO_4_	34
4	DMA instead of DMSO	trace
5	[Ir(dF(CF_3_)ppy)_2_(dtbby)]PF_6_ instead of 4CzIPN	43
6	no K_2_HPO_4_	0
7	without 4CzIPN	0
8	in darkness	0

^a^Reaction conditions: **1a** (0.1 mmol), **2a** (1.0 equiv), **3** (1.5 equiv), 4CzIPN (2 mol %), K_2_HPO_4_ (10 mol %), rt, Ar, 24 h. ^b^Crude yields were determined by ^19^F NMR using trifluoromethoxybenzene as an internal standard.

**Scheme 2:**
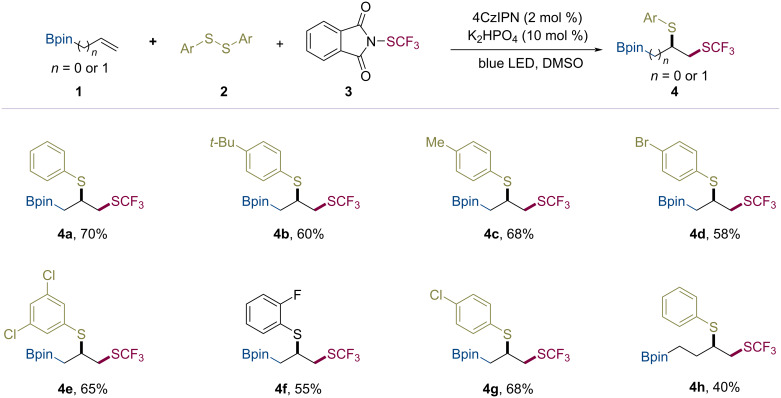
Substrate scope of disulfides.

With the optimized reaction conditions determined, we next examined the substrate scope of the disulfides (Scheme 2). Using borate-substituted olefins, the intermolecular trifluoromethylthio–thiolation induced by the sequential radical difunctionalization proceeded smoothly in a chemoselective fashion. Both, with electron-withdrawing and electron-donating groups substituted aryldisulfides were tolerated to access products **4a**–**g**. The homoallylic borate (**1b**) was also converted into the corresponding product **4h** in a moderate yield.

**Scheme 3:**
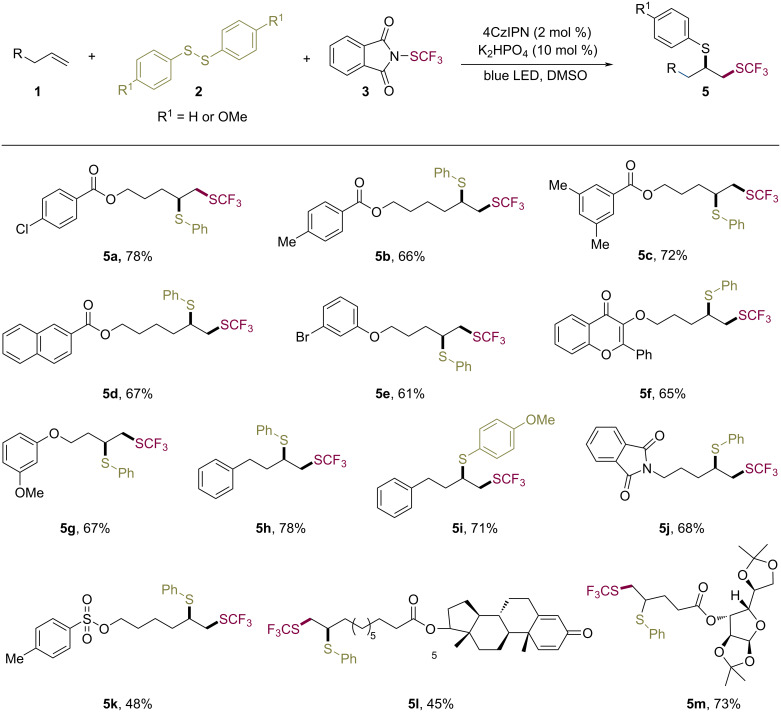
Substrate scope of unactivated alkenes.

In order to further examine the generality of the reaction, we have extended this protocol to a range of unactivated alkenes. Terminal alkenes containing ester (**5a**–**d**) and oxygenated alkyl (**5e**–**g**) functionalities were tolerated under this framework (Scheme 3). The tested 1-phenyl-3-butene substrates transformed into the desired products **5h** and **5i** in good yields. Also olefins containing amido (**5j**) and sulfonate (**5k**) functionalities furnished the corresponding SCF_3_ adducts. In addition, boldenone- and ᴅ-glucose-derived terminal alkenes were compatible with the conditions affording the corresponding products **5l** and **5m** in moderate to good yields.

**Scheme 4:**
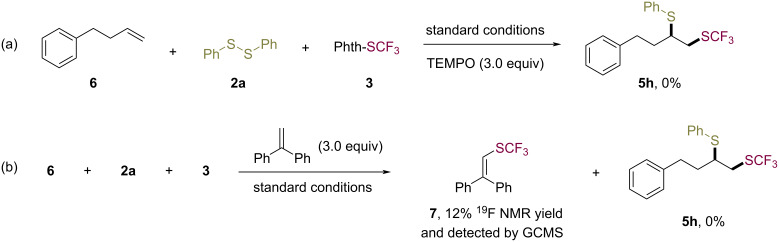
Control experiments.

To gain insight into the reaction mechanism, control experiments were conducted under the standard conditions (Scheme 4). The radical-trapping agent 2,2,6,6-tetramethylpiperidin-1-oxyl (TEMPO, 3.0 equiv) completely prevented the reaction. When diphenylethylene (3.0 equiv) was added to the reaction mixture, the desired product **5h** was not obtained, and the vinyltrifluoromethylsulfide **7** was afforded in 12% yield [46–47].

**Scheme 5:**
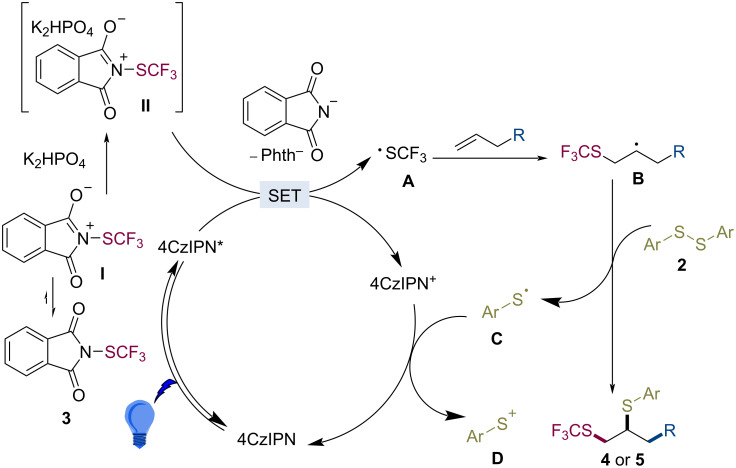
Proposed mechanism.

Based on the above results and previous literature reports [33,38,48], a plausible mechanism for the trifluoromethylthio–thiolation is proposed (Scheme 5). In solution, two resonance structures can be formulated for Phth-SCF_3_, **3** (*E*_1/2_^red^ = −0.45 V vs SCE) [49] and the intermediate **I** [50]. The complexation of the intermediate **I** with K_2_HPO_4_ provides the intermediate **II**. Then, 4CzIPN* (4CzIPN^+^/4CzIPN*: *E*_1/2_^red^ = −1.04 V vs SCE) [51] reduces the intermediate **II** to generate a phthalimide anion (Phth^−^ [50]) and a trifluoromethylthio radical under irradiation. The SCF_3_ radical readily reacts with the unactivated alkenes to give the intermediate **B**. The subsequent addition of **B** to disulfide **2** affords the difunctionalized products **4** or **5** and the thiophenyl radical **C** [52]. Finally, the oxidation of radical **C** by 4CzIPN^+^ closes the catalytic cycle [53].

## Conclusion

In summary, we described a visible-light-induced cascade radical difunctionalization of unactivated alkenes to provide partially trifluoromethylated dithioethers. The approach features practical conditions, good functional group tolerance, and a broad substrate scope allowing the incorporation of two distinctive sulfur-containing motifs into terminal olefins.

## Supporting Information

File 1 Full experimental details, compound characterization, and copies of NMR spectra.
