# Supplementary material for: Metal-free visible-light-enabled vicinal trifluoromethyl dithiolation of unactivated alkenes
Source: Beilstein J Org Chem. 2021 Feb 24;17:551–7. doi: 10.3762/bjoc.17.49 (PMC7934782; doi:10.3762/bjoc.17.49)
Supplement: File 1 — Full experimental details, compound characterization, and copies of NMR spectra. [file Beilstein_J_Org_Chem-17-551-s001.pdf]

BEILSTEIN JOURNAL OF ORGANIC CHEMISTRY

Supporting Information

for

Metal-free visible-light-enabled vicinal trifluoromethyl dithiolation of unactivated alkenes

Xiaojuan Li, Qiang Zhang, Weigang Zhang, Jinzhu Ma, Yi Wang and Yi Pan

Beilstein J. Org. Chem. **2021,** *17,* 551–557. doi:10.3762/bjoc.17.49

Full experimental details, compound characterization, and copies of NMR spectra

License and Terms: This is a supporting information file under the terms of the Creative Commons Attribution License (https://creativecommons.org/licenses/by/4.0). Please note that the reuse, redistribution and reproduction in particular requires that the author(s) and source are credited and that individual graphics may be subject to special legal provisions.
The license is subject to the *Beilstein Journal of Organic Chemistry* terms and conditions: (https://www.beilstein-journals.org/bjoc/terms)

Table of contents

I. General methods S1

II. Synthesis of starting materials S2

III. Optimization of the reaction conditions S3

IV. Experimental procedures S6

V. Mechanistic investigations S6

VI. Characterization data S8

VII. Spectral data S15

References S48

I. General methods

All reactions were performed in flame-dried glassware with equipped with a magnetic stirring bar and sealed with a rubber septum. The solvents were distilled by standard methods. Reagents were obtained from commercial suppliers and were used without further purification unless otherwise noted. Silica gel column chromatography was carried out using silica gel 60 (230–400 mesh). Analytical thin layer chromatography (TLC) was done using silica gel (silica gel 60 F254). TLC plates were analyzed by exposure to ultraviolet (UV) light. NMR experiments were performed using a Bruker AVANCE III-400 or 500 spectrometer and carried out in deuterochloroform ($CDCl_3$) ^{1}H NMR and ^{13}C NMR spectra were recorded at 400 MHz or 500 MHz and 100 MHz or 125 MHz, respectively. ^{19}F NMR spectra were recorded at 376 MHz or 470 MHz. Chemical shifts are reported as δ values relative to internal TMS (δ 0.00 for ^{1}H NMR), chloroform (δ 7.26 for ^{1}H NMR), chloroform (δ 77.00 for ^{13}C NMR). The following abbreviations are used for the multiplicities: s: singlet, d: doublet, dd: doublet of doublet, t: triplet, q: quadruplet, m: multiplet, br: broad signal for proton spectra; coupling constants (*J*) are reported in hertz (Hz). Melting points were uncorrected. Infrared spectra were obtained on an agilent Cary630. HRMS were recorded on a Bruker microTOF-Q111. GC–MS spectra were performed on a Shimadzu QP2010 (EI source). In a general experiment, 10 W blue LEDs (465 nm) were used as a blue light source. A borosilicate glass tube was used as a reaction tube. We have not used any filters. Unless otherwise noted, all reagents were weighed and handled in air, and all reactions were carried out under argon. Medium-sized screw-cap test tubes (8 mL) were used for all 0.20 mmol scale reactions: Fisher 13 × 100 mm tubes (Cat. No.1495935C). Cap with septum: Thermo Scientific ASM PHN CAP w/PTFE/SIL (Cat. No.03378316)

II. Synthesis of starting materials

Substrates **1a**, **1b**, **1c**, **1d**, **1e**, **1f**, **1g**, **1j**, **1k**, and **1m** were prepared according to the literature [1-5]. Substrates **1h** and **1l**, were purchased from commercial sources (Alfa, TCI, Energy and Macklin) and used as received

Procedure for the synthesis of *N*-(trifluoromethylthio)phthalimide (3)[6]

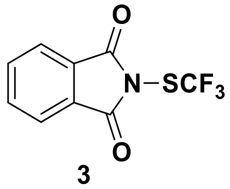

3

Under argon, dried CH_3CN (40 mL) was added to a mixture of *N*-bromophthalimide (5.2 g, 23 mmol), $AgSCF_3$ (6.25 g, 30 mmol) in a 100 mL round-bottomed flask. The mixture was stirred at room temperature for 3 h and then the solvent was evacuated under rotary evaporator. Before filtering the residue through a short plug of Celite, CH_2Cl_2 (20 mL) was added. Then, the filter residue was evacuated again under reduced pressure to obtain the target compound as a white solid (5.3 g, 93%). ***N*-(trifluoromethylthio)phthalimide (3)** ^{1}H NMR (500 MHz, chloroform-*d*) δ 8.02 (dd, *J* = 5.5, 3.1 Hz, 2H), 7.87 (dd, *J* = 5.5, 3.1 Hz, 2H). ^{19}F NMR (471 MHz, chloroform-*d*) δ −48.89.

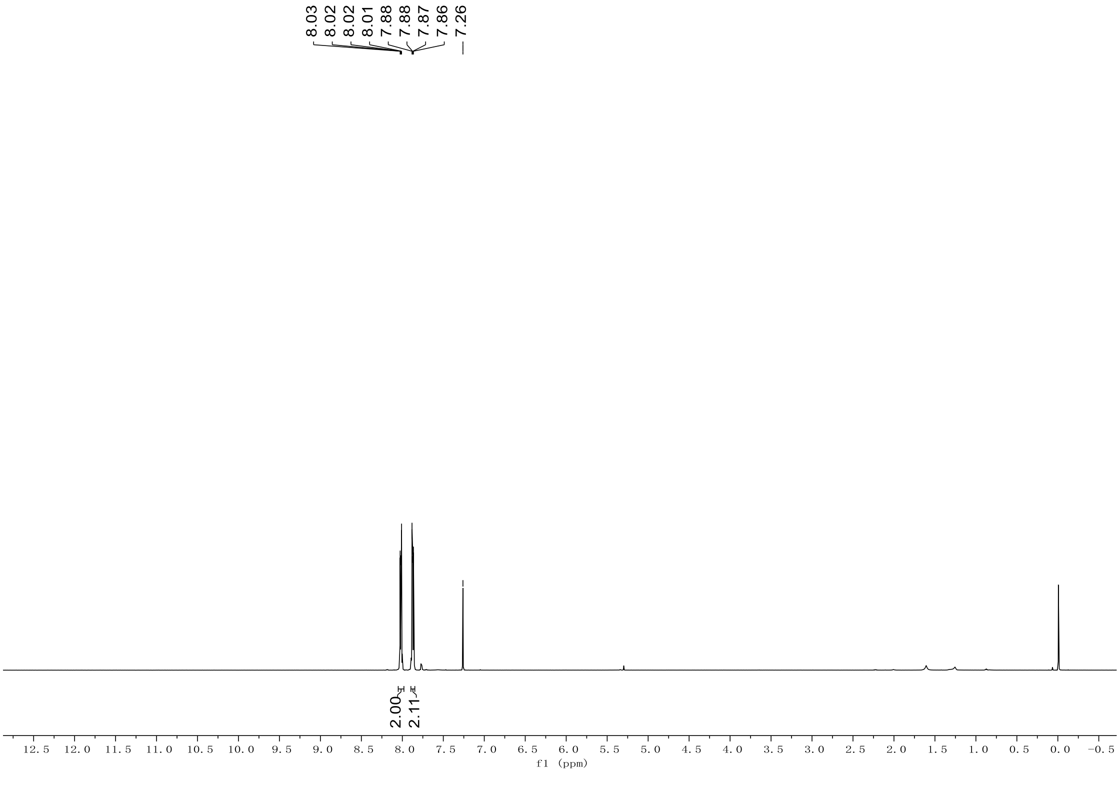

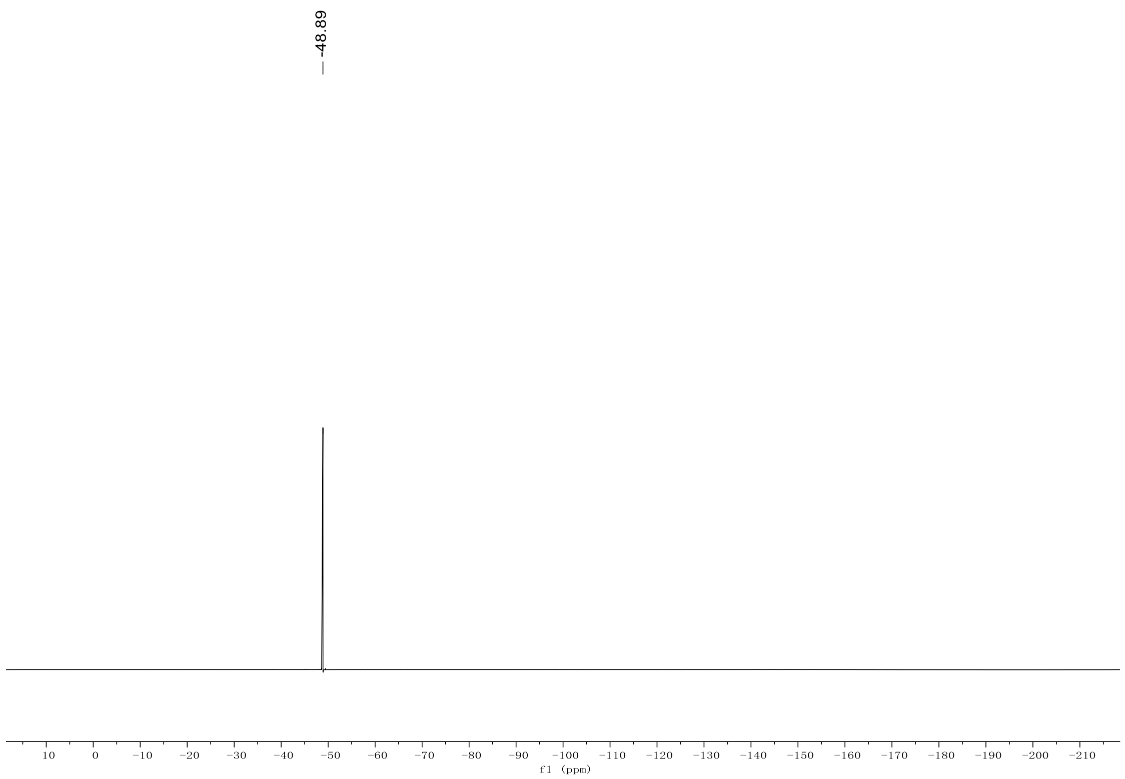

III. Optimization of the reaction conditions

Table S1. Optimization of solvent [a]

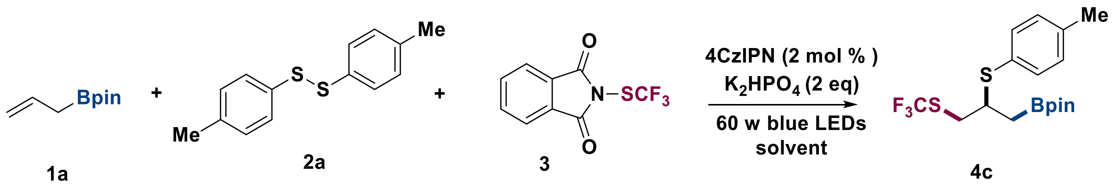

Entry	Solvent	Yield of **4c** [b](%)
1	MeCN	16
2	Acetone	n.r.
3	DMA	n.r.
4	EA	3
5	DCM	1
6	THF	n.r.
7	DMF	8
8	DMSO	20

[a] All reactions were carried out with **1a** (0.1 mmol, 1 equiv), **2a** (0.1 mmol, 1 equiv), **3** (1.2 equiv), K_2HPO_4 (2 equiv), photocatalyst 4CzIPN (2 mol %) in solvent (1.0 mL) at rt under Ar and 60 W blue LEDs.

[b] Crude yields were determined by ^{19}F NMR using trifluoromethoxybenzene as internal standard.

Table S2. Optimization of base [a]

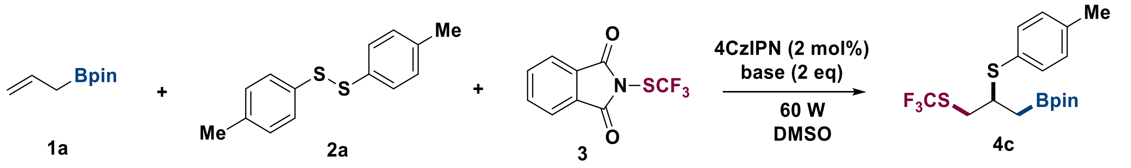

Entry	Base	Yield of **4c** [b](%)
1	LiOAc	n.r.
2	K_2HPO_4	25
3	KOOCH	n.r.
4	KF	n.r.
5	K_3PO_4	21
6	K_2CO_3	trace
7	Et_3CN	n.r.
8	NaH_2PO_4	20

[a] All reactions were carried out with **1a** (0.1 mmol, 1 equiv), **2a** (0.1 mmol, 1 equiv), **3** (1.2 equiv), photocatalyst 4CzIPN (2 mol %) in DMSO (1.0 mL) at rt under Ar and 60 W blue LEDs.

[b] Crude yields were determined by ^{19}F NMR using trifluoromethoxybenzene as internal standard.

Table S3. Optimization of the light source [a]

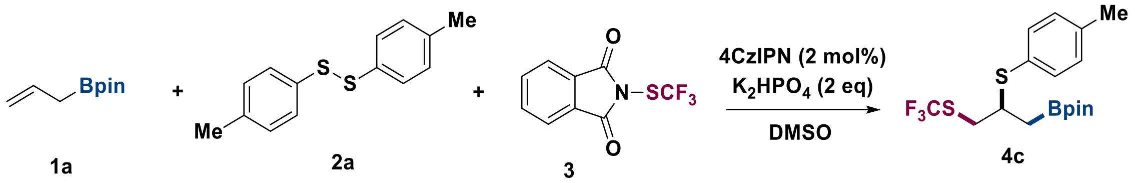

Entry	**Light source**	Yield of **4c** [b](%)
1	CFL	n.r.
2	10 W	32
3	30 W	17
4	60 W	21
5	90 W	14

[a] All reactions were carried out with **1a** (0.1 mmol, 1 equiv), **2a** (0.1 mmol, 1 equiv), **3** (1.2 equiv), K_2HPO_4 (2 equiv), photocatalyst 4CzIPN (2 mol %) in DMSO (1.0 mL) at rt under Ar and LEDs.

[b] Crude yields were determined by ^{19}F NMR using trifluoromethoxybenzene as internal standard.

Table S4. Optimization the amount of base [a]

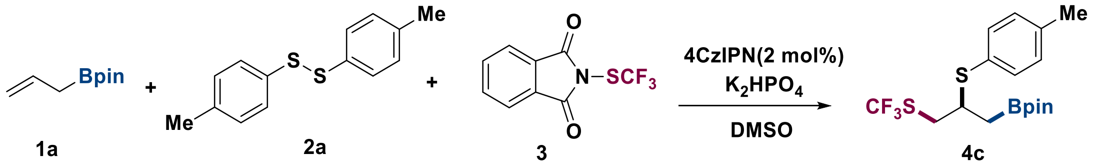

Entry	**K_2HPO_4 (eq)**	Yield of **4c** [b](%)
1	0	0
2	0.1	53
3	0.2	52
4	1	39
5	2	32
6	3	28

[a] All reactions were carried out with **1a** (0.1 mmol, 1 equiv), **2a** (0.1 mmol, 1 equiv), **3** (1.2 equiv), K_2HPO_4, photocatalyst 4CzIPN (2 mol %) in DMSO (1.0 mL) at rt under Ar and 10 W blue LEDs.
[b] Crude yields were determined by ^{19}F NMR using trifluoromethoxybenzene as internal standard.

Table S5. Optimization of the amount of 2a and 3 [a]

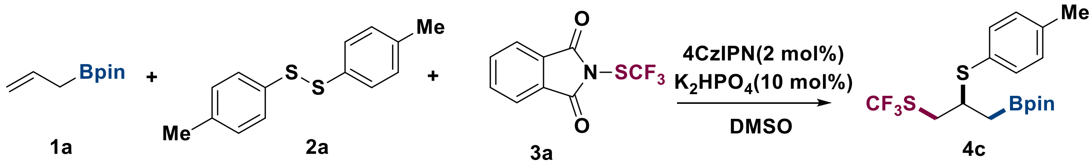

Entry	**3 (eq)**	**2a**	Yield of **4c** [b](%)
1	1.5	1	68
2	1.5	0.8	60
3	1.2	0.8	65
4	2	1	60
5	1	1	34

[a] All reactions were carried out with **1a** (0.1 mmol, 1 equiv), **2a**, **3**, K_2HPO_4 (10 mol %), photocatalyst 4CzIPN (2 mol %) in DMSO (1.0 mL) at rt under Ar and 10 W blue LEDs.
[b] Crude yields were determined by ^{19}F NMR using trifluoromethoxybenzene as internal standard.

IV. Experimental procedures

General procedure

Under argon, 4CzIPN (2 mol %) and *N*-(trifluoromethylthio)phthalimide (**3**, 1.5 equiv), **1a** (0.2 mmol, 1 equiv), **2a** (0.2 mmol, 1 equiv), and K_2HPO_4 (10 mol %) were dissolved in dried DMSO (2 mL) at room temperature. After that, the tube was exposed to 10 W blue LEDs and the mixture was stirred at room temperature for about 24 h until the reaction was completed as monitored by TLC analysis. Then, the reaction solution was poured into 10 mL of water. The aqueous phase was extracted with EA (3 × 15 mL). The combined organic phases were washed with 15 mL of water and 15 mL of brine. After drying over Na_2SO_4 and removal of the solvent in vacuo, the crude products were directly purified by flash chromatography on silica gel to give the desired product.

V. Mechanistic investigations

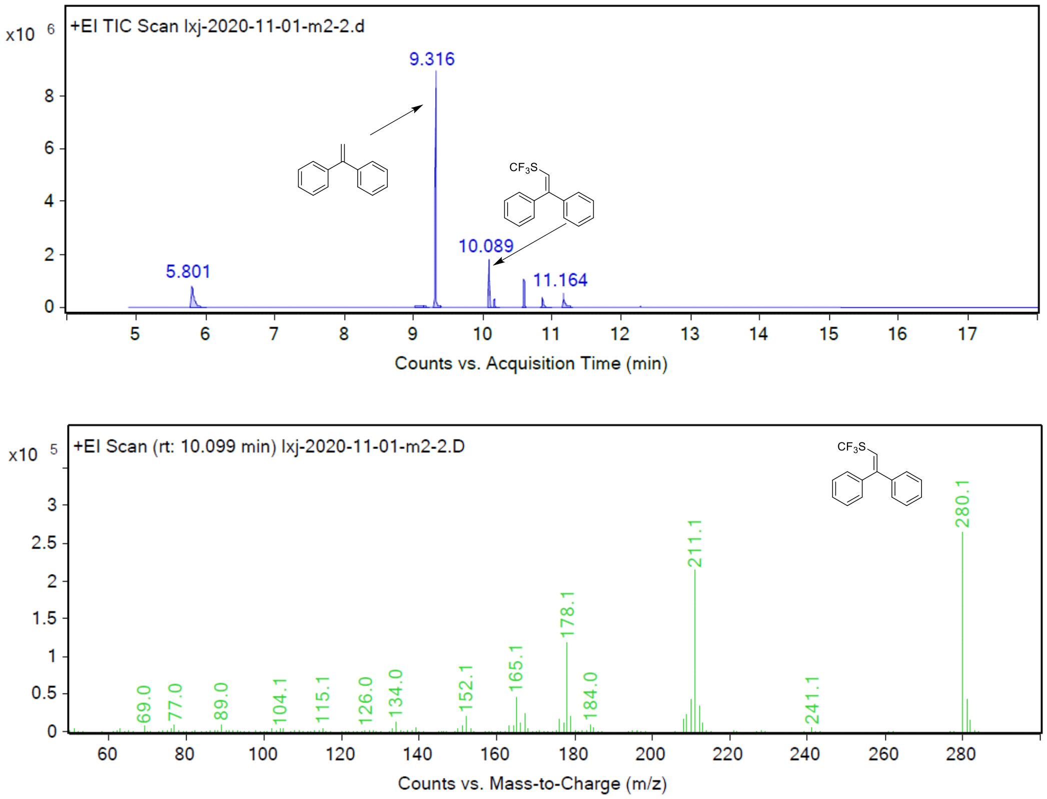

The GC–MS of the reaction mixture

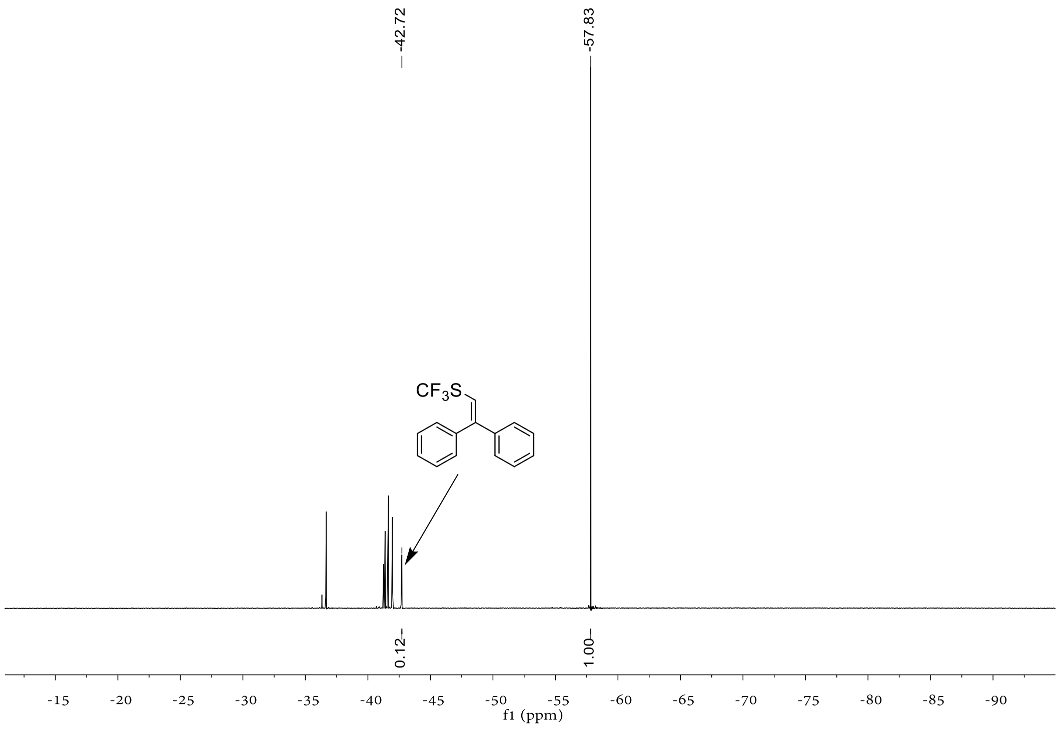

VI. Characterization data

4,4,5,5-Tetramethyl-2-(2-(phenylthio)-3-((trifluoromethyl)thio)propyl)-1,3,2-dioxaborolane (4a)

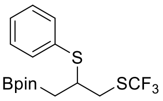

Following the general procedure, **4a** was purified by silica gel chromatography (EA/PE = 1/80); 52.9 mg (70%); yellow oil. ^{1}H NMR (500 MHz, $CDCl_3$) δ 7.51 – 7.45 (m, 2H), 7.36 – 7.28 (m, 3H), 3.56-3.49 (m, *J* = 7.5, 5.6 Hz, 1H), 3.18 (dd, *J* = 13.4, 5.4 Hz, 1H), 3.03 (dd, *J* = 13.4, 7.6 Hz, 1H), 1.36 (dd, *J* = 15.9, 6.9 Hz, 1H), 1.28 (s, 12H), 1.20 (dd, *J* = 15.9, 7.9 Hz, 1H). ^{13}C NMR (126 MHz, $CDCl_3$) δ 133.5, 132.9, 131.1 (q, *J* = 306.2 Hz), 129.0, 127.9, 83.7, 44.8, 36.6 (q, *J* = 1.8 Hz), 24.8, 24.8, 16.8. ^{19}F NMR (376 MHz, $CDCl_3$) δ -40.88. HRMS(ESI) m/z: $[M + Na]^+$ calcd for $C_{16}H_{22}BF_3NaO_2S_2$ 401.0998; found 401.0998.

2-(2-((4-(*tert*-Butyl)phenyl)thio)-3-((trifluoromethyl)thio)propyl)-4,4,5,5-tetramethyl-1,3,2-dioxaborolane(4b)

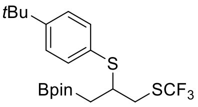

Following the general procedure, **4b** was purified by silica gel chromatography (EA/PE = 1/80); 52.1mg (60%); yellow oil. ^{1}H NMR (400 MHz, $CDCl_3$) δ 7.45 – 7.38 (m, 2H), 7.37 – 7.30 (m, 2H), 3.56 – 3.37 (m, 1H), 3.18 (dd, *J* = 13.3, 5.6 Hz, 1H), 3.04 (dd, *J* = 13.4, 7.4 Hz, 1H), 1.39 – 1.33 (m, 1H), 1.31 (s, 9H), 1.28 (s, 12H), 1.25 – 1.16 (m, 1H). ^{13}C NMR (126 MHz, $CDCl_3$) δ 151.4, 133.8, 131.1 (q, *J* = 306.3 Hz), 128.9, 126.1, 83.6, 44.9, 36.6 (q, *J* = 2.0 Hz), 34.6, 31.2,30.9, 24.8, 24.8, 16.9. ^{19}F NMR (376 MHz, $CDCl_3$) δ -40.85. HRMS(ESI) m/z: $[M + Na]^+$ calcd for $C_{20}H_{30}BF_3NaO_2S_2$ 457.1625; found 457.1626.

4,4,5,5-Tetramethyl-2-(2-(p-tolylthio)-3-((trifluoromethyl)thio)propyl)-1,3,2-dioxaborolane (4c)

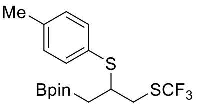

Following the general procedure, **4c** was purified by silica gel chromatography (EA/PE = 1/80); 53.3 mg (68%); yellow oil. ^{1}H NMR (500 MHz,$CDCl_3$ δ 7.38 (d, *J* = 8.1 Hz, 2H), 7.13 (d, *J* = 7.8 Hz, 2H), 3.51 – 3.38 (m, 1H), 3.15 (dd, *J* = 13.3, 5.6 Hz, 1H), 3.02 (dd, *J* = 13.3, 7.4 Hz, 1H), 2.34 (s, 3H), 1.33 (dd, *J* = 15.9, 7.0 Hz, 1H), 1.28 (s, 12H), 1.18 (dd, *J* = 16.0, 8.0 Hz, 1H). ^{13}C NMR (126 MHz, $CDCl_3$) δ 138.2, 134.1, 131.1 (q, *J* = 306.4 Hz), 129.8, 128.9, 83.6, 45.0, 36.6 (q, *J* = 1.8 Hz), 24.8, 24.8, 21.1, 16.7.^{19}F NMR (471 MHz, $CDCl_3$) δ -40.88. HRMS(ESI) m/z: $[M + Na]^+$ calcd for $C_{17}H_{24}BF_3NaO_2S_2$ 416.1155; found 415.1152.

2-(2-((4-Bromophenyl)thio)-3-((trifluoromethyl)thio)propyl)-4,4,5,5-tetramethyl-1,3,2-dioxaborolane (4d)

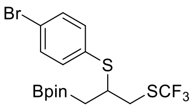

Following the general procedure, **4d** was purified by silica gel chromatography (EA/PE = 1/80); 52.9 mg (58%); yellow oil. ^{1}H NMR (400 MHz, $CDCl_3$) δ 7.45 (d, *J* = 8.5 Hz, 2H), 7.34 (d, *J* = 8.5 Hz, 2H), 3.54 – 3.44 (m, 1H), 3.16 (dd, *J* = 13.5, 5.6 Hz, 1H), 3.03 (dd, *J* = 13.5, 7.5 Hz, 1H), 1.36 (dd, *J* = 16.0, 6.9 Hz, 1H), 1.27 (s, 12H), 1.19 (dd, *J* = 16.0, 8.0 Hz, 1H). ^{13}C NMR (126 MHz, $CDCl_3$) δ 134.9, 134.5, 131.0(q, *J* = 306.2 Hz), 132.2, 122.3, 83.7, 45.1, 36.6 (q, *J* = 1.9 Hz), 24.8, 24.8, 16.8. ^{19}F NMR (376 MHz, $CDCl_3$) δ -40.85. HRMS(ESI) m/z: [M + Na]$^+$ calcd for $C_{16}H_{21}BBrF_3NaO_2S_2$ 479.0104; found 479.0103.

2-(2-((3,5-Dichlorophenyl)thio)-3-((trifluoromethyl)thio)propyl)-4,4,5,5-tetramethyl-1,3,2-dioxaborolane(4e)

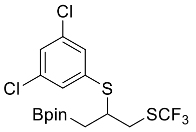

Following the general procedure, **4e** was purified by silica gel chromatography (EA/PE = 1/80); 57.9 mg (65%); yellow oil. ^{1}H NMR (500 MHz, $CDCl_3$) δ 7.33 (d, *J* = 1.9 Hz, 2H), 7.27-7.24 (m, 1H), 3.62-3.55 (m, 1H), 3.21 (dd, *J* = 13.7, 5.4 Hz, 1H), 3.07 (dd, *J* = 13.7, 7.7 Hz, 1H), 1.40 (dd, *J* = 16.1, 6.8 Hz, 1H), 1.27 (d, *J* = 2.4 Hz, 12H), 1.21 (dd, *J* = 16.1, 7.9 Hz, 1H). ^{13}C NMR (126 MHz, $CDCl_3$) δ 137.4, 135.2, 130.9 (q, *J* = 306.5 Hz), 129.8, 127.6, 83.9, 45.0, 36.6 (q, *J* = 1.6 Hz), 24.8, 16.8. ^{19}F NMR (471 MHz, $CDCl_3$) δ -40.79. HRMS(ESI) m/z: [M + Na]$^+$ calcd for $C_{16}H_{20}BCl_2F_3NaO_2S_2$ 469.0219; found 469.0220.

2-(2-((2-Fluorophenyl)thio)-3-((trifluoromethyl)thio)propyl)-4,4,5,5-tetramethyl-1,3,2-dioxaborolane (4f)

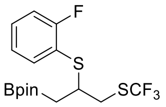

Following the general procedure, **4f** was purified by silica gel chromatography (EA/PE = 1/80); 43.6 mg (55%); yellow oil. ^{1}H NMR (400 MHz, $CDCl_3$) δ 7.53-7.46 (m, 1H), 7.34 – 7.28 (m, 1H), 7.17 – 7.04 (m, 2H), 3.64-3.54 (m, 1H), 3.19 (dd, *J* = 13.6, 5.2 Hz, 1H), 3.04 (dd, *J* = 13.6, 7.6 Hz, 1H), 1.36 (dd, *J* = 16.0, 7.3 Hz, 1H), 1.25 (s, 12H), 1.21 (dd, *J* = 16.1, 7.4 Hz, 1H). ^{13}C NMR (126 MHz, $CDCl_3$) δ 162.7 (d, *J* = 246.8 Hz), 136.1, 131.0 (q, *J* = 306.3 Hz), 130.4 (d, *J* = 8.0 Hz), 124.6 (d, *J* = 4.0 Hz), 119.9 (d, *J* = 18.4 Hz), 116.0 (d, *J* = 23.1 Hz), 83.7, 44.3 (q, *J* = 1.8 Hz), 36.6, 24.8, 24.8, 16.7; ^{19}F NMR (376 MHz, $CDCl_3$) δ -40.94, -106.54- -106.63 (m, *J* = 7.6, 4.4 Hz). HRMS(ESI) m/z: [M + Na]$^+$ calcd for $C_{16}H_{21}BF_4NaO_2S_2$ 419.0904; found 419.0902.

2-(2-((4-Chlorophenyl)thio)-3-((trifluoromethyl)thio)propyl)-4,4,5,5-tetramethyl-1,3,2-dioxaborolane(4g)

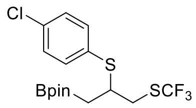

Following the general procedure, **4g** was purified by silica gel chromatography (EA/PE = 1/80); 56.0 mg (68%); yellow oil. ^{1}H NMR (400 MHz, $CDCl_3$) δ 7.44 – 7.37 (m, 2H), 7.32-7.27 (m, 2H), 3.55 – 3.43 (m, 1H), 3.15 (dd, *J* = 13.5, 5.6 Hz, 1H), 3.03 (dd, *J* = 13.4, 7.4 Hz, 1H), 1.35 (dd, *J* = 16.0, 6.9 Hz, 1H), 1.27 (s, 12H), 1.18 (dd, *J* = 15.9, 8.0 Hz, 1H). ^{13}C NMR (126 MHz, $CDCl_3$) δ 134.8, 134.2, 131.4, 131.0 (q, *J* = 306.3 Hz), 129.2, 83.7, 45.2, 36.5 (q, *J* = 1.9 Hz), 24.8, 24.8, 16.8. ^{19}F NMR (376 MHz, $CDCl_3$) δ -40.88. HRMS(ESI) m/z: [M] calcd for $C_{16}H_{22}BClF_3O_2S_2$ 413.0789; found 413.0789.

4,4,5,5-Tetramethyl-2-(3-(phenylthio)-4-((trifluoromethyl)thio)butyl)-1,3,2-dioxaborolane(4h)

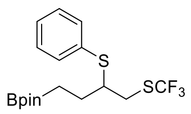

Following the general procedure, **4h** was purified by silica gel chromatography (EA/PE = 1/80); 31.4mg (40%); yellow oil ^{1}H NMR (500 MHz, $CDCl_3$) δ 7.51 – 7.44 (m, 2H), 7.37 – 7.27 (m, 3H), 3.33-3.23 (m, 1H), 3.16 (dd, *J* = 13.5, 5.7 Hz, 1H), 2.99 (dd, *J* = 13.5, 7.9 Hz, 1H), 2.03-1.94 (m, 1H), 1.75-1.63 (m, 1H), 1.27 (s, 12H), 1.16 – 0.99 (m, 2H). ^{13}C NMR (126 MHz, $CDCl_3$) δ 133.2, 133.0, 131.0 (q, *J* = 306.4 Hz), 129.0, 127.7, 83.2, 50.7, 34.9 (q, *J* = 1.8Hz), 27.5, 24.8, 24.8, 8.7. ^{19}F NMR (471 MHz, $CDCl_3$) δ -41.02. HRMS(ESI) m/z: [M+Na] calcd for $C_{17}H_{24}BF_3NaO_2S_2$ 415.1446; found 415.1145.

4-(Phenylthio)-5-((trifluoromethyl)thio)pentyl 4-chlorobenzoate(5a)

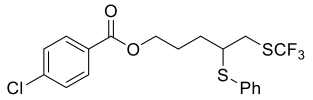

Following the general procedure, **5a** was purified by silica gel chromatography (EA/PE = 1/80); 67.7 mg (78%);colourless oil. ^{1}H NMR (500 MHz, $CDCl_3$) δ 8.01 – 7.92 (m, 2H), 7.49 – 7.38 (m, 4H), 7.34-7.29 (m, *J* = 4.0, 1.7 Hz, 3H), 4.37 (t, *J* = 6.2 Hz,2H), 3.32-3.24 (m, 1H), 3.21 (dd, *J* = 13.6, 5.3 Hz, 1H), 2.94 (dd, *J* = 13.5, 8.3 Hz, 1H), 2.21 – 2.08 (m, 1H), 2.06 – 1.92 (m, 2H), 1.72 – 1.61 (m, 1H). ^{13}C NMR (126 MHz, $CDCl_3$) δ 165.7, 139.4, 133.3, 132.2, 130.9 (q, *J* = 306.4 Hz), 130.9, 129.2, 128.7, 128.6, 128.1, 64.5, 48.1, 35.0 (q, *J* = 1.8 Hz), 29.2, 26.1. ^{19}F NMR (471 MHz, $CDCl_3$) δ -40.84. HRMS(ESI) m/z: $[M + Na]^+$ calcd for $C_{19}H_{18}ClF_3NaO_2S_2$ 457.0281; found 457.0279.

5-(Phenylthio)-6-((trifluoromethyl)thio)hexyl 4-methylbenzoate(5b)

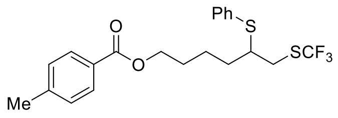

Following the general procedure, **5b** was purified by silica gel chromatography (EA/PE = 1/80); 56.5 mg (66%);colourless oil. ^{1}H NMR (400 MHz, $CDCl_3$) δ 7.97 – 7.90 (m, 2H), 7.47 – 7.40 (m, 2H), 7.36 – 7.27 (m, 3H), 7.26-7.21 (m, 2H), 4.32 (t, *J* = 6.0 Hz, 2H), 3.33 – 3.20 (m, 1H), 3.17 (dd, *J* = 13.4, 5.2 Hz, 1H), 2.94 (dd, *J* = 13.4, 8.1 Hz, 1H), 2.40 (s, 3H), 1.96 – 1.72 (m, 4H), 1.71 – 1.56 (m, 2H). ^{13}C NMR (126 MHz, $CDCl_3$) δ 166.6, 143.5, 133.2, 132.6, 130.9 (q, *J* = 306.4 Hz), 129.5, 129.1, 129.0, 128.0, 127.6, 64.4, 48.3, 35.0 (q, *J* = 1.8 Hz), 32.3, 28.4, 23.4, 21.6. ^{19}F NMR (376 MHz, $CDCl_3$) δ -40.87. HRMS(ESI) m/z: $[M + Na]^+$ calcd for $C_{21}H_{23}F_3NaO_2S_2$ 451.0984; found 451.0983

4-(Phenylthio)-5-((trifluoromethyl)thio)pentyl 3,5-dimethylbenzoate(5c)

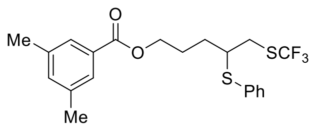

Following the general procedure, **5c** was purified by silica gel chromatography (EA/PE = 1/80); 61.6 mg (72%); colourless oil. ^{1}H NMR (400 MHz, $CDCl_3$) δ 7.66 (s, 2H), 7.48 – 7.41 (m, 2H), 7.33-7.28 (m, 3H), 7.20 (s, 1H), 4.36 (t, *J* = 6.2 Hz, 2H), 3.35-3.26 (m, 1H), 3.21 (dd, *J* = 13.5, 5.3 Hz, 1H), 2.96 (dd, *J* = 13.5, 8.2 Hz, 1H), 2.36 (s, 6H), 2.23 – 2.09 (m, 1H), 2.08 – 1.91 (m, 2H), 1.75 – 1.59 (m, 1H). ^{13}C NMR (126 MHz, $CDCl_3$) δ 166.9, 138.0, 134.6, 133.3, 132.2, 130.9 (q, *J* = 306.4 Hz), 130.0, 129.2, 128.1, 127.3, 64.1, 48.1, 35.0 (q, *J* = 2.1 Hz), 29.2, 26.1, 21.1. ^{19}F NMR (376 MHz, $CDCl_3$) δ -40.85. HRMS(ESI) m/z: $[M + Na]^+$ calcd for $C_{21}H_{23}F_3NaO_2S_2$ 451.0984; found 451.0980.

5-(Phenylthio)-6-((trifluoromethyl)thio)hexyl 2-naphthoate(5d)

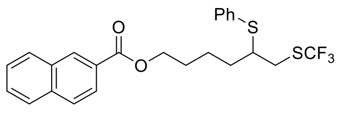

Following the general procedure, **5d** was purified by silica gel chromatography (EA/PE = 1/80); 62.2 mg (67%);colourless oil. ^{1}H NMR (500 MHz, $CDCl_3$) δ 8.63 (s, 1H), 8.10-8.06 (m, 1H), 7.98-7.04 (m, 1H), 7.91-7.86 (m, 2H), 7.62-7.52 (m, 2H), 7.48-7.43 (m, 2H), 7.36 – 7.27 (m, 3H), 4.42 (t, *J* = 6.3 Hz, 2H), 3.32-3.25 (m, 1H), 3.20 (dd, *J* = 13.5, 5.2 Hz, 1H), 2.97 (dd, *J* = 13.5, 8.2 Hz, 1H), 1.98 – 1.79 (m, 4H), 1.75 – 1.65 (m, 2H). ^{13}C NMR (126 MHz, $CDCl_3$) δ 166.7, 135.5, 133.1, 132.6, 132.5, 131.0, 131.0 (q, *J* = 306.4 Hz), 129.3, 129.2, 128.2, 128.1, 128.0, 127.7, 127.5, 126.6, 125.2, 64.7, 48.3, 35.0 (q, *J* = 1.8 Hz), 32.3, 28.4, 23.4. ^{19}F NMR (471 MHz, $CDCl_3$) δ -40.82. HRMS(ESI) m/z: $[M + Na]^+$ calcd for $C_{24}H_{23}F_3NaO_2S_2$ 487.0984; found 487.0986.

(5-(3-Bromophenoxy)-1-((trifluoromethyl)thio)pentan-2-yl)(phenyl)sulfane(5e)

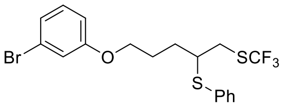

Following the general procedure, **5e** was purified by silica gel chromatography (EA/PE = 1/80); 54.9mg (61%); colourless oil. ^{1}H NMR (500 MHz, CDCl$_3$) δ 7.51 – 7.43 (m, 2H), 7.38 – 7.29 (m, 3H), 7.14 (t, *J* = 8.0 Hz, 1H), 7.11 – 7.03 (m, 2H), 6.85-6.80 (m, 1H), 3.99 (t, *J* = 6.0 Hz, 2H), 3.35-3.25 (m, 1H), 3.20 (dd, *J* = 13.5, 5.4 Hz, 1H), 2.97 (dd, *J* = 13.5, 8.1 Hz,1H), 2.20-2.09 (m, 1H), 2.07 – 1.92 (m, 2H), 1.80 – 1.63 (m, 1H). ^{13}C NMR (126 MHz, CDCl$_3$) δ 159.6, 133.3, 132.4, 130.9 (q, *J* = 306.5 Hz), 130.5, 129.2, 128.1, 123.8, 122.8, 117.7, 113.5, 67.5, 48.3, 35.1 (q, *J* = 1.8 Hz), 29.4, 26.5. ^{19}F NMR (376 MHz, CDCl$_3$) δ -40.87. HRMS(ESI) m/z: [M + Na]$^+$ calcd for $C_{18}H_{18}BrF_3NaOS_2$ 472.9827; found 472.9826.

2-Phenyl-3-((4-(phenylthio)-5-((trifluoromethyl)thio)pentyl)oxy)-4H-chromen-4-one(5f)

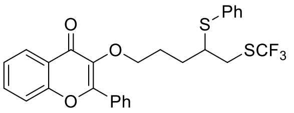

Following the general procedure, **5f** was purified by silica gel chromatography (EA/PE = 1/10); 61.9 mg (65%); colourless oil. ^{1}H NMR (400 MHz, CDCl$_3$) δ 8.27 (dd, *J* = 8.0, 1.7 Hz, 1H), 8.13 – 8.04 (m, 2H), 7.72-7.66 (m, 1H), 7.57 – 7.48 (m, 4H), 7.44-7.38 (m, 3H), 7.36 – 7.27 (m, 3H), 4.12-4.07 (m, 2H), 3.31 – 3.20 (m, 1H), 3.10 (dd, *J* = 13.3, 5.6 Hz, 1H), 2.92 (dd, *J* = 13.4, 7.7 Hz, 1H), 2.11 – 1.99 (m, 1H), 1.97 – 1.81 (m, 2H), 1.65 – 1.56 (m, 1H). ^{13}C NMR (101 MHz, CDCl$_3$) δ 175.1, 156.0, 155.3, 140.4, 133.4, 133.2, 132.6, 131.0 (q, *J* = 306.3 Hz), 131.0, 130.7, 129.1, 128.7, 128.4, 127.9, 125.8, 124.7, 124.2, 118.0, 72.0, 48.3, 35.1 (q, *J* = 2.3 Hz), 29.6, 27.5. ^{19}F NMR (376 MHz, CDCl$_3$) δ -40.92. HRMS(ESI) m/z: [M + Na]$^+$ calcd for $C_{27}H_{23}F_3NaO_3S_2$539.0933; found 539.0930

(4-(3-Methoxyphenoxy)-1-((trifluoromethyl)thio)butan-2-yl)(phenyl)sulfane(5g)

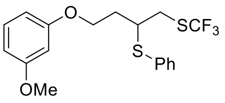

Following the general procedure, **5g** was purified by silica gel chromatography (EA/PE = 1/40); 52mg (67%); colourless oil. ^{1}H NMR (500 MHz, CDCl$_3$) δ 7.52 – 7.45 (m, 2H), 7.39 – 7.31 (m, 3H), 7.22 (t, *J* = 8.2 Hz, 1H), 6.58-6.53 (m, 2H), 6.50 (t, *J* = 2.4 Hz, 1H), 4.30 – 4.17 (m, 2H), 3.83 (s, 3H), 3.65-3.55 (m, 1H), 3.26 (dd, *J* = 13.6, 5.5 Hz, 1H), 3.11 (dd, *J* = 13.5, 7.9 Hz, 1H), 2.39-2.30 (m 1H), 2.07-1.98 (m, 1H). ^{13}C NMR (126 MHz, CDCl$_3$) δ 160.8, 159.8, 133.2, 132.4, 131.0 (q, *J* = 306.5 Hz), 129.9, 129.2, 128.1, 106.7, 106.5, 101.0, 64.7, 55.3, 45.6, 35.2 (q, *J* = 1.8 Hz), 32.5. ^{19}F NMR (471 MHz, CDCl$_3$) δ -40.90. HRMS(ESI) m/z: [M + Na]$^+$ calcd for $C_{18}H_{19}F_3NaO_2S_2$461.0671; found 411.0670

Phenyl(4-phenyl-1-((trifluoromethyl)thio)butan-2-yl)sulfane(5h)

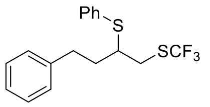

Following the general procedure, **5h** was purified by silica gel chromatography (EA/PE = 1/80); 53.4 mg (78%); colourless oil. ^{1}H NMR (400 MHz, $CDCl_3$) δ 7.47 – 7.41 (m, 2H), 7.36-7.28 (m, 5H), 7.24-7.19 (m,3H), 3.26 – 3.13 (m, 2H), 3.01-2.93 (m, 2H), 2.88-2.77 (m, 1H), 2.22-2.09 (m, 1H), 1.92-1.80 (m, 1H). ^{13}C NMR (101 MHz, $CDCl_3$ δ 140.8, 133.2, 132.5, 130.9 (q, J = 306.5 Hz), 129.2, 128.5, 128.4, 128.0, 126.2, 47.6, 35.1, 34.2, 32.8. ^{19}F NMR (376 MHz, $CDCl_3$) δ -40.91. HRMS(ESI) m/z: $[M + Na]^+$ calcd for $C_{17}H_{17}F_3NaS_2$ 365.0616; found 365.0615.

(4-Methoxyphenyl)(4-phenyl-1-((trifluoromethyl)thio)butan-2-yl)sulfane(5i)

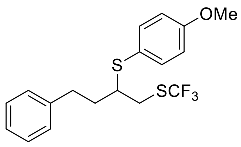

Following the general procedure, **5i** was purified by silica gel chromatography (EA/PE = 1/80); 52.8 mg (71%); colourless oil. ^{1}H NMR (500 MHz, $CDCl_3$) δ 7.44 – 7.40 (m, 2H), 7.34 – 7.29 (m, 2H), 7.25 – 7.21 (m, 3H), 6.90 – 6.86 (m, 2H), 3.82 (s, 3H), 3.13 (dd, J = 12.9, 5.6 Hz, 1H), 3.08 – 3.01 (m, 1H), 3.01 – 2.91 (m, 2H), 2.88 – 2.79 (m, 1H), 2.12-2.03 (m, 1H), 1.87-1.77 (m, 1H). ^{13}C NMR (126 MHz, $CDCl_3$) δ 160.1, 140.9, 136.4, 131.0 (q, J = 306.4 Hz), 128.5, 128.4, 126.1, 122.1, 114.7, 55.3, 48.3, 35.0 (q, J = 1.8 Hz), 34.2, 32.9. ^{19}F NMR (471 MHz, $CDCl_3$) δ -40.96. HRMS(ESI) m/z: $[M + Na]^+$ calcd for $C_{18}H_{19}F_3NaOS_2$ 395.0721; found 395.0717.

2-(4-(Phenylthio)-5-((trifluoromethyl)thio)pentyl)isoindoline-1,3-dione(5j)

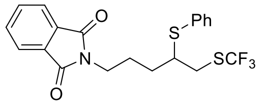

Following the general procedure, **5j** was purified by silica gel chromatography (EA/PE = 1/80); 57.8 mg (68%); colourless oil. ^{1}H NMR (400 MHz, $CDCl_3$) δ 7.88-7.82 (m, 2H), 7.76-7.70 (m, 2H), 7.49 – 7.39 (m, 2H), 7.34 – 7.19 (m, 3H), 3.73 (t, J = 6.9 Hz, 2H), 3.30-3.20 (m, 1H), 3.14 (dd, J = 13.5, 5.5 Hz, 1H), 2.93 (dd, J = 13.5, 8.0 Hz, 1H), 2.17 – 1.99 (m, 1H), 1.95 – 1.78 (m, 2H), 1.61 – 1.49 (m, 1H). ^{13}C NMR (126 MHz, $CDCl_3$) δ 168.4, 134.0, 133.5, 132.2, 132.0, 130.9 (q, J = 306.4 Hz), 129.2, 128.1, 123.3, 48.2, 37.4, 35.0 (q, J = 2.1 Hz), 29.9, 25.9. ^{19}F NMR (376 MHz, $CDCl_3$) δ -40.94. HRMS(ESI) m/z: $[M + Na]^+$ calcd for $C_{20}H_{18}F_3NNaO_2S_2$ 448.0623; found 448.0624.

5-(Phenylthio)-6-((trifluoromethyl)thio)hexyl 4-methylbenzenesulfonate (5k)

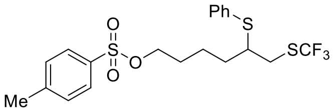

Following the general procedure, **5k** as purified by silica gel chromatography (EA/PE = 1/8); 44.5 mg (48%); colourless oil. ^{1}H NMR (400 MHz, $CDCl_3$) δ 7.80 (d, J = 8.3 Hz, 2H), 7.44 – 7.38 (m, 2H), 7.38 – 7.29 (m, 5H), 4.05 (t, J = 6.0 Hz, 2H), 3.21 – 3.06 (m, 2H), 2.96 – 2.81 (m, 1H), 2.44 (s, 3H), 1.82 – 1.61

(m, 4H), 1.55-1.45 (m, 2H). ^{13}C NMR (126 MHz, $CDCl_3$) δ 144.8, 133.1, 133.0, 132.5, 130.9 (q, *J* = 306.3 Hz), 129.8, 129.2, 128.0, 127.9, 70.0, 48.2, 34.9 (q, *J* = 3.3, 1.5 Hz), 32.0, 28.5, 22.8, 21.6. ^{19}F NMR (376 MHz, $CDCl_3$) δ -40.94. ^{19}F NMR (376 MHz, $CDCl_3$) δ -40.87. HRMS(ESI) m/z: $[M + Na]^+$ calcd for $C_{20}H_{23}F_3NaO_3S_3$ 487.0654; found 487.0651.

(8*R*,9*S*,10*R*,13*S*,14*S*)-10,13-Dimethyl-3-oxo-6,7,8,9,10,11,12,13,14,15,16,17-dodecahydro-3*H*-cyclopenta[*a*]phenanthren-17-yl 10-(phenylthio)-11-((trifluoromethyl)thio)undecanoate (5l)

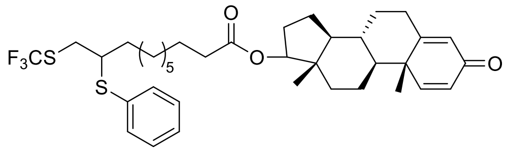

Following the general procedure, **5l** was purified by silica gel chromatography (EA/PE = 1/40); 59.6 mg (45%); colourless oil. ^{1}H NMR (400 MHz, $CDCl_3$) δ 7.47 – 7.23 (m, 4H), 7.04 (d, *J* = 10.2 Hz, 1H), 6.23 (dd, *J* = 10.1, 1.9 Hz, 1H), 6.07 (t, *J* = 1.6 Hz, 1H), 4.64 – 4.55 (m, 1H), 3.29-3.20 (m, 1H), 3.16 (dd, *J* = 13.4, 5.2 Hz, 1H), 2.94 (dd, *J* = 13.4, 8.1 Hz, 1H), 2.53-2.43 (m, 1H), 2.40-2.35 (m, 1H), 2.30 (t, *J* = 7.5 Hz, 2H), 2.23 – 2.11 (m, 1H), 2.00 – 1.91 (m, 1H), 1.86 – 1.44 (m, 13H), 1.42 – 1.35 (m, 1H), 1.31 (s, 7H), 1.26 (s, 1H), 1.23 (s, 3H), 1.22-1.16 (m, 1H), 1.12 – 0.98 (m, 3H), 0.87 (s, 3H). ^{13}C NMR (126 MHz, $CDCl_3$) δ 186.3, 173.7, 168.8, 155.6, 132.9, 132.9, 131.0 (q, *J* = 306.5 Hz), 129.1, 127.8, 127.5, 123.9, 82.0, 52.1, 49.8, 48.3, 43.5, 42.7, 36.5, 35.3, 35.0 (q, *J* = 1.7 Hz), 34.4, 33.0, 32.7, 32.6, 29.2, 29.1, 29.1, 29.0, 27.4, 26.6, 25.0, 23.6, 22.3, 18.7, 12.1. ^{19}F NMR (376 MHz, $CDCl_3$) δ -40.88. HRMS(ESI) m/z: $[M + H]^+$ calcd for $C_{37}H_{50}F_3O_3S_2$ 663.3148; found 663.3147.

(3a*S*,5*S*,6*R*,6a*S*)-5-((S)-2,2-Dimethyl-1,3-dioxolan-4-yl)-2,2-dimethyltetrahydro-furo[2,3-*d*][1,3]dioxol-6-yl 4-(phenylthio)-5-((trifluoromethyl)thio)pentanoate (5m)

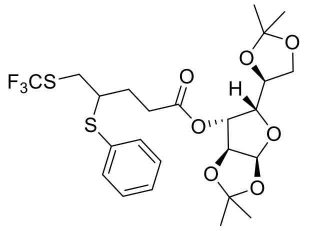

Following the general procedure, **5m** was purified by silica gel chromatography (EA/PE = 1/40); 80.6 mg (73%); colourless oil. ^{1}H NMR (400 MHz, $CDCl_3$, the mixture of diastereomers) δ 7.47-7.42 (m, 2H), 7.37 – 7.30 (m, 3H), 5.90-5.85 (m, 1H), 5.32-5.25 (m, 1H), 4.53-4.44 (m, 1H), 4.25-4.17 (m 2H), 4.12 – 4.00 (m, 2H), 3.35-3.25 (m,1H), 3.23-3.15(m,1H), 3.00-2.88 (m, 1H), 2.77-2.60 (m, 2H), 2.29 – 2.16 (m, 1H), 1.93 – 1.75 (m, 1H), 1.52 (s, 3H), 1.44-1.38 (m,, 3H), 1.34-1.27 (m, 6H). ^{19}F NMR (376 MHz, $CDCl_3$) δ -40.92, -40.94. ^{13}C NMR (126 MHz, $CDCl_3$, the mixture of diastereomers) δ 171.26, 133.48 & 133.54, 131.99 & 131.70, 130.82 (q, *J*=307.4Hz), 130.80 (q, *J*=307.4Hz), 129.25, 128.33 & 128.23, 112.26, 109.35, 105.01, 83.27, 79.77 & 79.76, 76.22 & 76.21, 72.40 & 72.38, 67.28 & 67.26, 47.88, 35.14-34.98 (m, the carbon of α-SCF_3), 31.43 & 31.35, 27.80 & 27.66, 26.84 & 26.83, 26.67, 26.14 & 26.13, 25.19 & 25.17. HRMS(ESI) m/z: $[M + Na]^+$ calcd for $C_{24}H_{31}F_3NaO_7S_2$ 575.1356; found 575.1349.

VII. Spectral data

4,4,5,5-Tetramethyl-2-(2-(phenylthio)-3-((trifluoromethyl)thio)propyl)-1,3,2-dioxaborolane (4a)

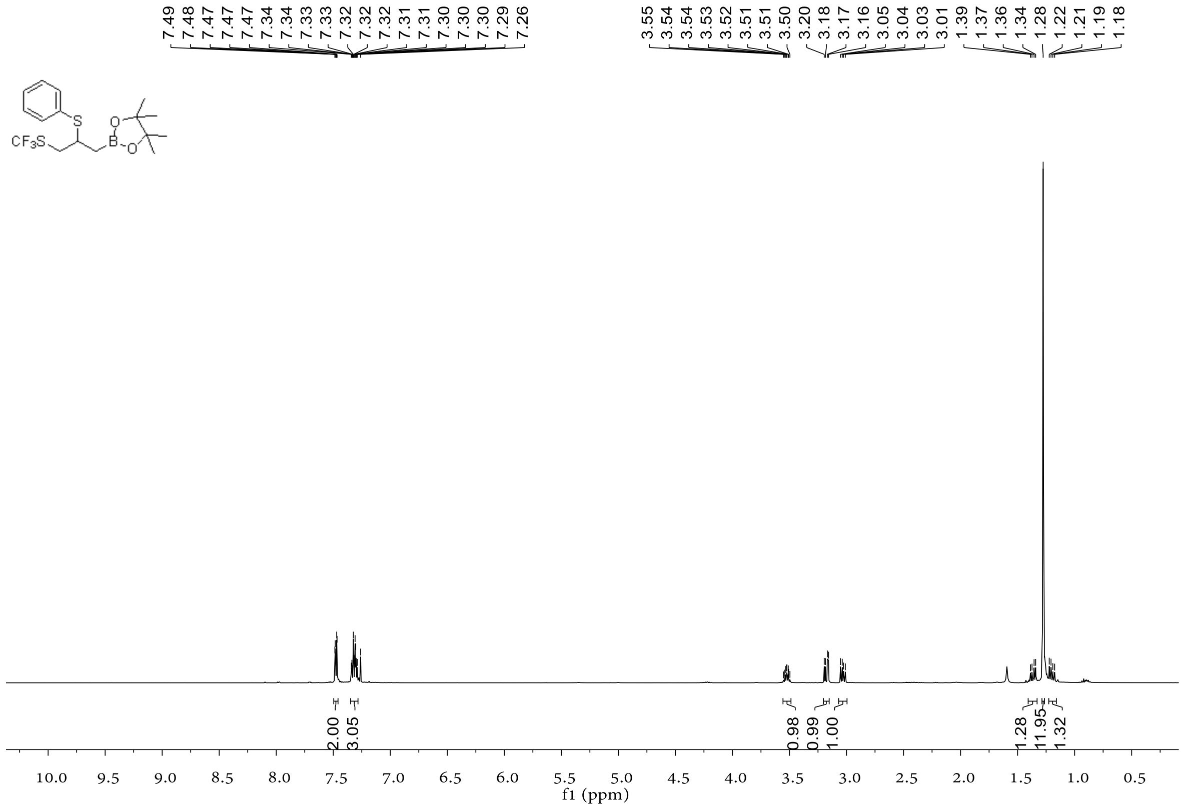

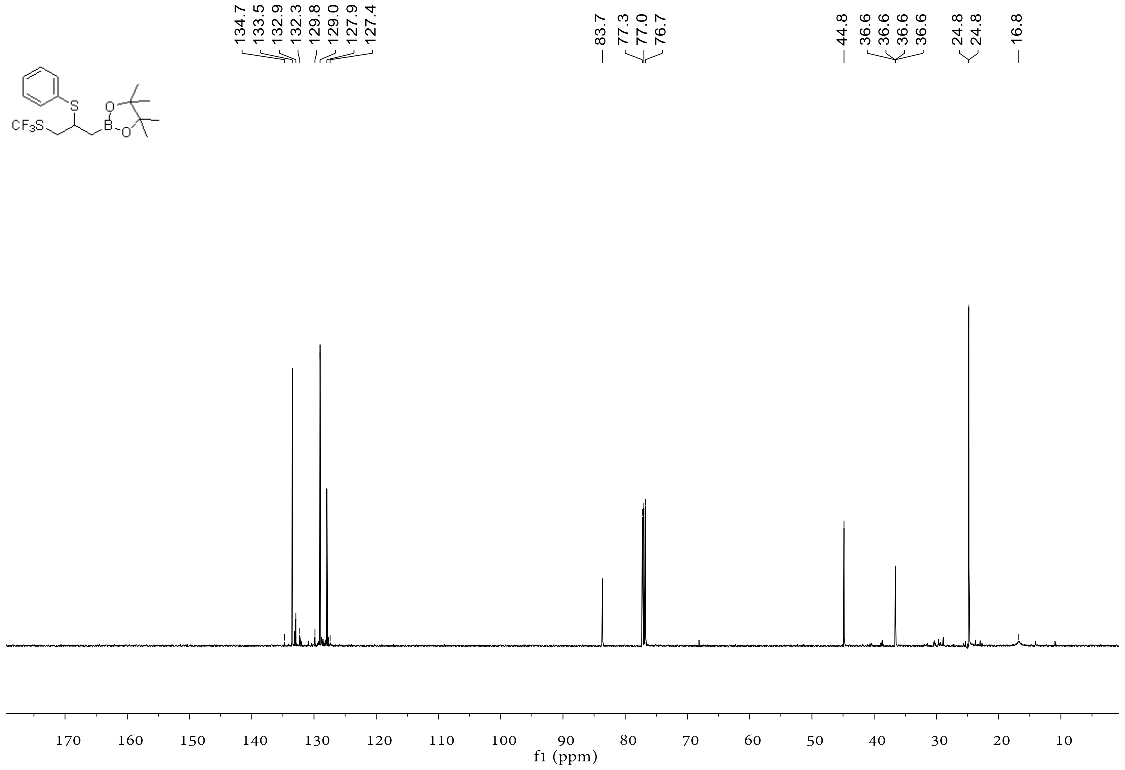

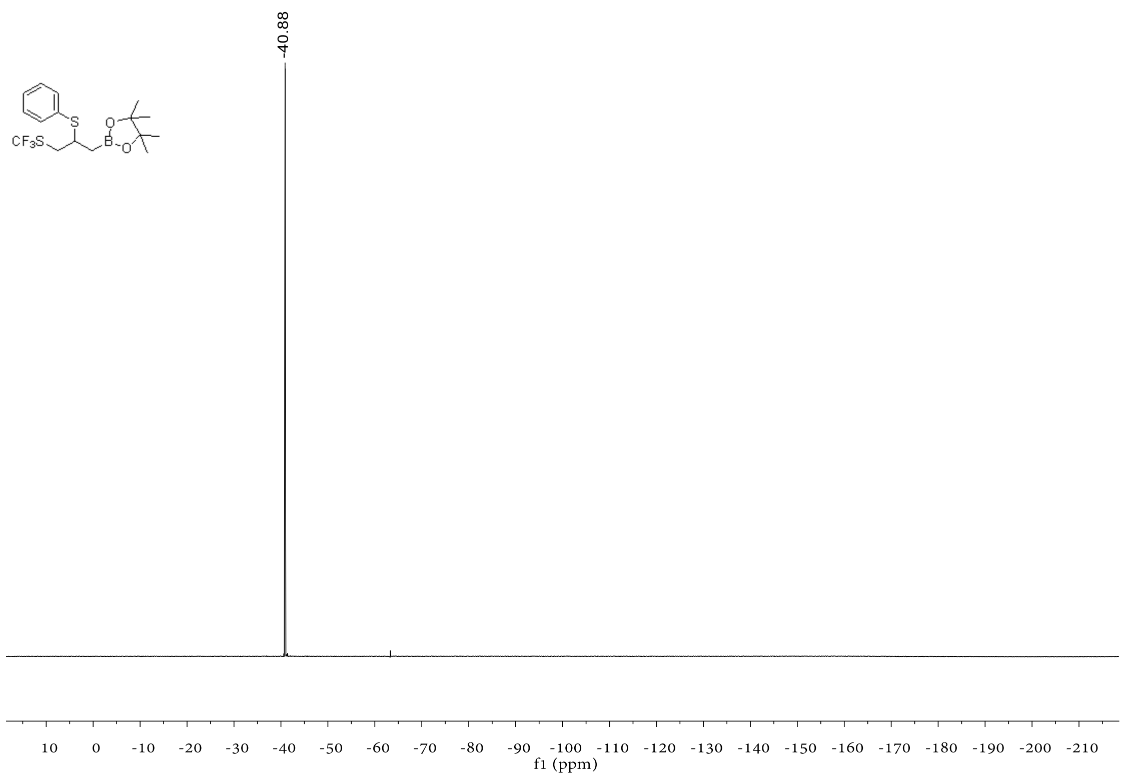

2-(2-((4-(*tert*-Butyl)phenyl)thio)-3-((trifluoromethyl)thio)propyl)-4,4,5,5-tetramethyl-1,3,2-dioxaborolane (4b)

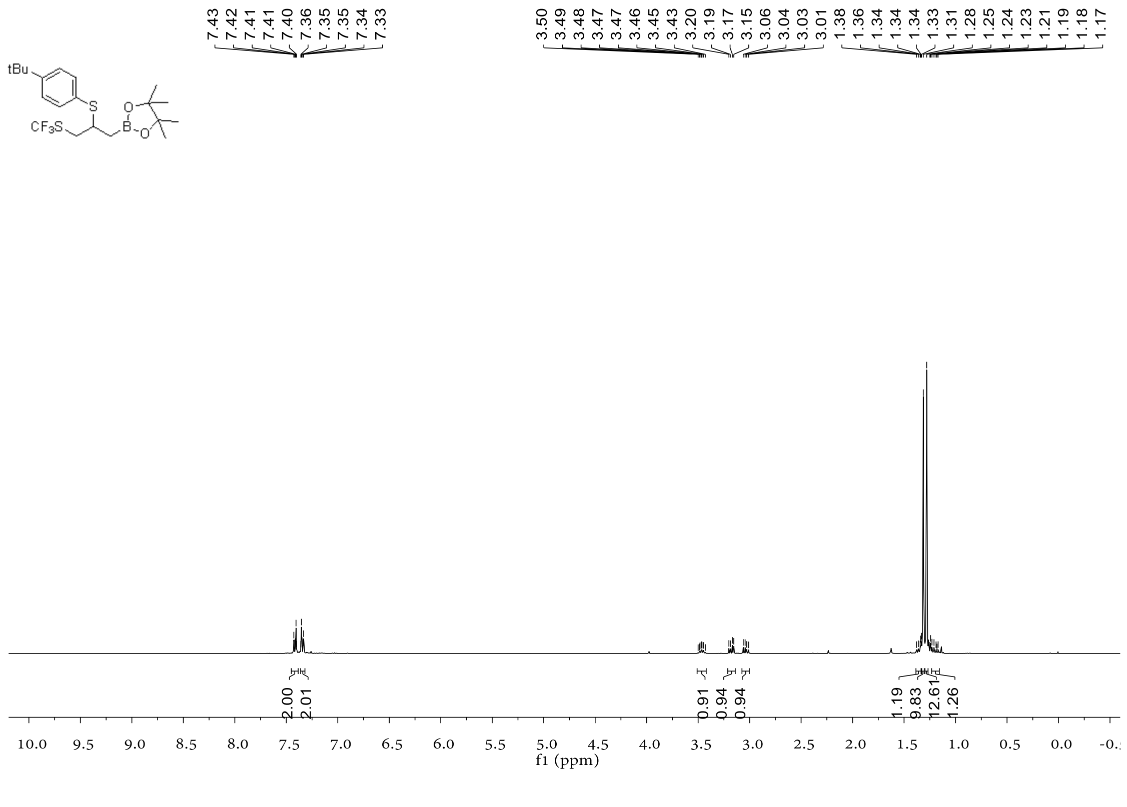

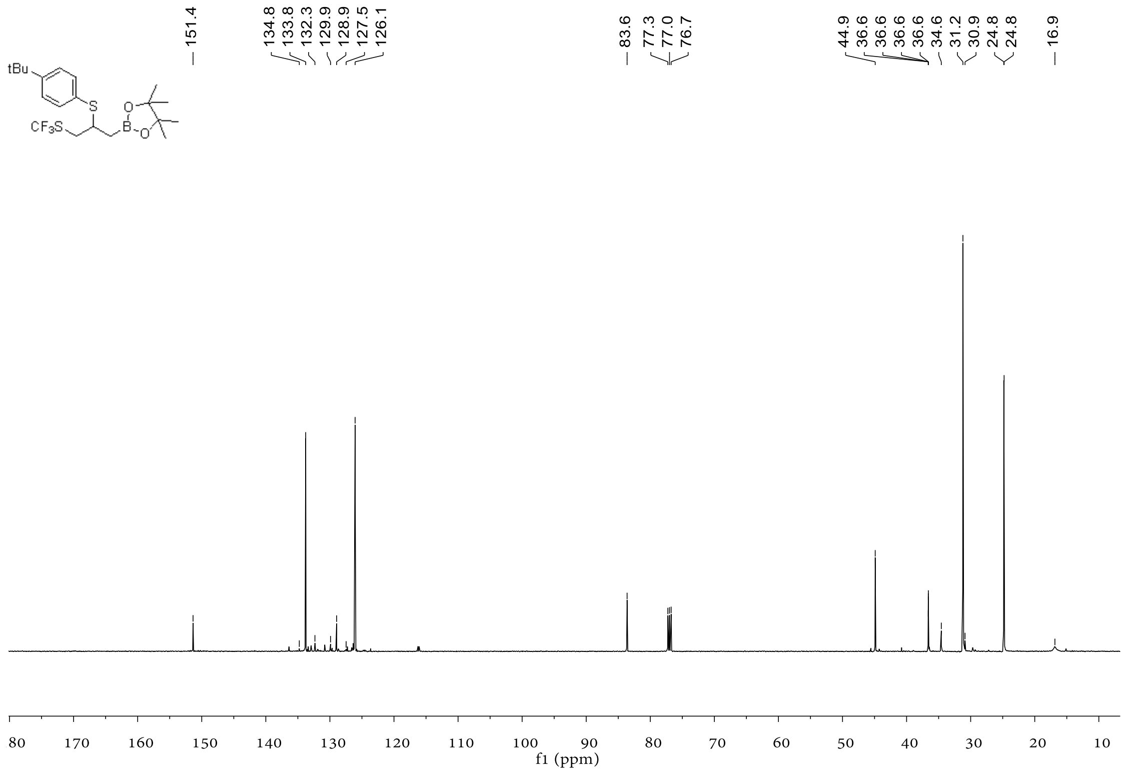
151.4
134.8
133.8
132.3
129.9
128.9
127.5
126.1
83.6
77.3
77.0
76.7
44.9
36.6
36.6
36.6
34.6
31.2
30.9
24.8
24.8
16.9
tBu
S
O
B
O
CF3S
80 170 160 150 140 130 120 110 100 90 80 70 60 50 40 30 20 10
f1 (ppm)

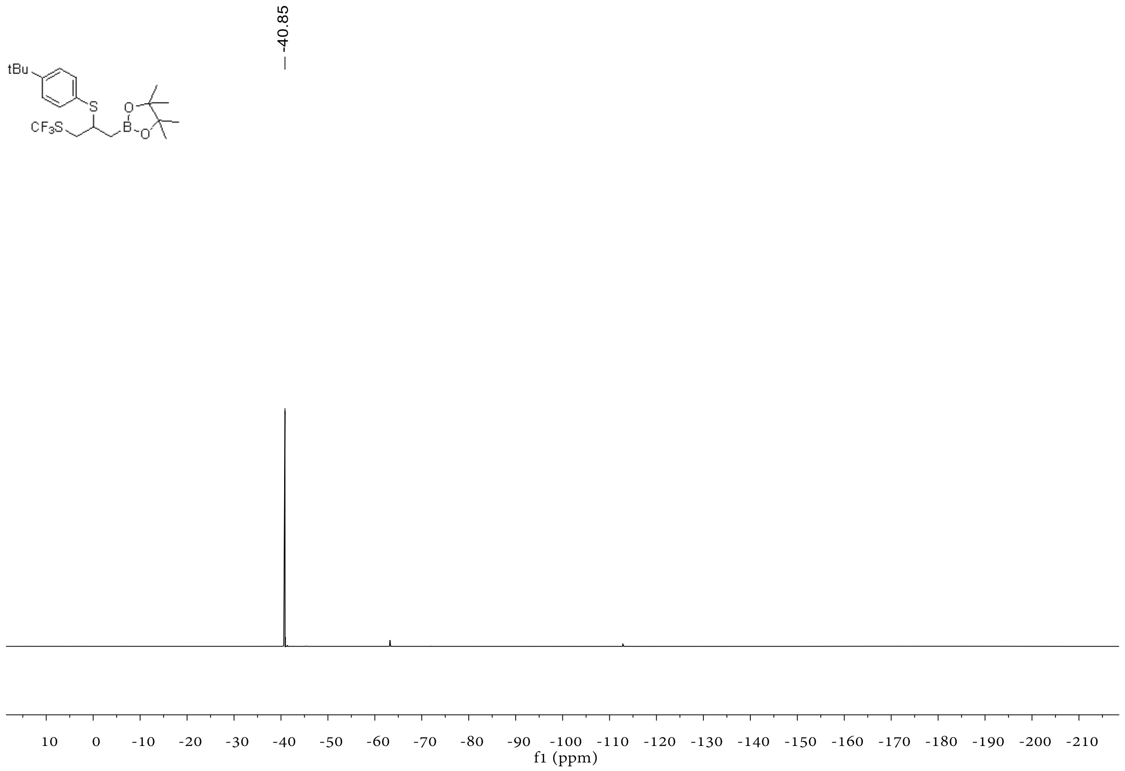
-40.85
tBu
S
O
B
O
CF3S
10 0 -10 -20 -30 -40 -50 -60 -70 -80 -90 -100 -110 -120 -130 -140 -150 -160 -170 -180 -190 -200 -210
f1 (ppm)

4,4,5,5-Tetramethyl-2-(2-(*p*-tolylthio)-3-((trifluoromethyl)thio)propyl)-1,3,2-dioxaborolane (4c)

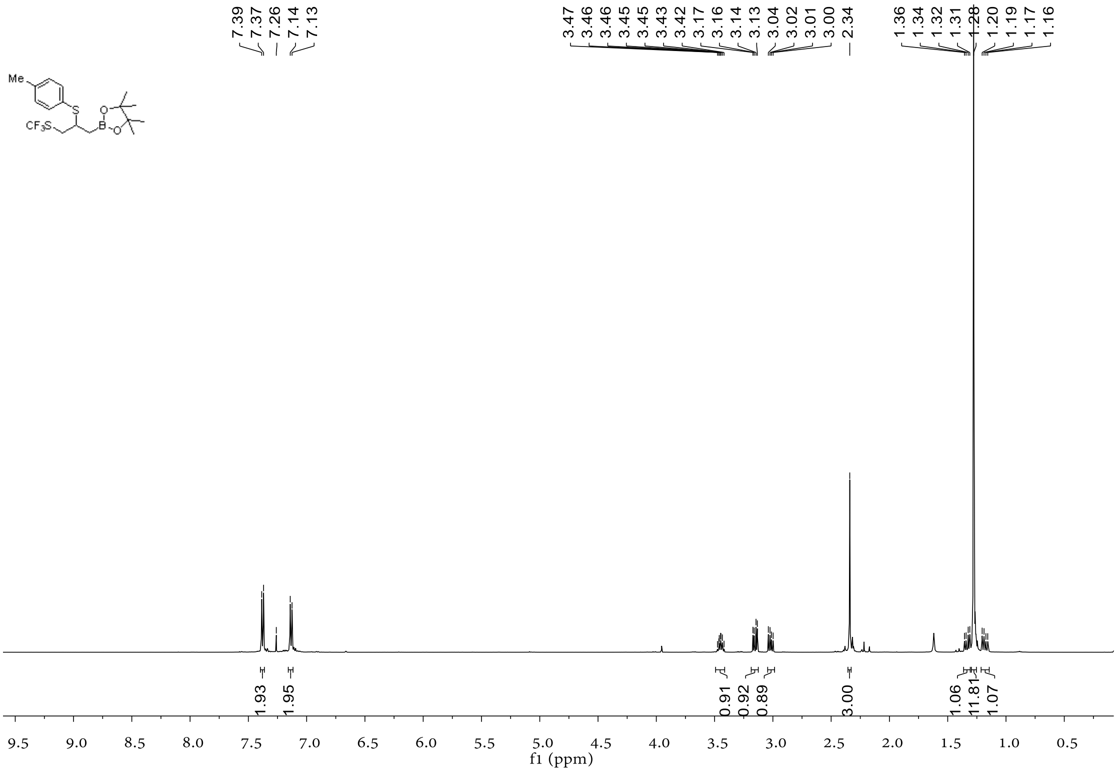

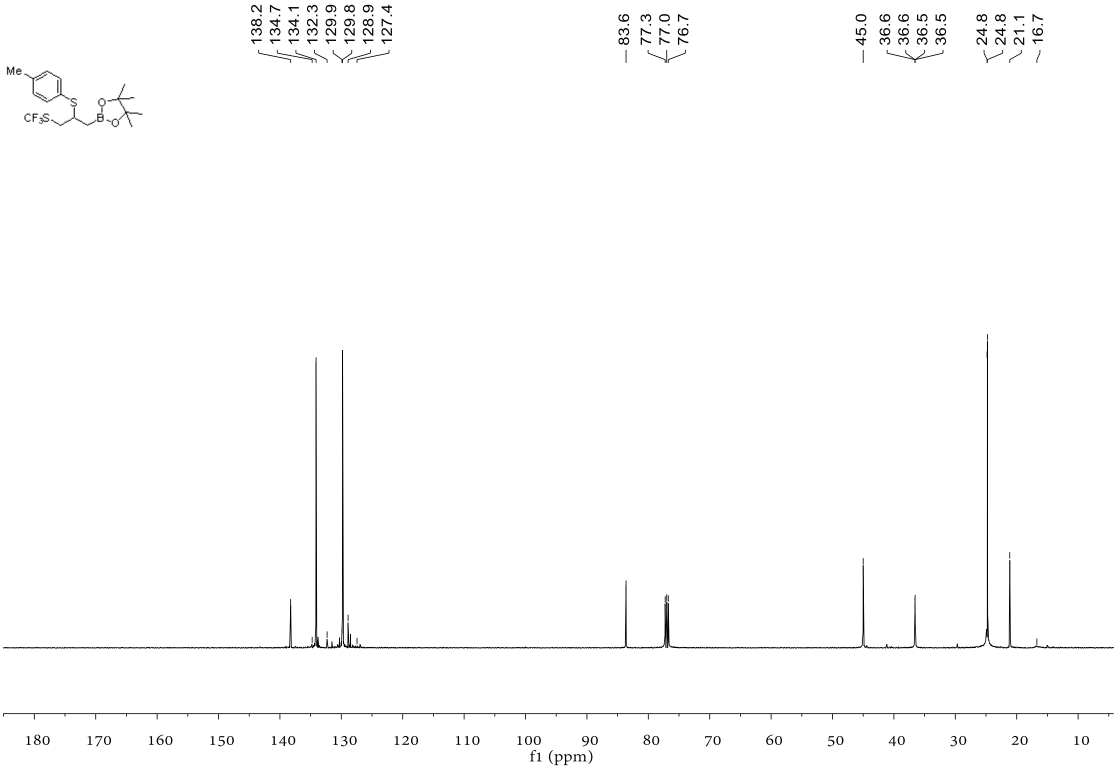

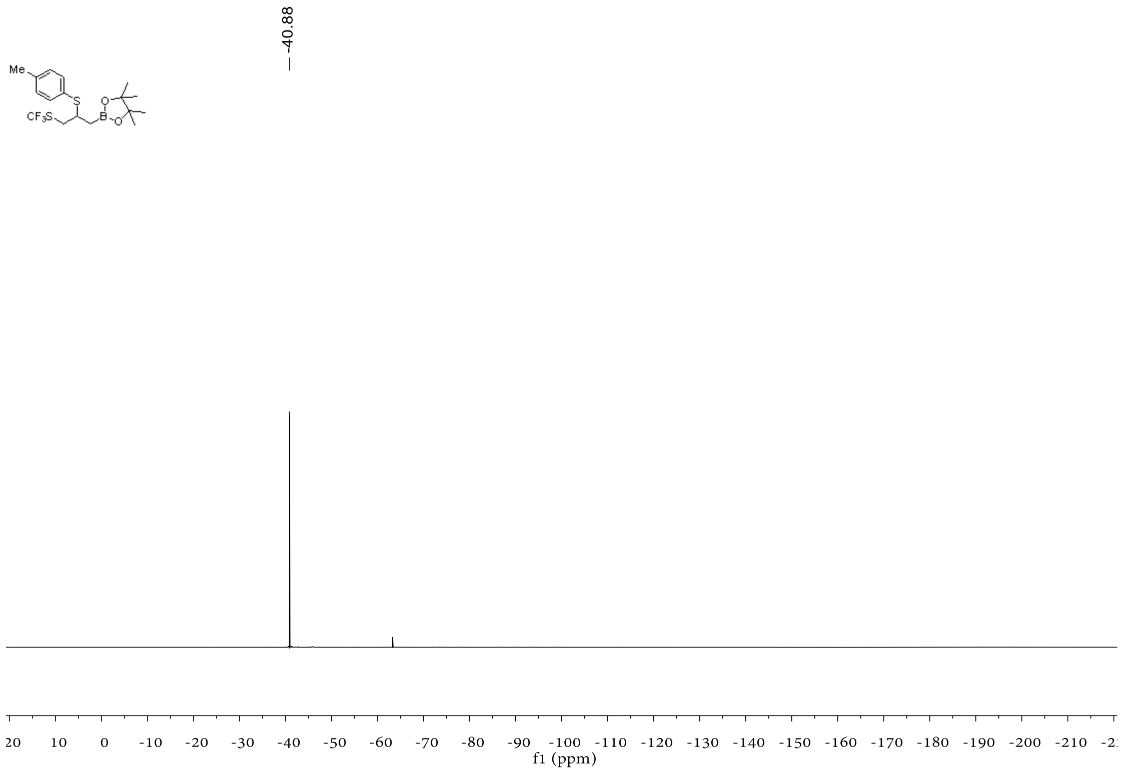

2-(2-((4-Bromophenyl)thio)-3-((trifluoromethyl)thio)propyl)-4,4,5,5-tetramethyl-1,3,2-dioxaborolane (4d)

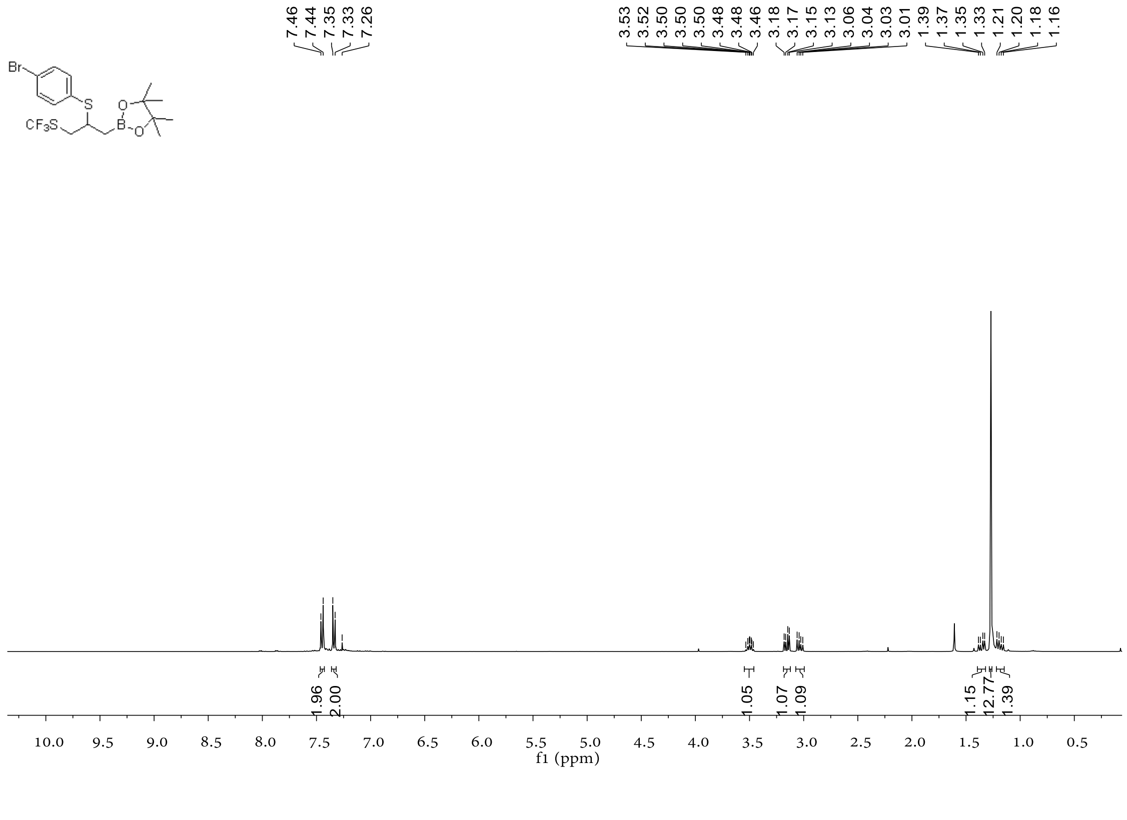

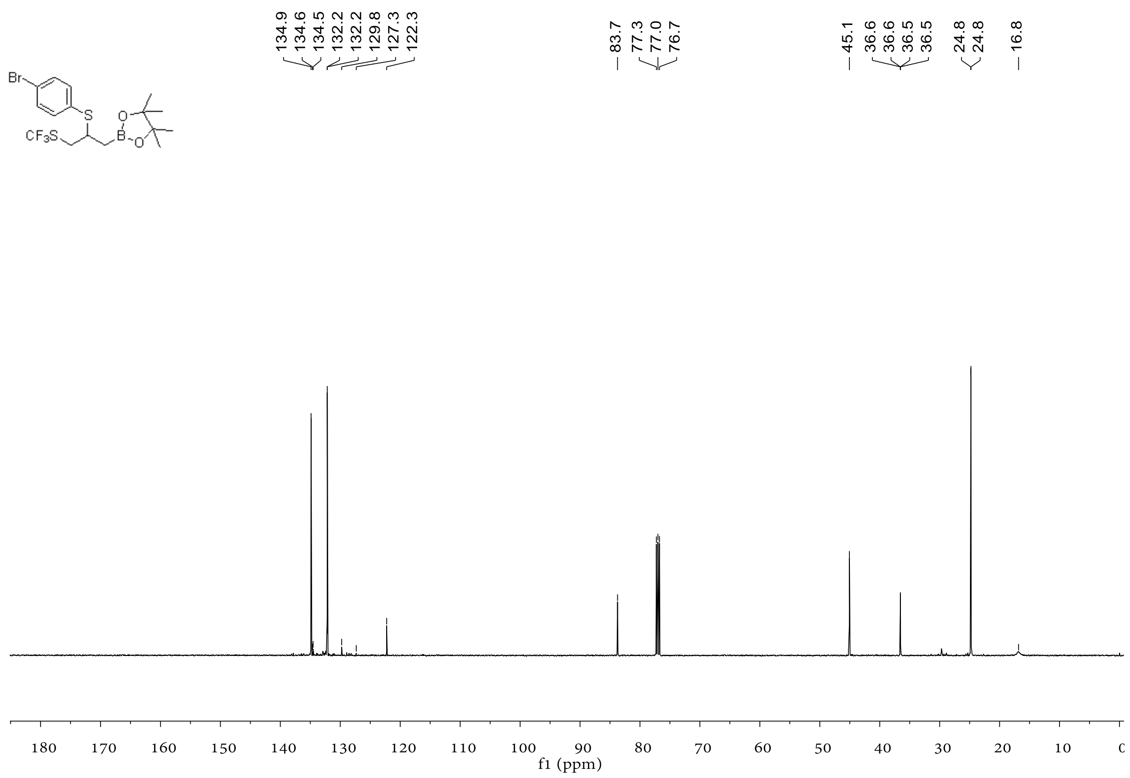

134.9
134.6
134.5
132.2
132.2
129.8
127.3
122.3
83.7
77.3
77.0
76.7
45.1
36.6
36.6
36.5
36.5
24.8
24.8
16.8
180 170 160 150 140 130 120 110 100 90 80 70 60 50 40 30 20 10 0
f1 (ppm)

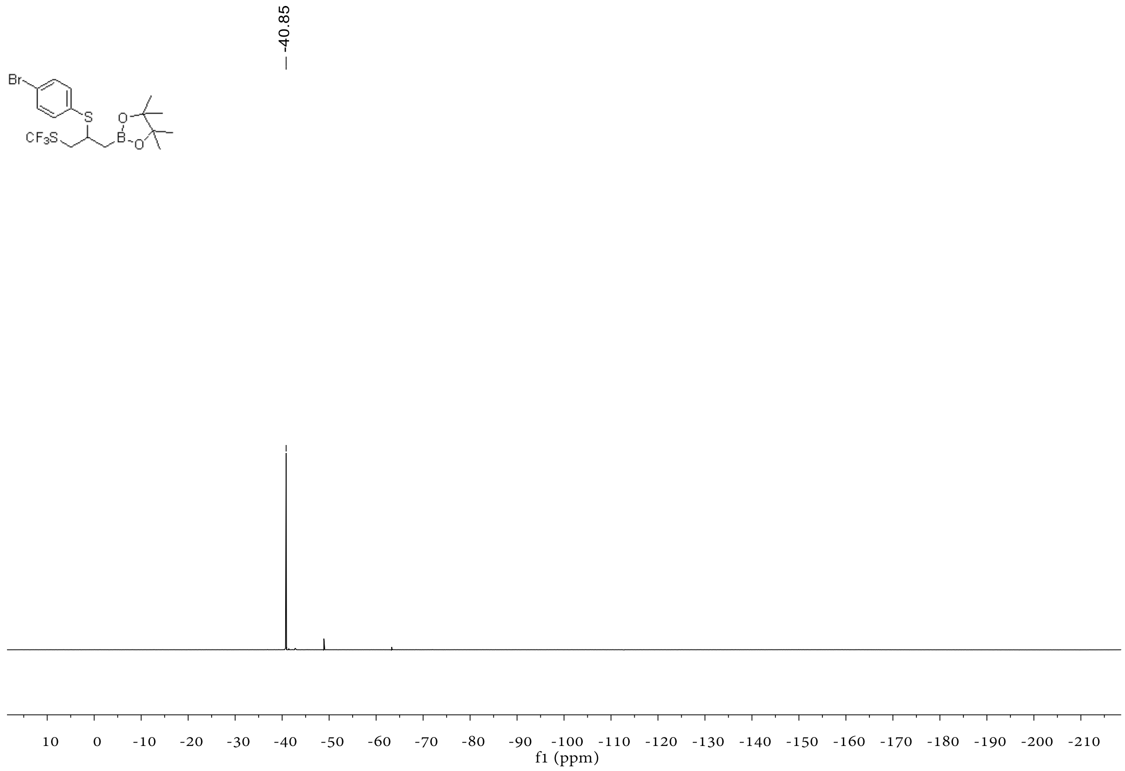

-40.85
10 0 -10 -20 -30 -40 -50 -60 -70 -80 -90 -100 -110 -120 -130 -140 -150 -160 -170 -180 -190 -200 -210
f1 (ppm)

2-(2-((3,5-Dichlorophenyl)thio)-3-((trifluoromethyl)thio)propyl)-4,4,5,5-tetramethyl-1,3,2-dioxaborolane(4e)

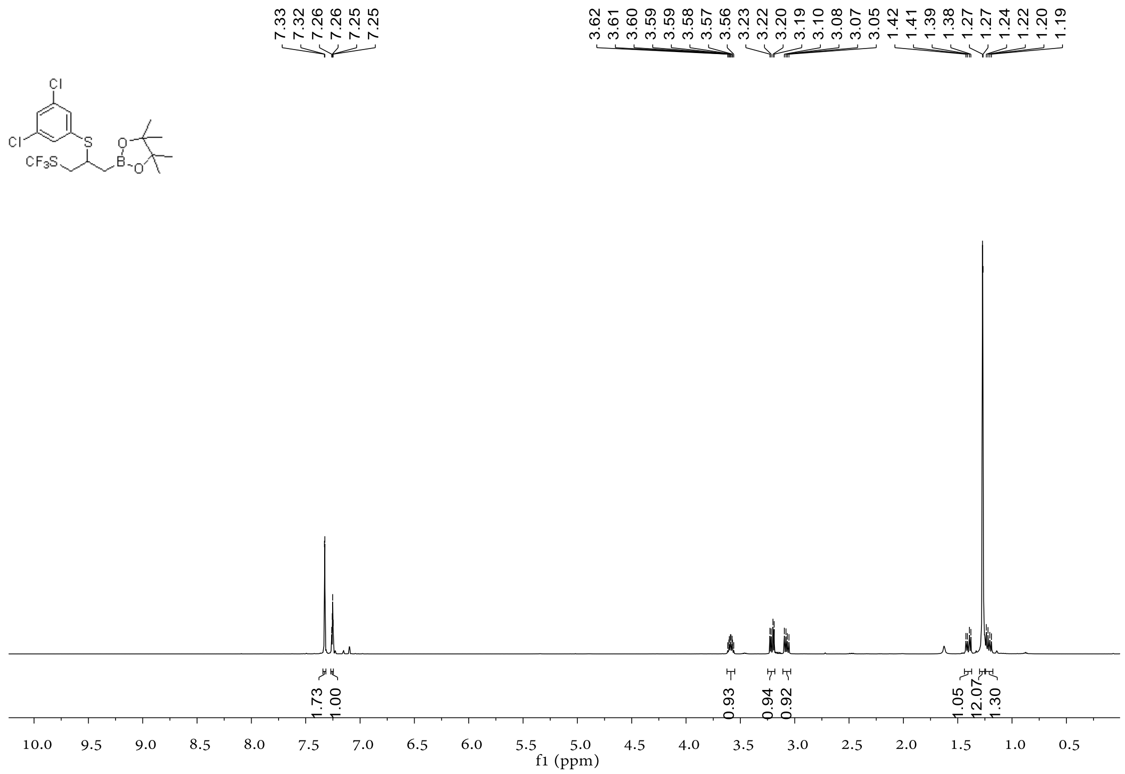

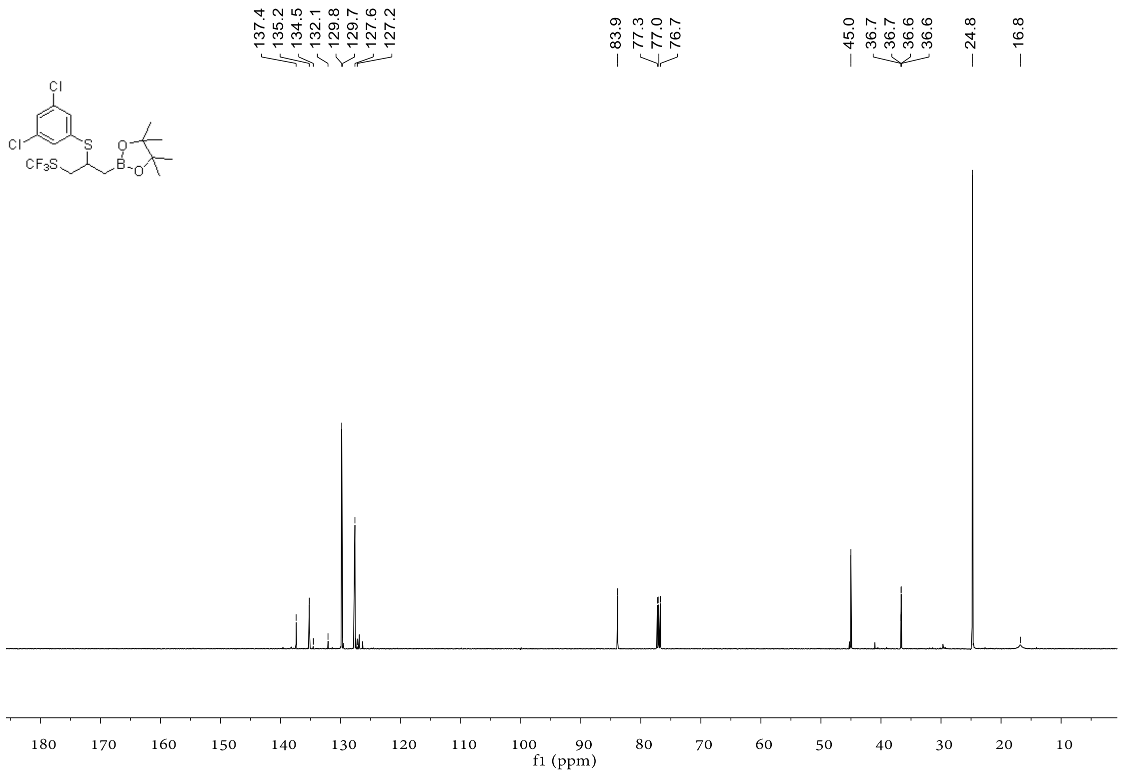

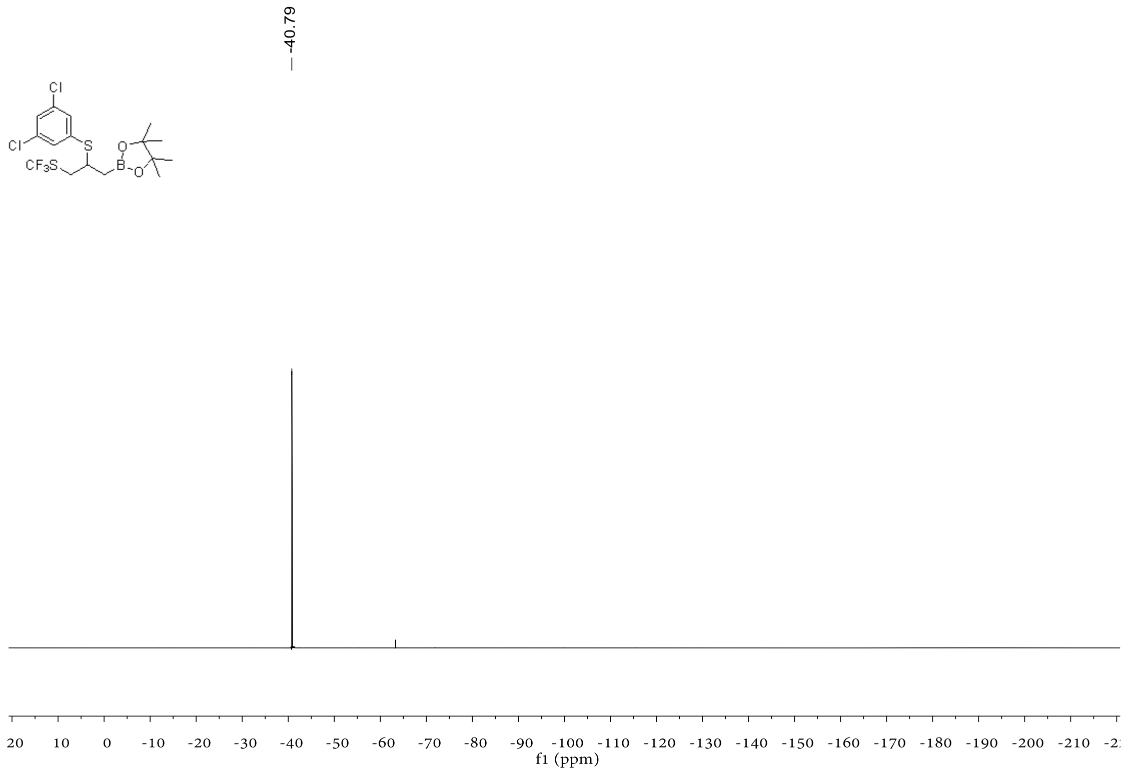

2-(2-((2-Fluorophenyl)thio)-3-((trifluoromethyl)thio)propyl)-4,4,5,5-tetramethyl-1,3,2-dioxaborolane (4g)

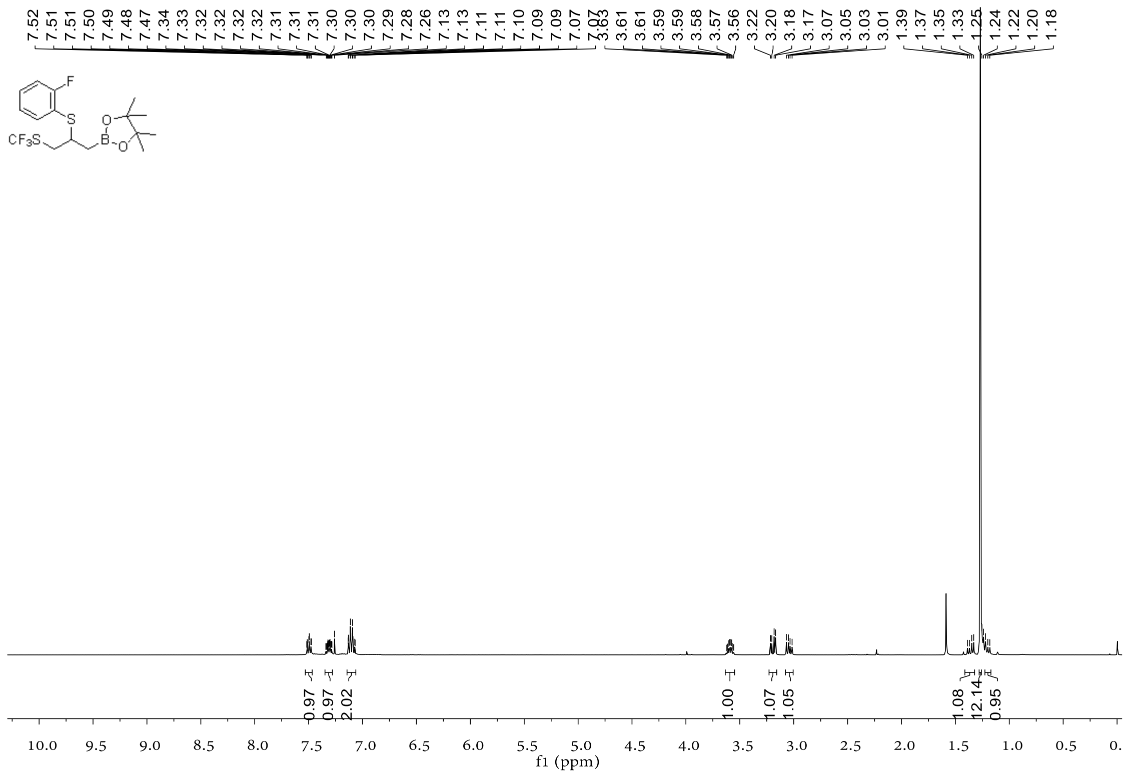

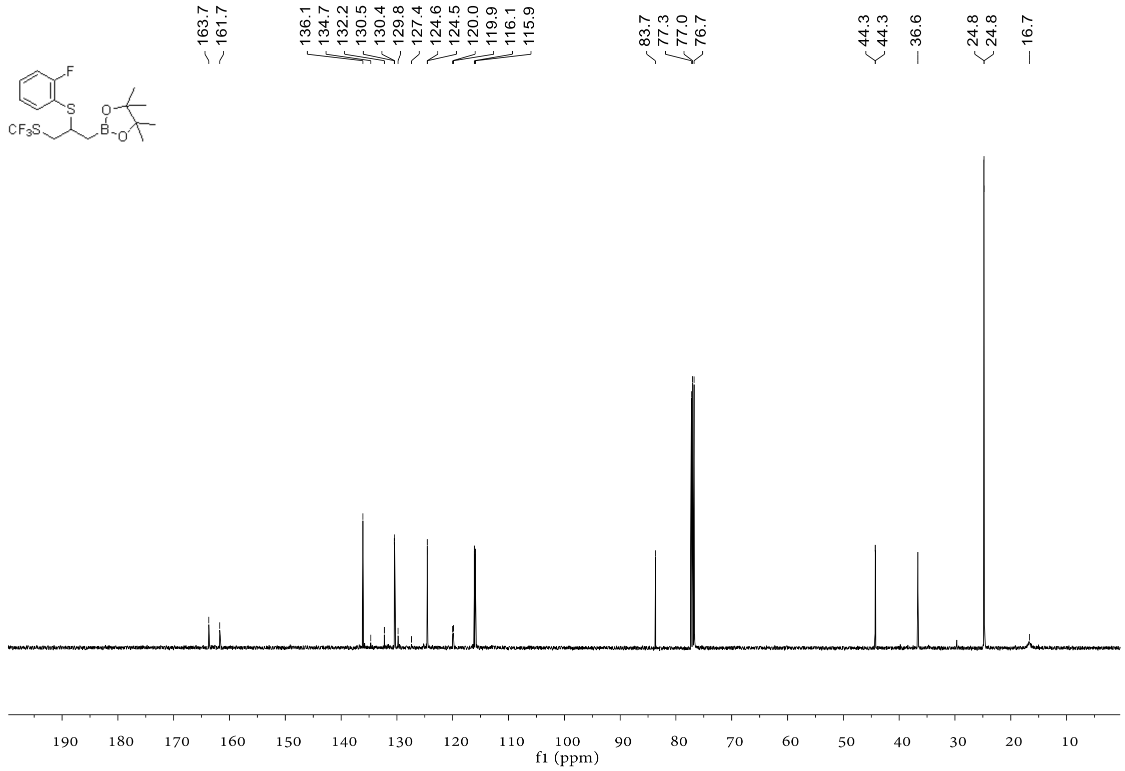
163.7
161.7
136.1
134.7
132.2
130.5
130.4
129.8
127.4
124.6
124.5
120.0
119.9
116.1
115.9
83.7
77.3
77.0
76.7
44.3
44.3
36.6
24.8
24.8
16.7
190 180 170 160 150 140 130 120 110 100 90 80 70 60 50 40 30 20 10
f1 (ppm)

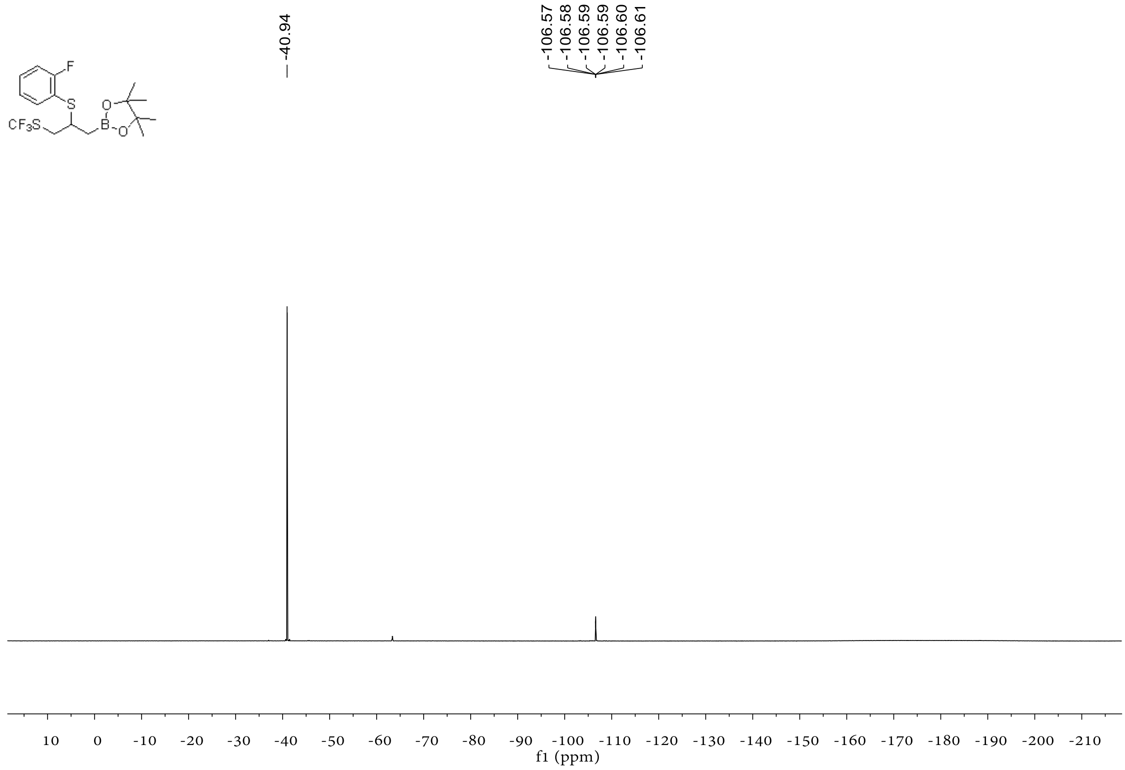
-40.94
-106.57
-106.58
-106.59
-106.59
-106.60
-106.61
10 0 -10 -20 -30 -40 -50 -60 -70 -80 -90 -100 -110 -120 -130 -140 -150 -160 -170 -180 -190 -200 -210
f1 (ppm)

2-(2-((4-Chlorophenyl)thio)-3-((trifluoromethyl)thio)propyl)-4,4,5,5-tetramethyl-1,3,2-dioxaborolane(4g)

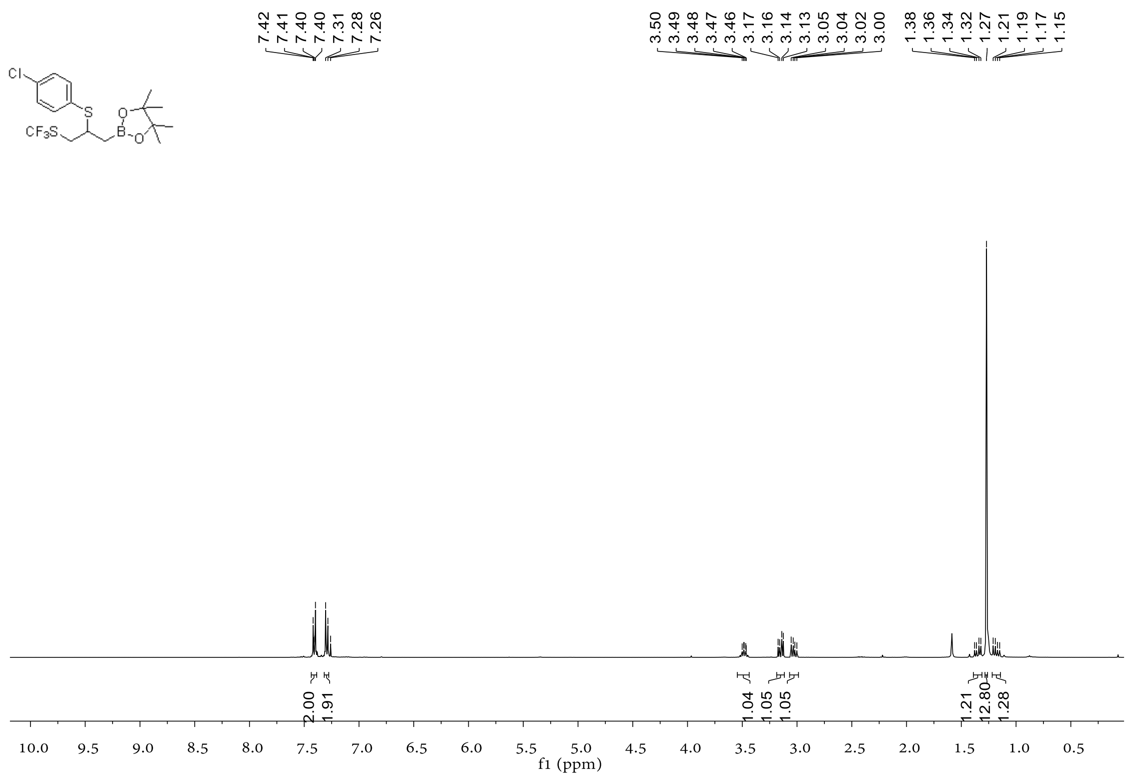

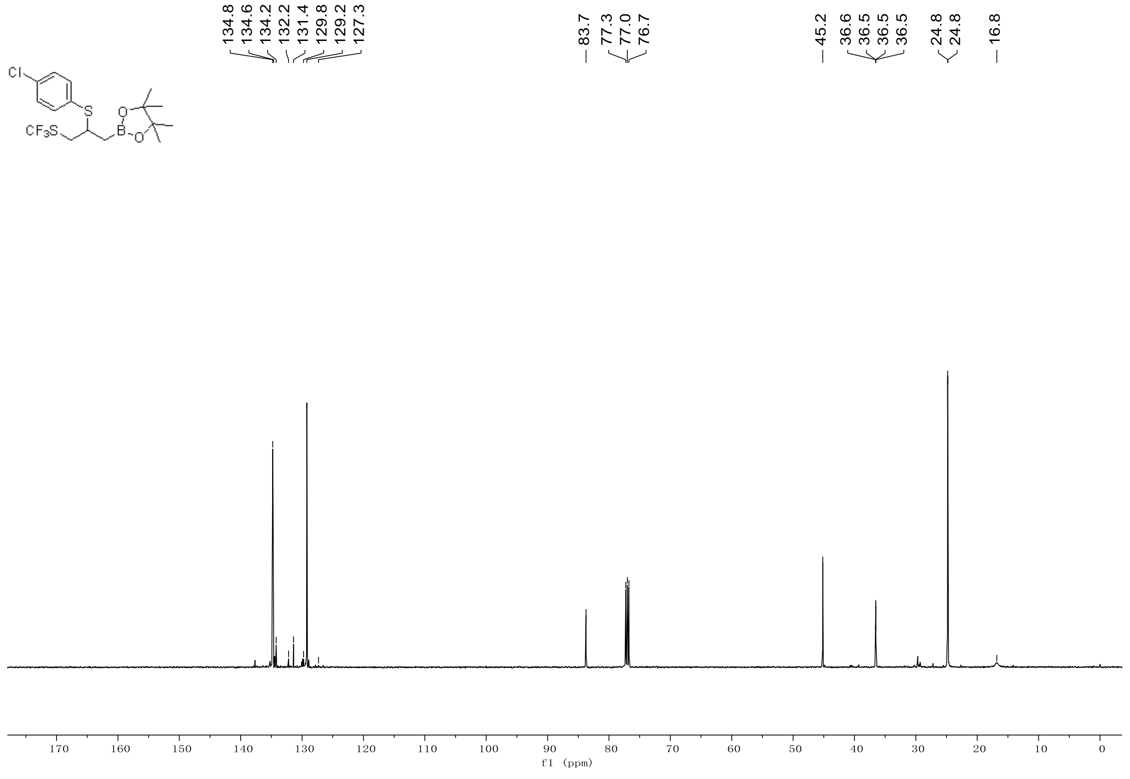

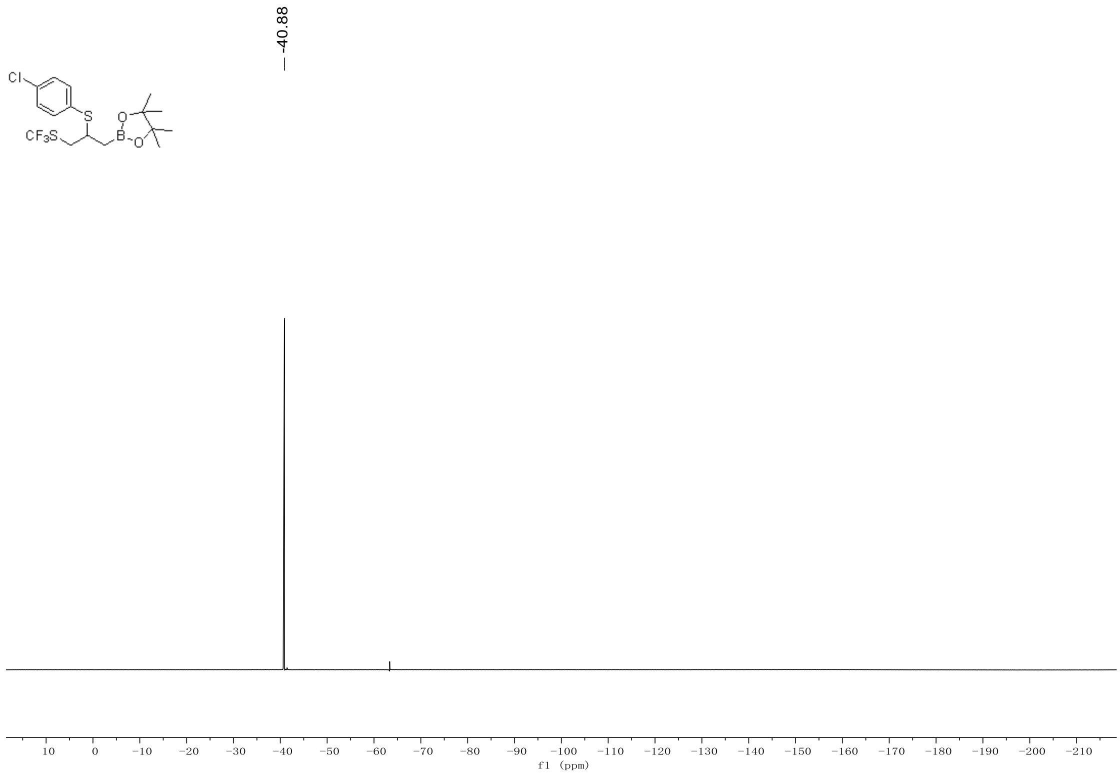

4,4,5,5-Tetramethyl-2-(3-(phenylthio)-4-((trifluoromethyl)thio)butyl)-1,3,2-dioxaborolane(4h)

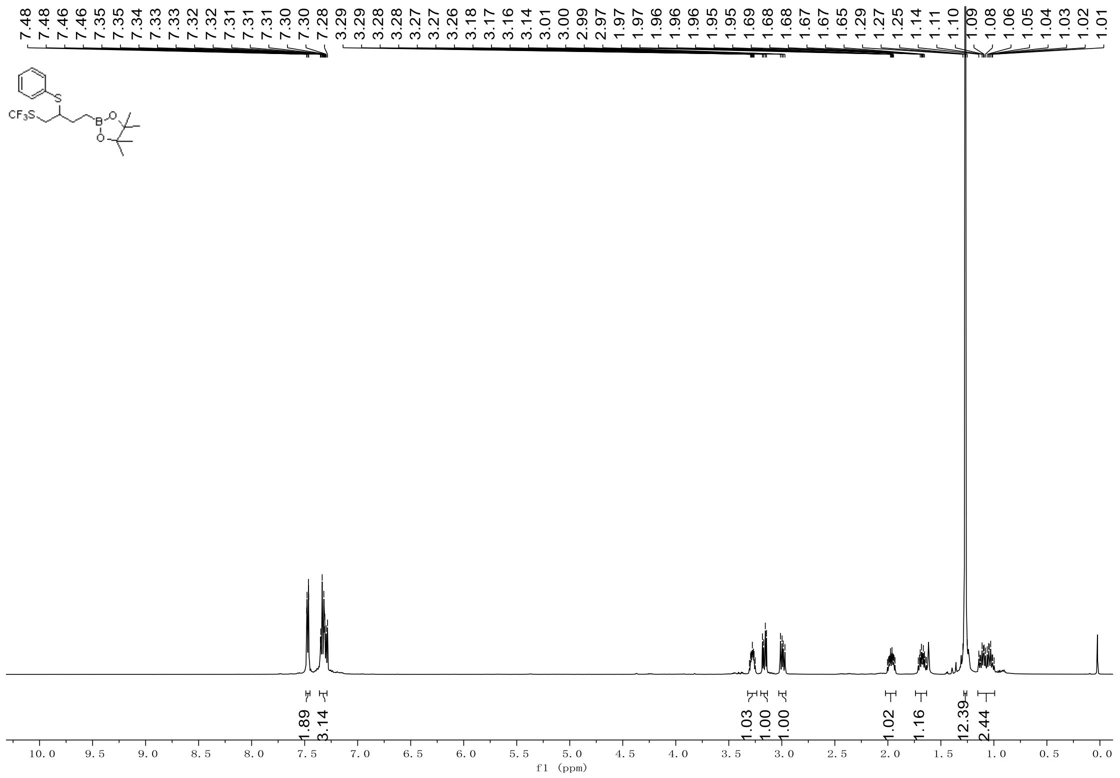

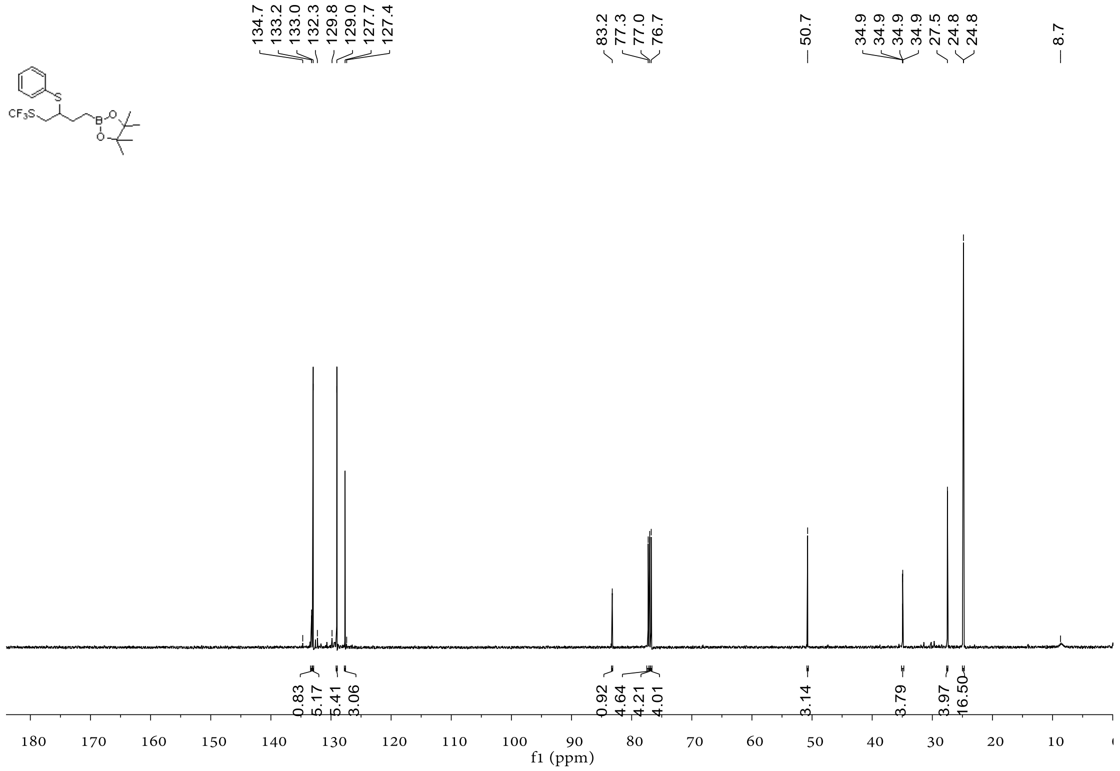

134.7
133.2
133.0
132.3
129.8
129.0
127.7
127.4
83.2
77.3
77.0
76.7
50.7
34.9
34.9
34.9
34.9
27.5
24.8
24.8
8.7
CF$_3$S
S
B
O
O
0.83
5.17
5.41
3.06
0.92
4.64
4.21
4.01
3.14
3.79
3.97
16.50
180 170 160 150 140 130 120 110 100 90 80 70 60 50 40 30 20 10
f1 (ppm)

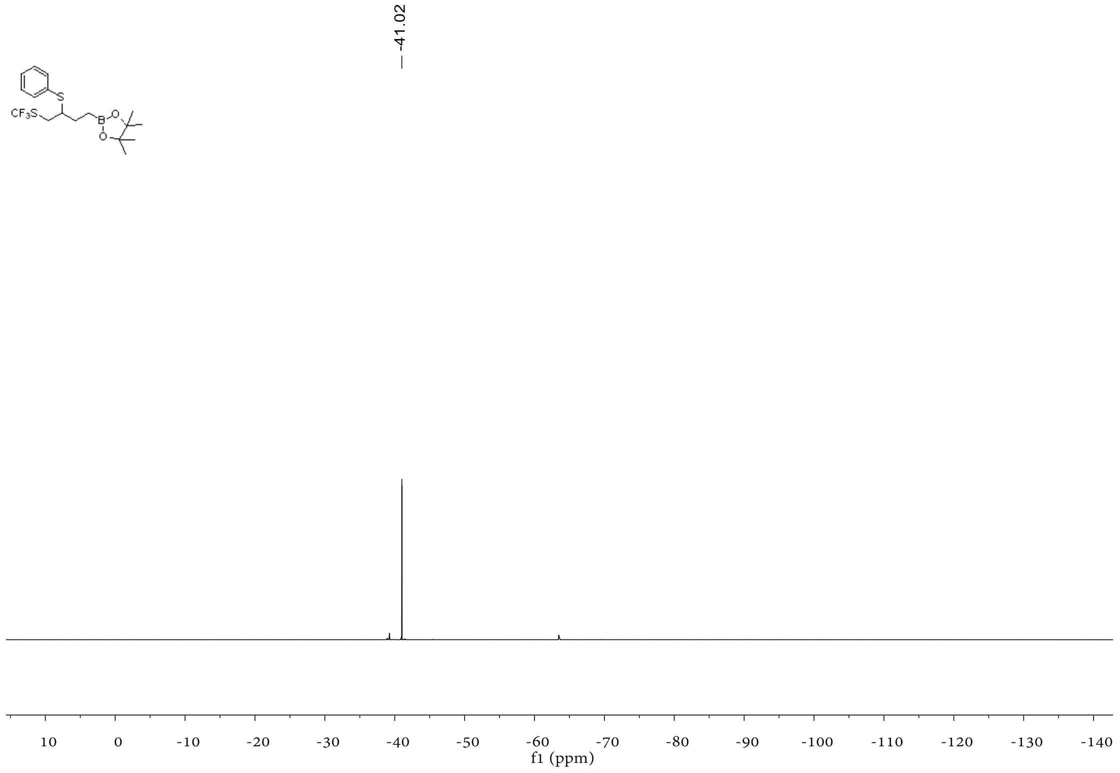

-41.02
CF$_3$S
S
B
O
O
10 0 -10 -20 -30 -40 -50 -60 -70 -80 -90 -100 -110 -120 -130 -140
f1 (ppm)

4-(Phenylthio)-5-((trifluoromethyl)thio)pentyl 4-chlorobenzoate (5a)

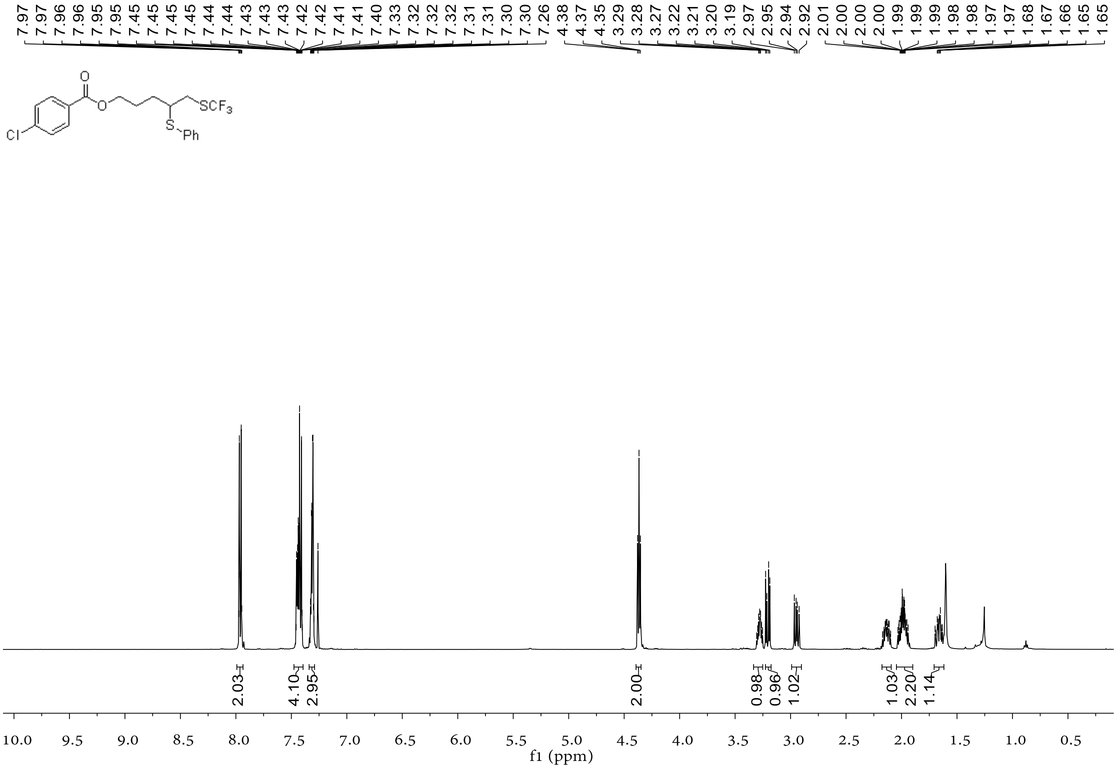

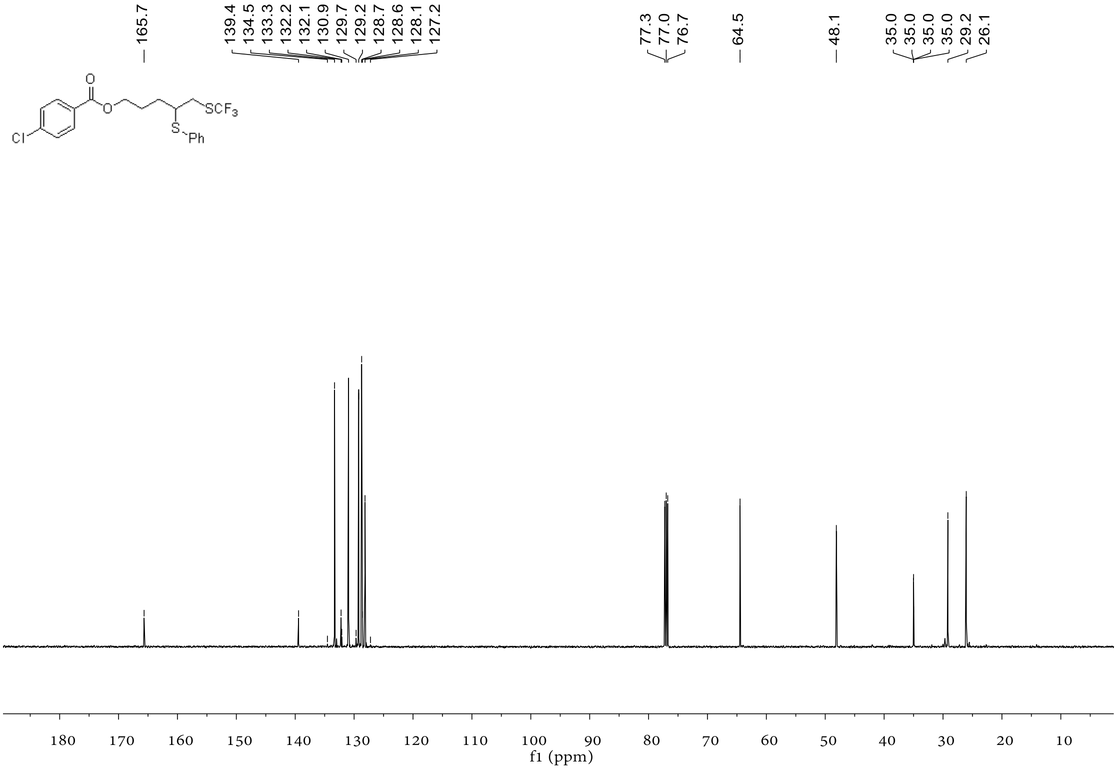

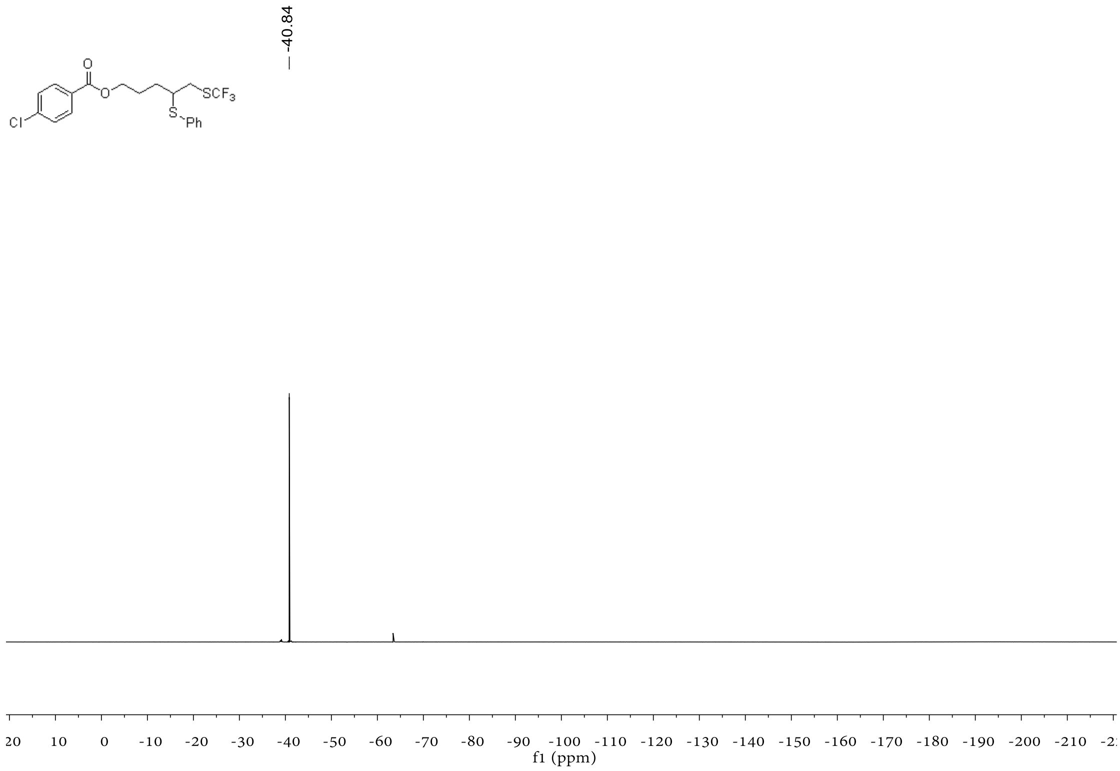

5-(Phenylthio)-6-((trifluoromethyl)thio)hexyl 4-methylbenzoate (5b)

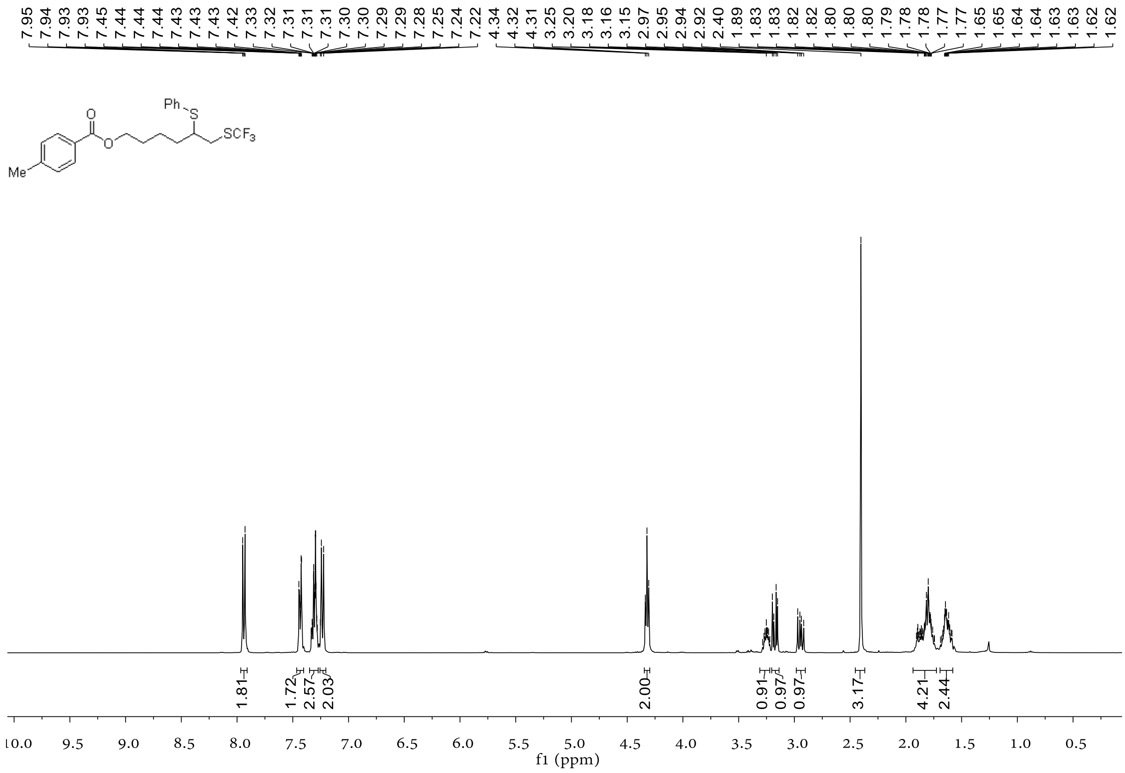

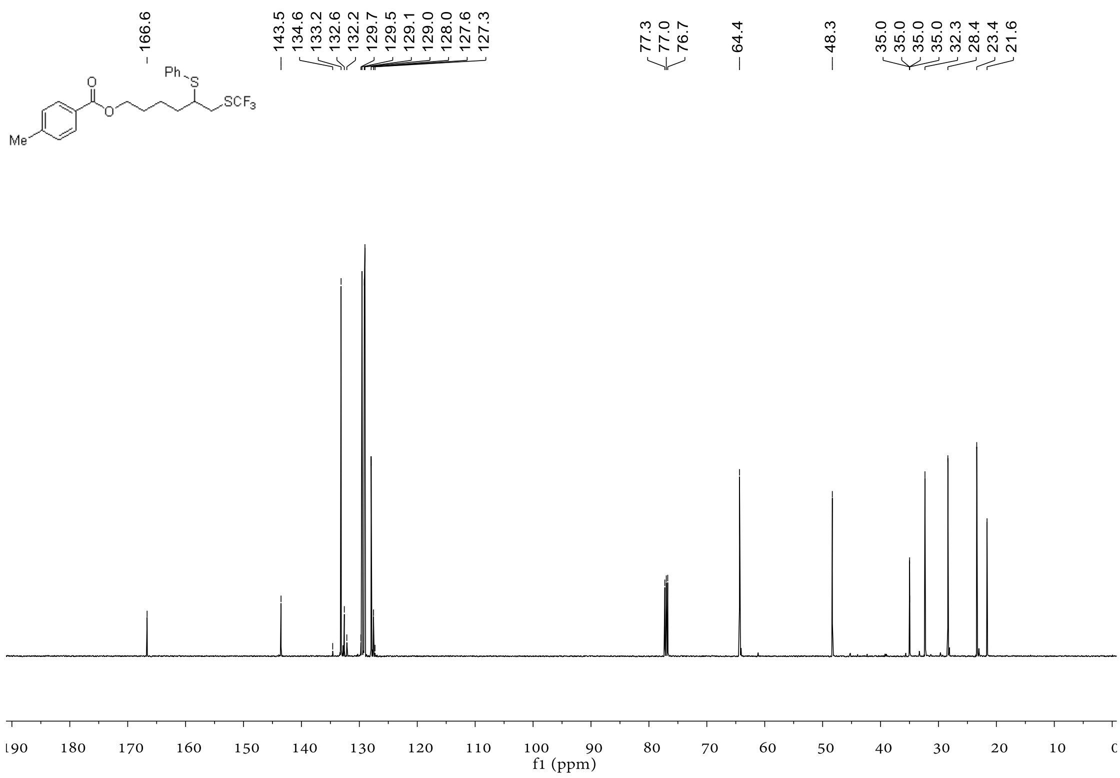
166.6
143.5
134.6
133.2
132.6
132.2
129.7
129.5
129.1
129.0
128.0
127.6
127.3
77.3
77.0
76.7
64.4
48.3
35.0
35.0
35.0
35.0
32.3
28.4
23.4
21.6
Ph
S
O
O
SCF3
Me
f1 (ppm)

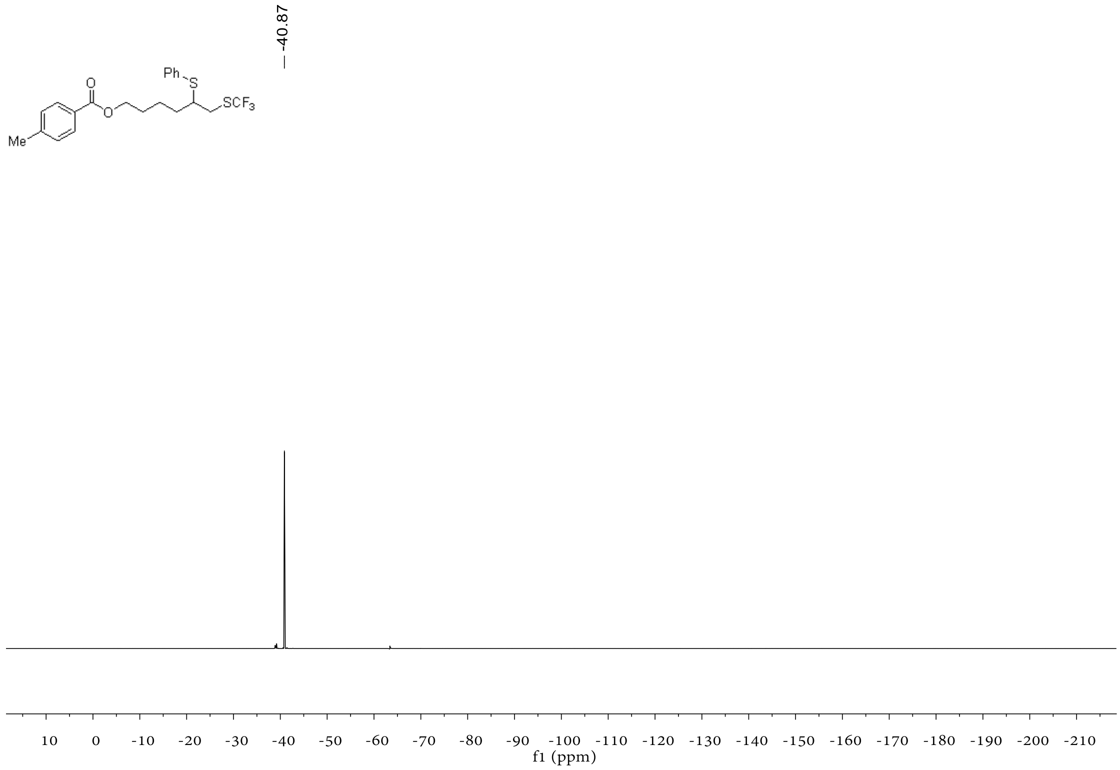
-40.87
Ph
S
O
O
SCF3
Me
f1 (ppm)

4-(Phenylthio)-5-((trifluoromethyl)thio)pentyl 3,5-dimethylbenzoate (5c)

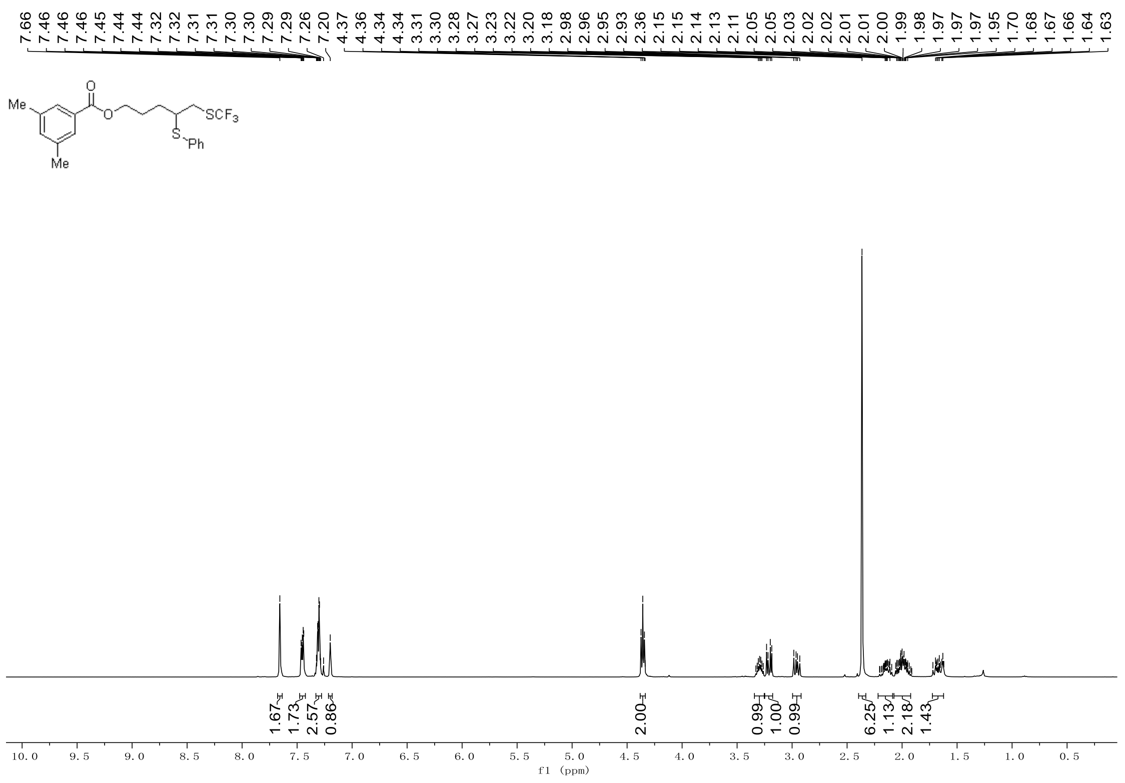

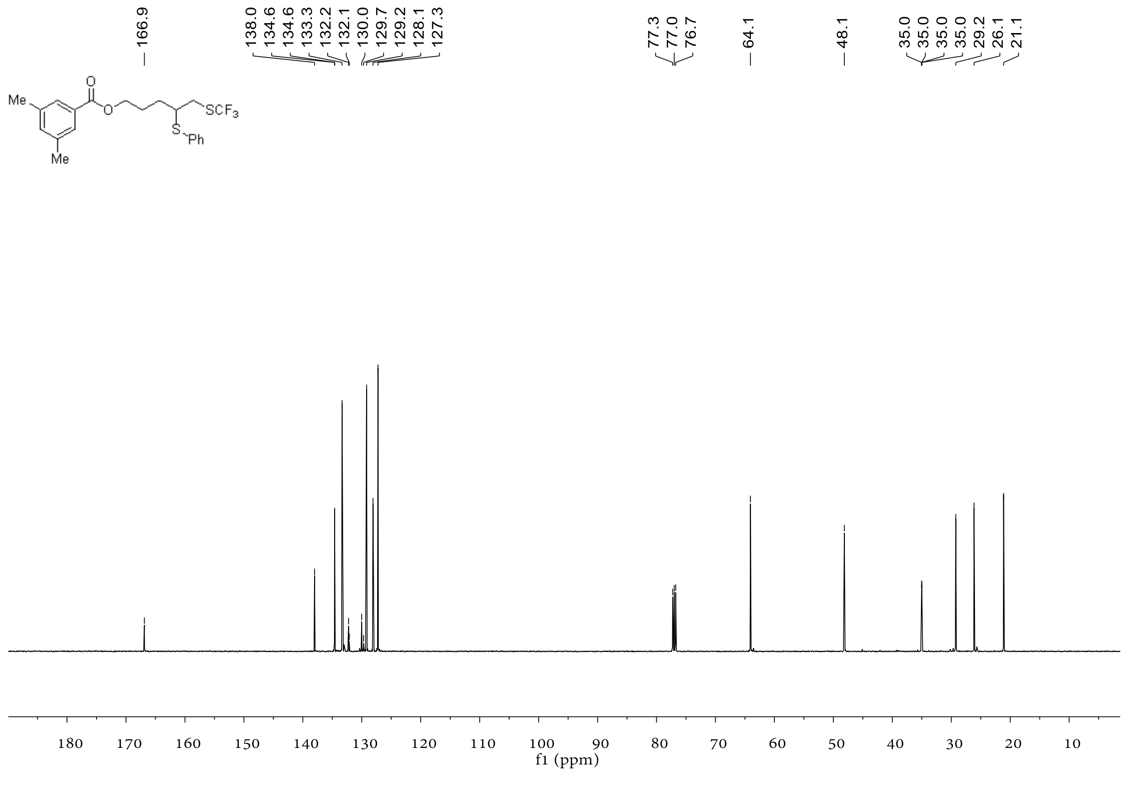

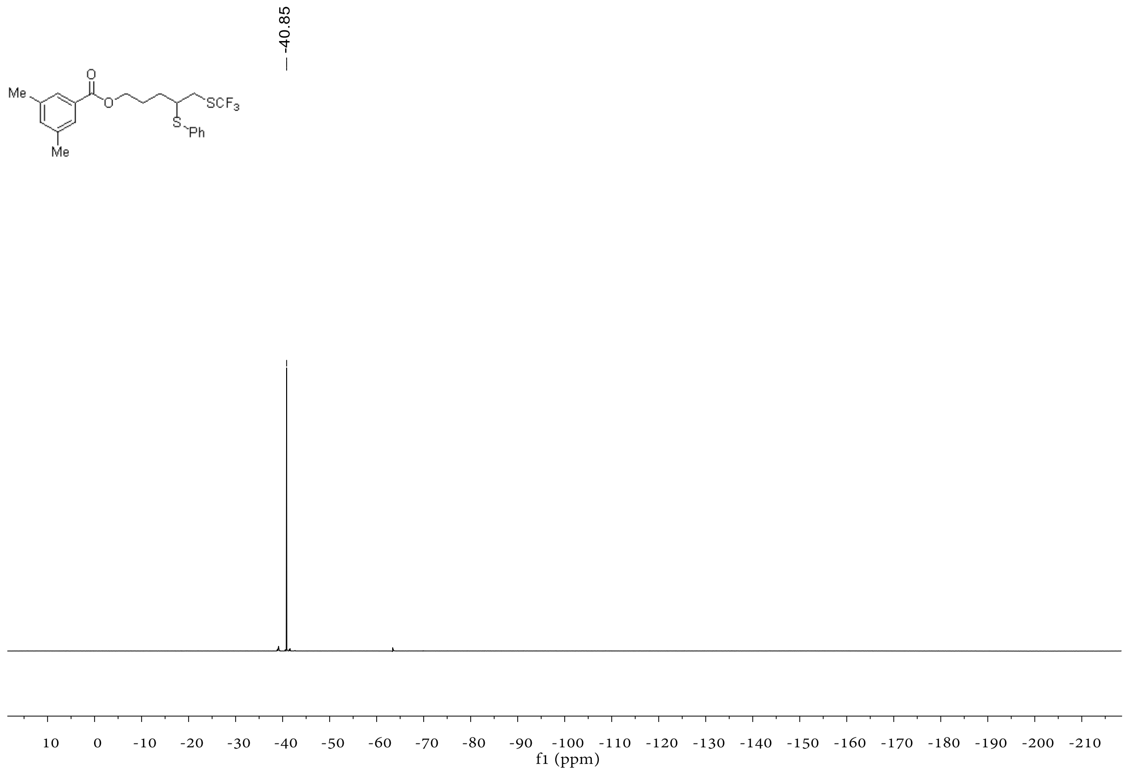

5-(Phenylthio)-6-((trifluoromethyl)thio)hexyl 2-naphthoate (5d)

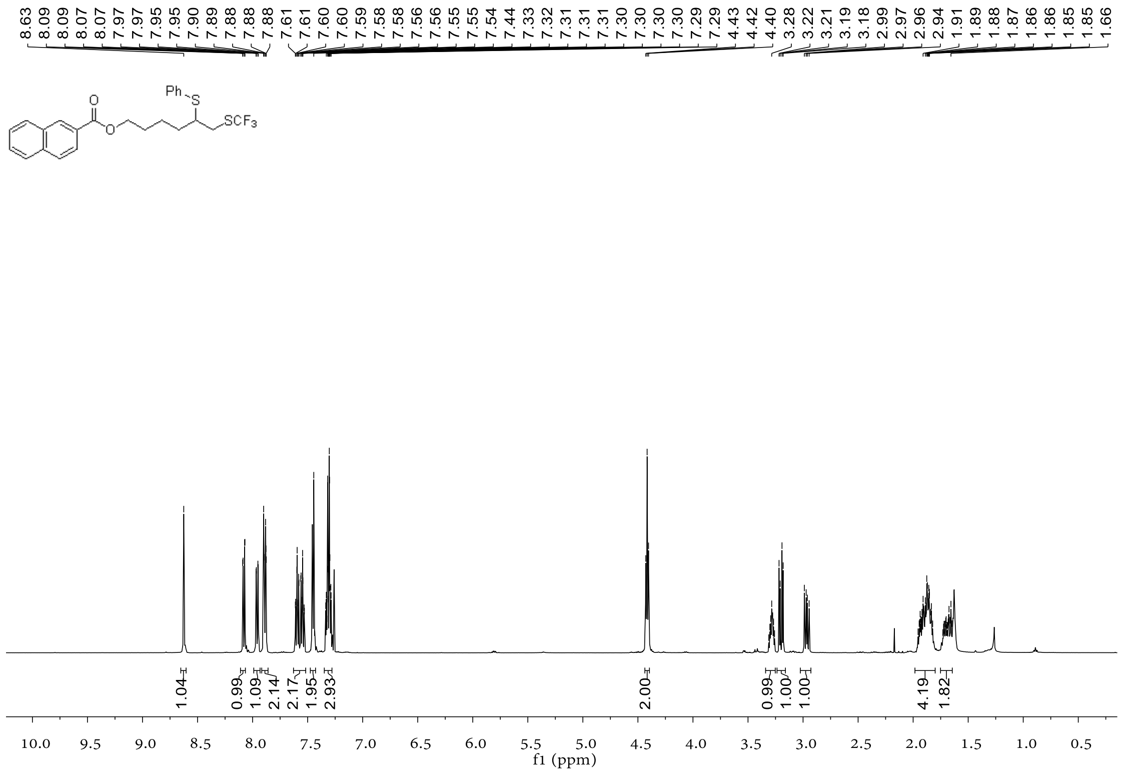

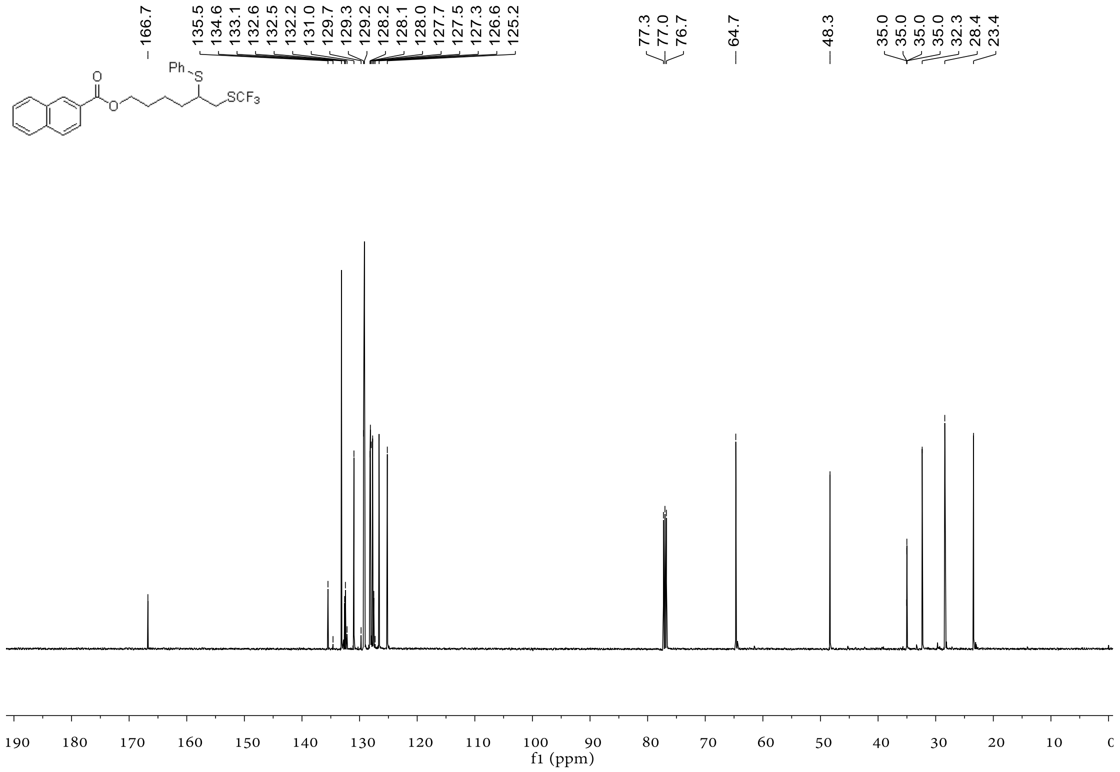
166.7
135.5
134.6
133.1
132.6
132.5
132.2
131.0
129.7
129.3
129.2
128.2
128.1
128.0
127.7
127.5
127.3
126.6
125.2
77.3
77.0
76.7
64.7
48.3
35.0
35.0
35.0
35.0
32.3
28.4
23.4
Ph
S
SCF3
O
O
f1 (ppm)

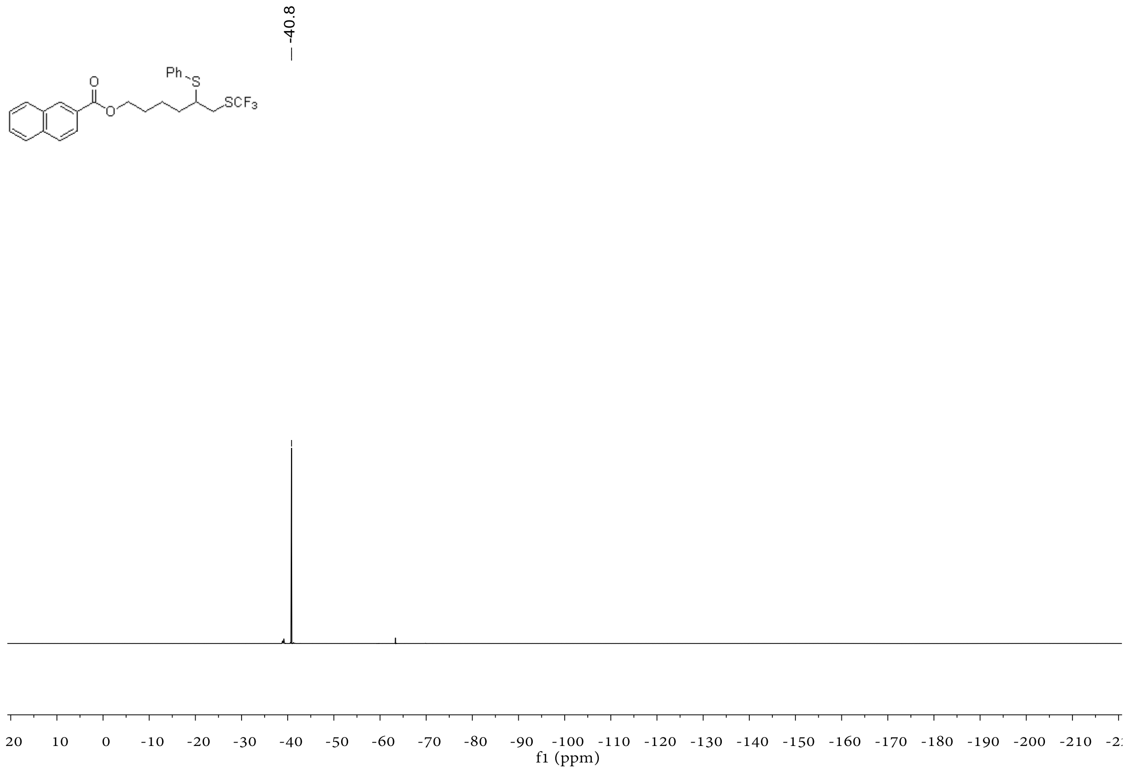
-40.8
Ph
S
SCF3
O
O
f1 (ppm)

(5-(3-Bromophenoxy)-1-((trifluoromethyl)thio)pentan-2-yl)(phenyl)sulfane (5e)

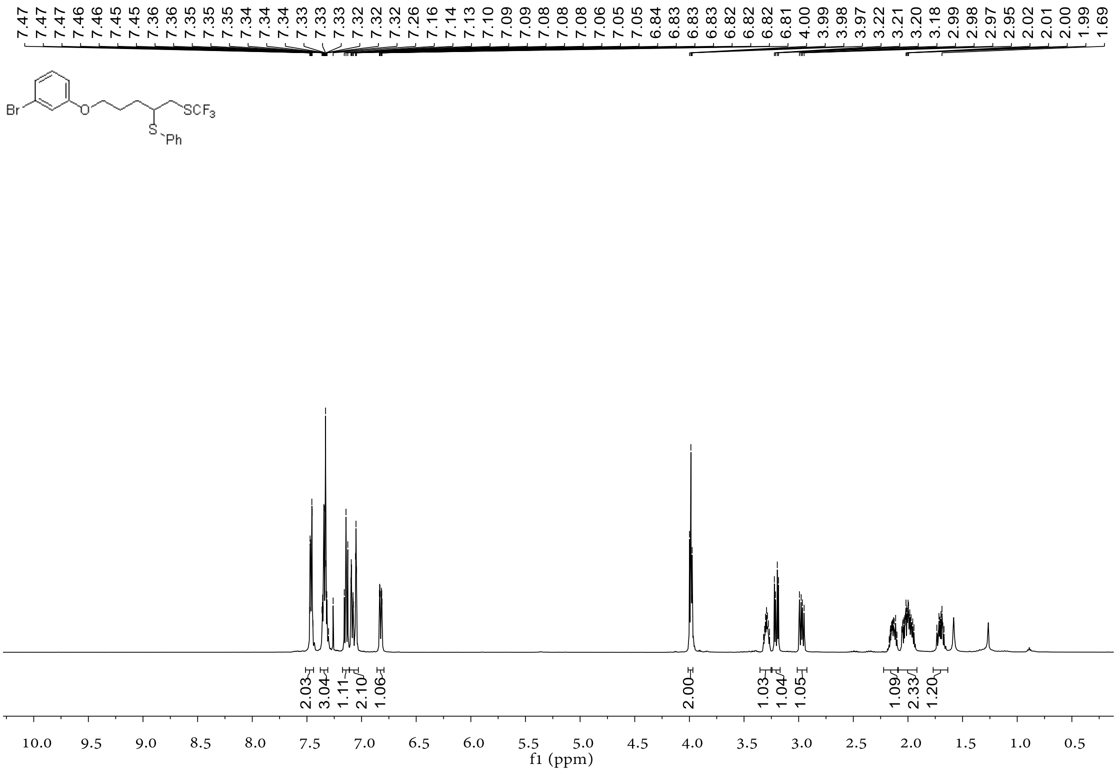

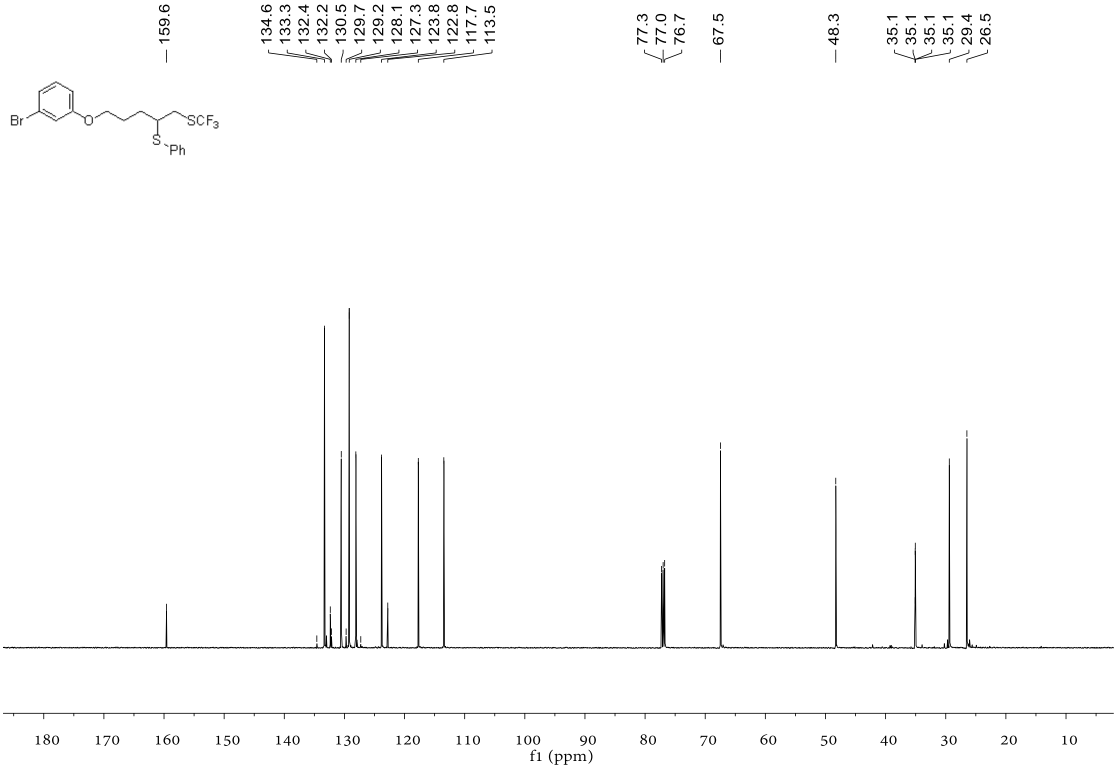

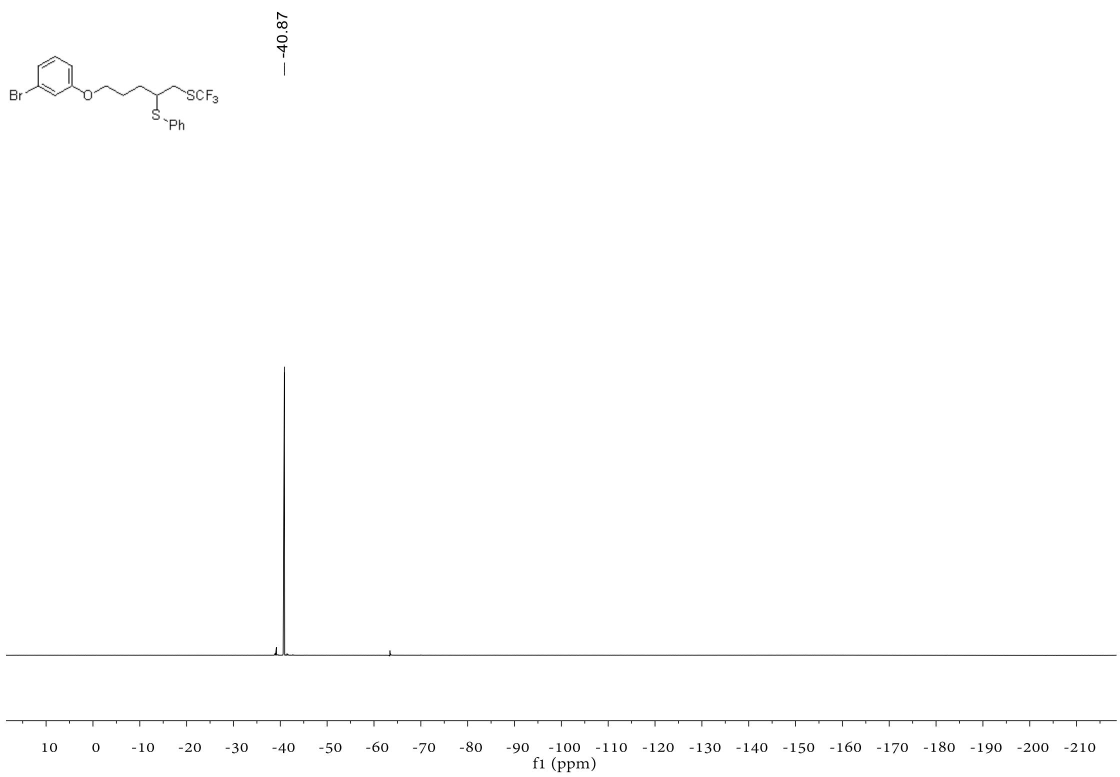

2-Phenyl-3-((4-(phenylthio)-5-((trifluoromethyl)thio)pentyl)oxy)-4*H*-chromen-4-one (5f)

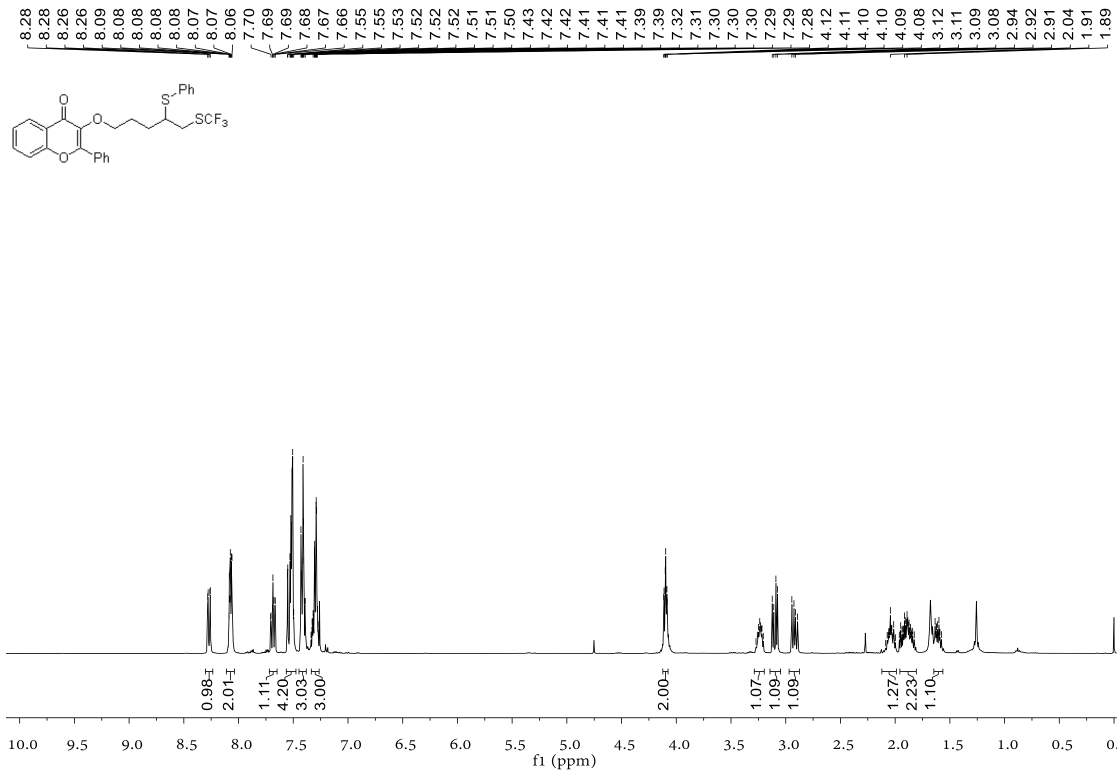

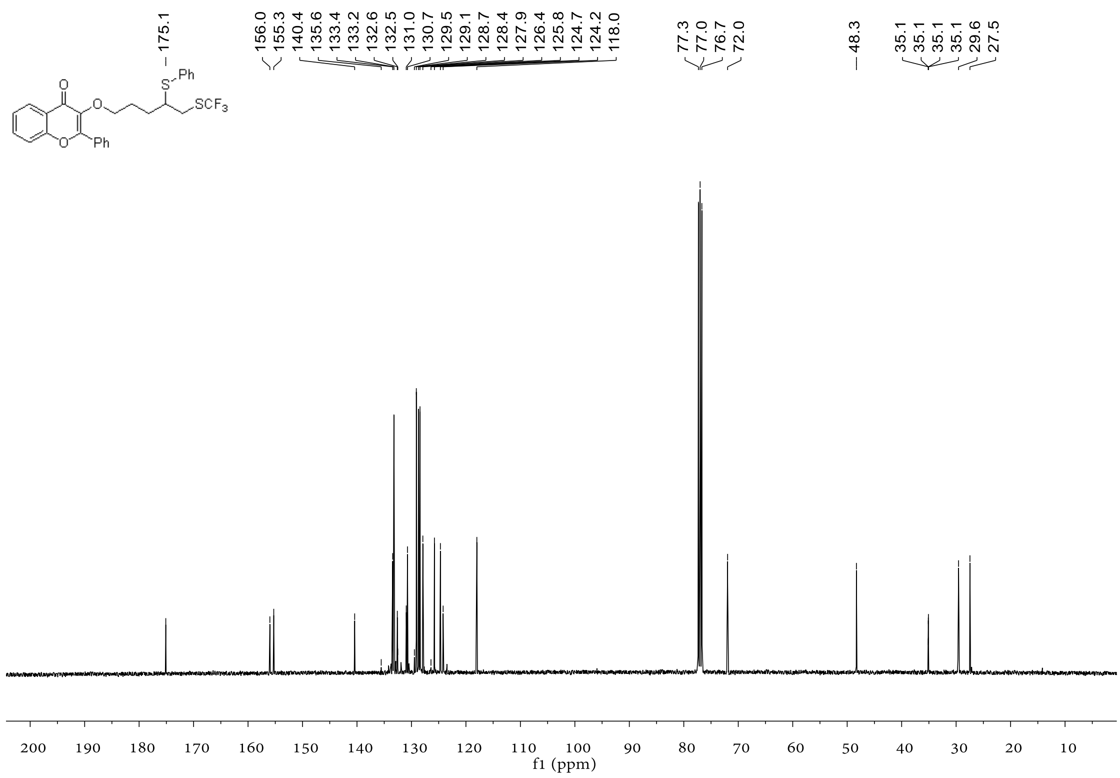

175.1
156.0
155.3
140.4
135.6
133.4
133.2
132.6
132.5
131.0
130.7
129.5
129.1
128.7
128.4
127.9
126.4
125.8
124.7
124.2
118.0
77.3
77.0
76.7
72.0
48.3
35.1
35.1
35.1
35.1
29.6
27.5
f1 (ppm)

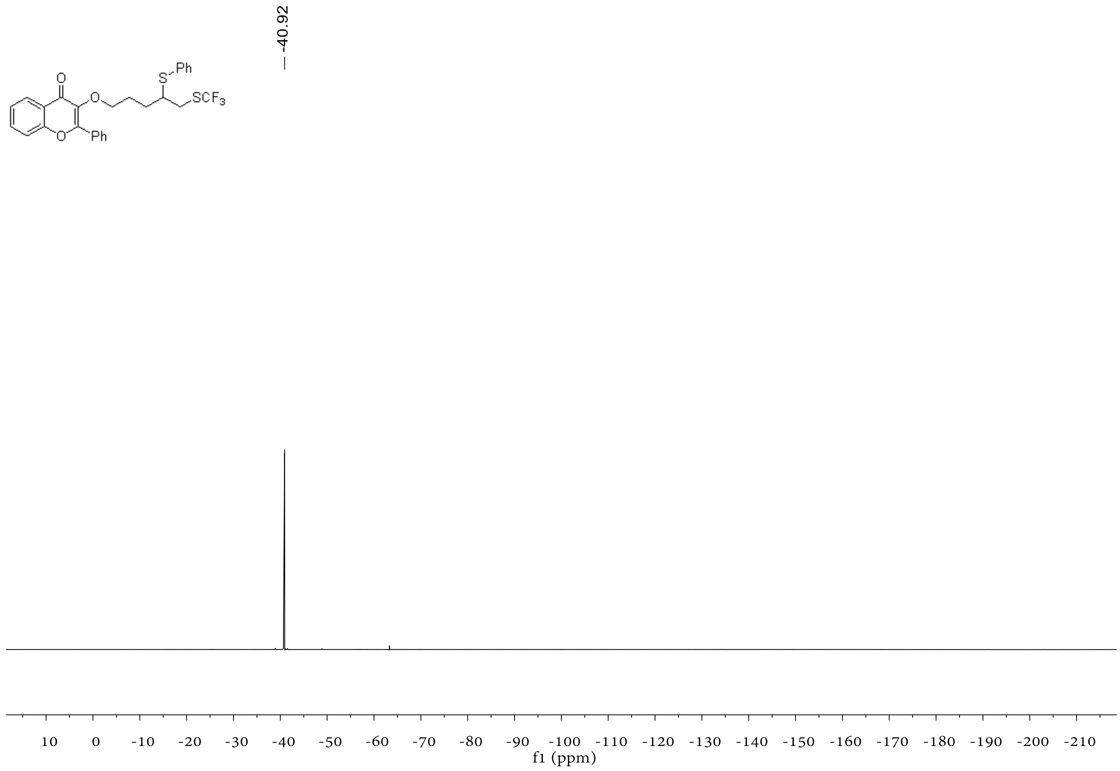

-40.92
f1 (ppm)

(4-(3-Methoxyphenoxy)-1-((trifluoromethyl)thio)butan-2-yl)(phenyl)sulfane (5g)

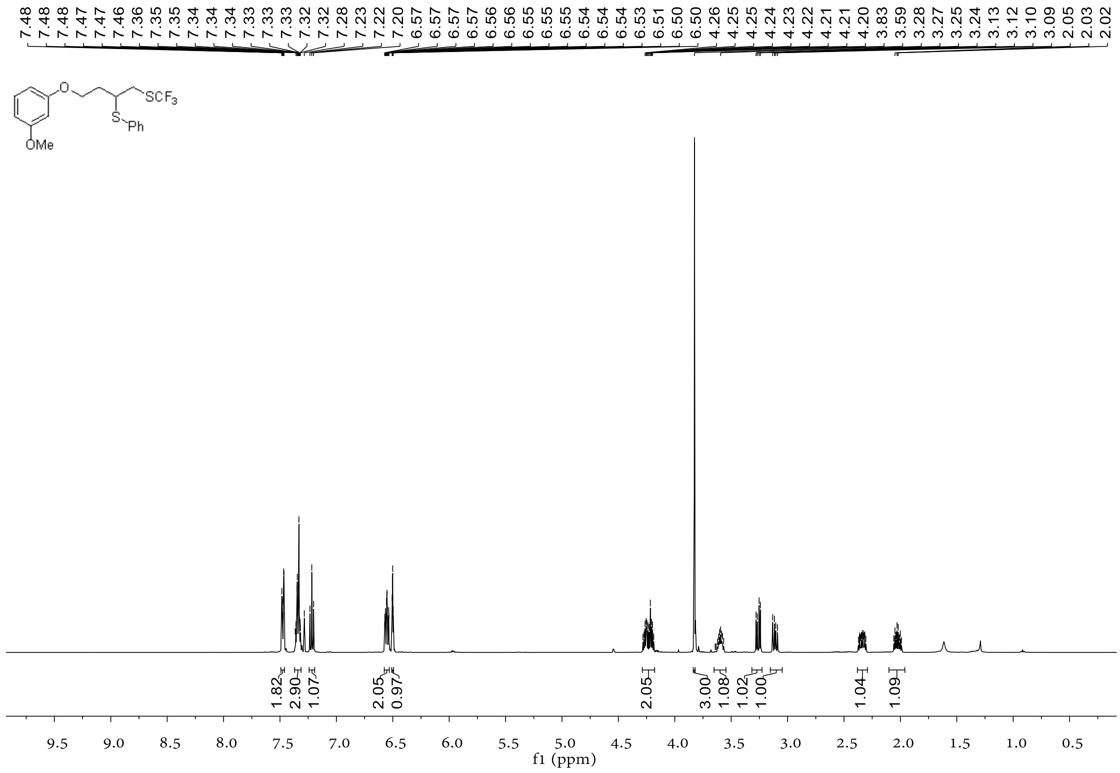

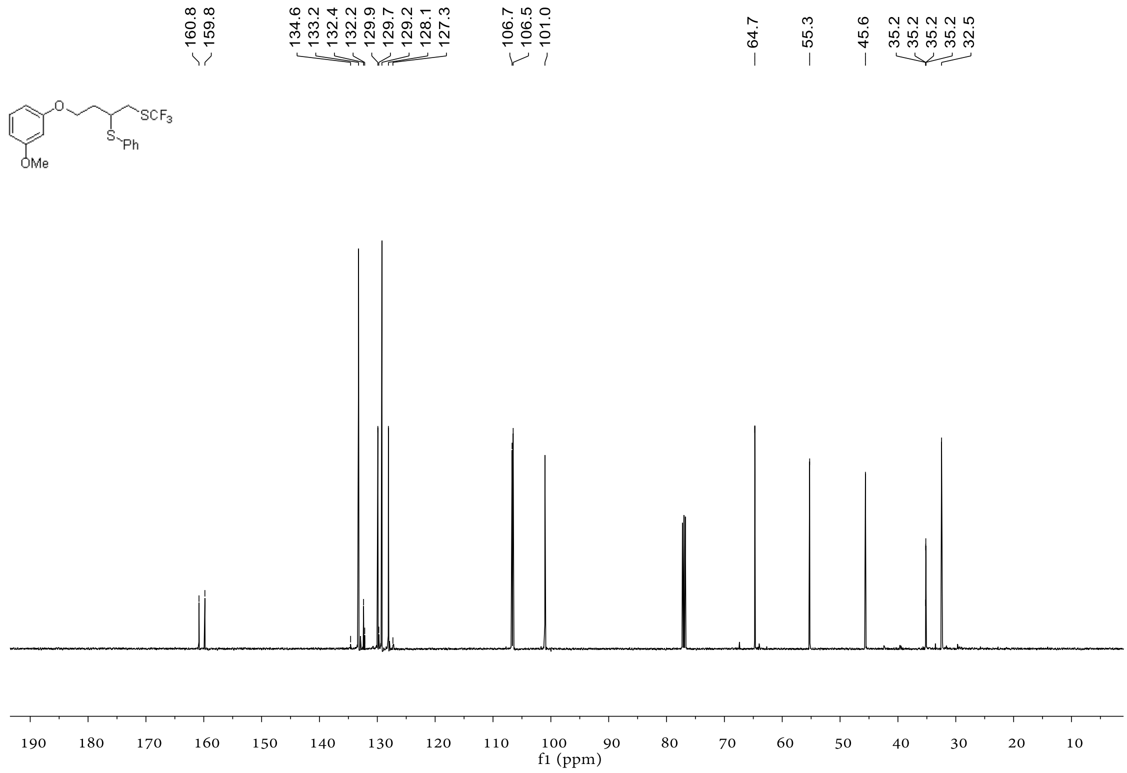

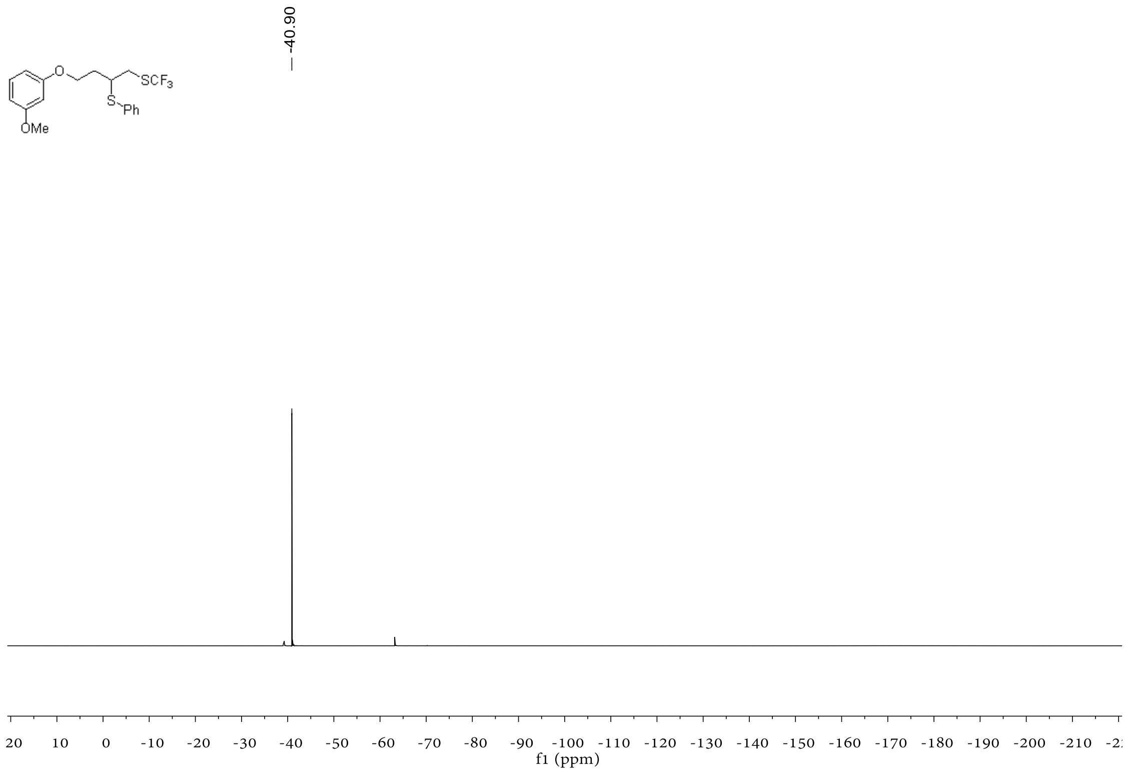

Phenyl(4-phenyl-1-((trifluoromethyl)thio)butan-2-yl)sulfane (5h)

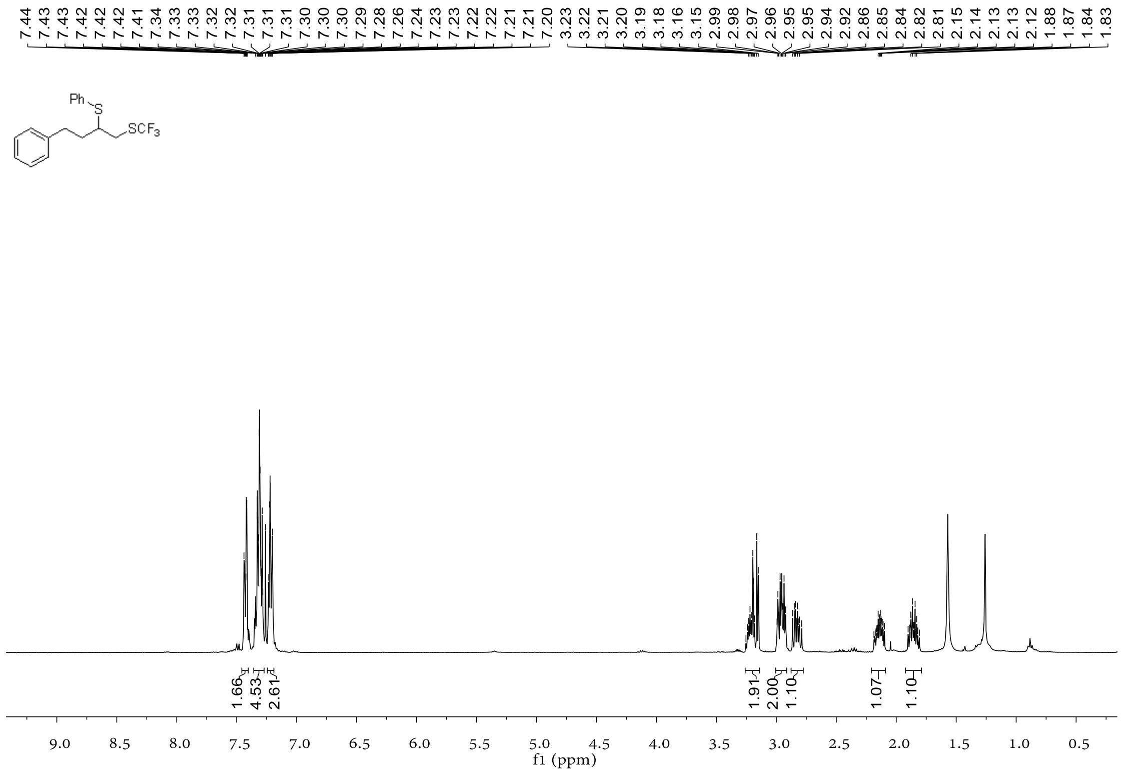

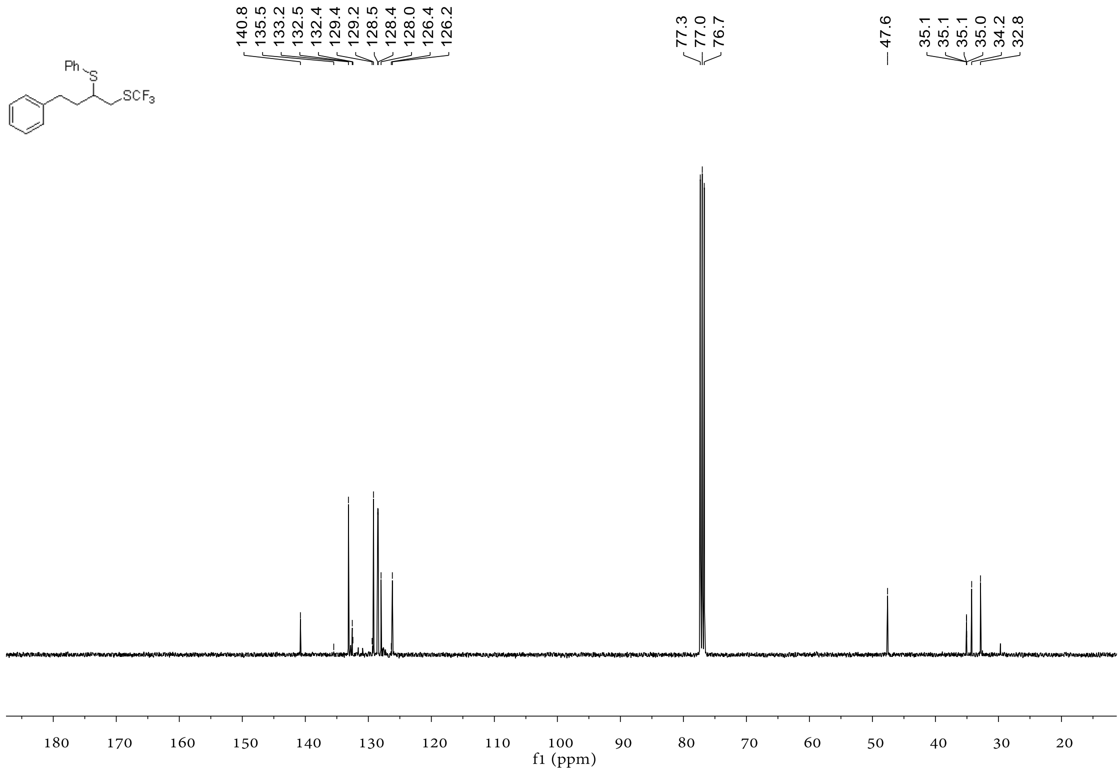
Ph
S
SCF3
140.8
135.5
133.2
132.5
132.4
129.4
129.2
128.5
128.4
128.0
126.4
126.2
77.3
77.0
76.7
47.6
35.1
35.1
35.1
35.0
34.2
32.8
180 170 160 150 140 130 120 110 100 90 80 70 60 50 40 30 20
f1 (ppm)

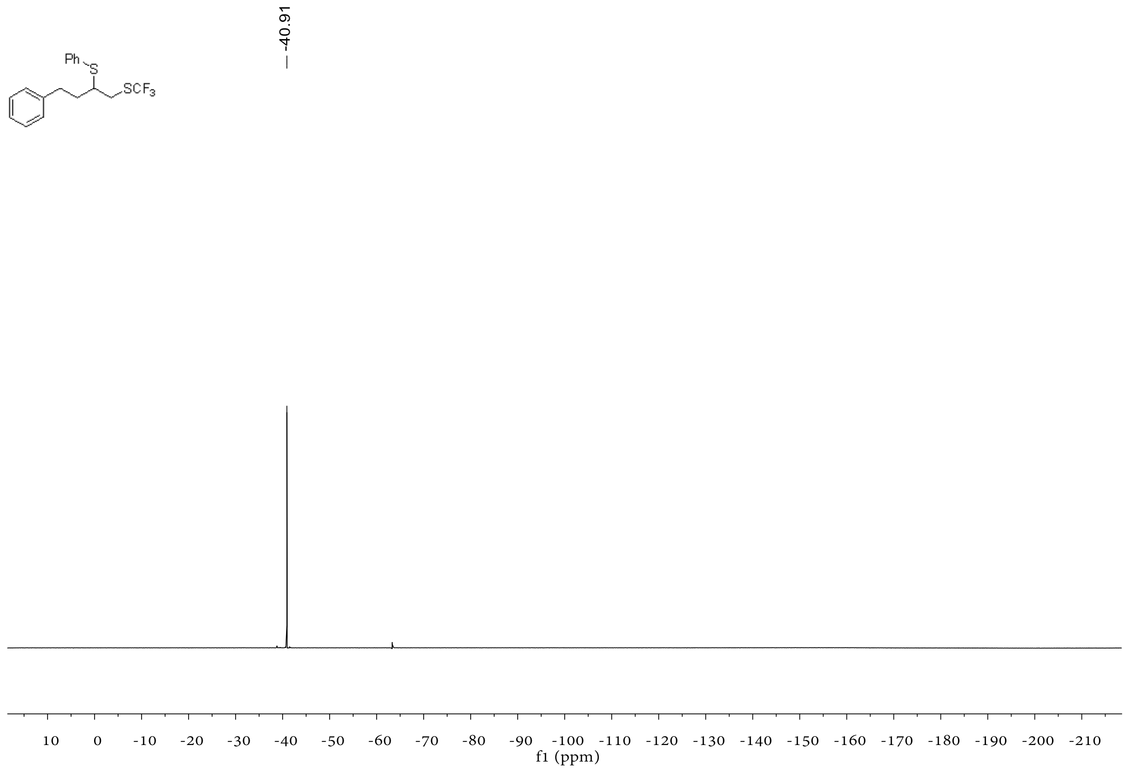
Ph
S
SCF3
-40.91
10 0 -10 -20 -30 -40 -50 -60 -70 -80 -90 -100 -110 -120 -130 -140 -150 -160 -170 -180 -190 -200 -210
f1 (ppm)

(4-Methoxyphenyl)(4-phenyl-1-((trifluoromethyl)thio)butan-2-yl)sulfane (5i)

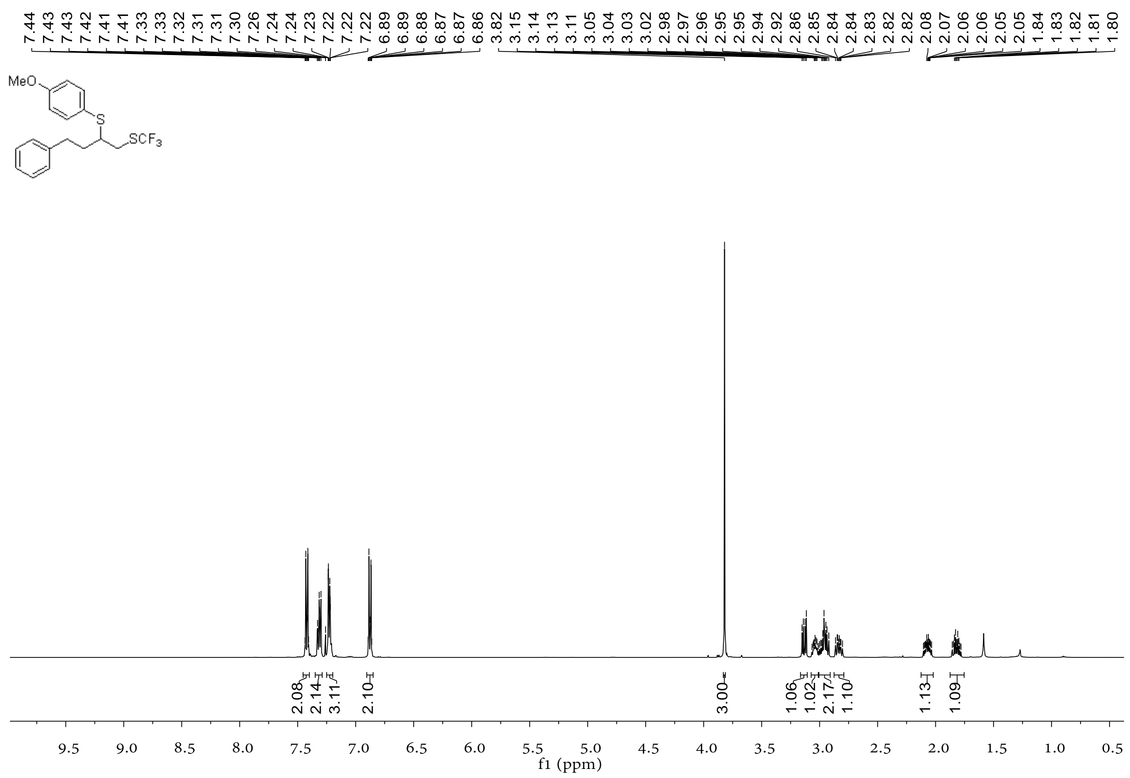

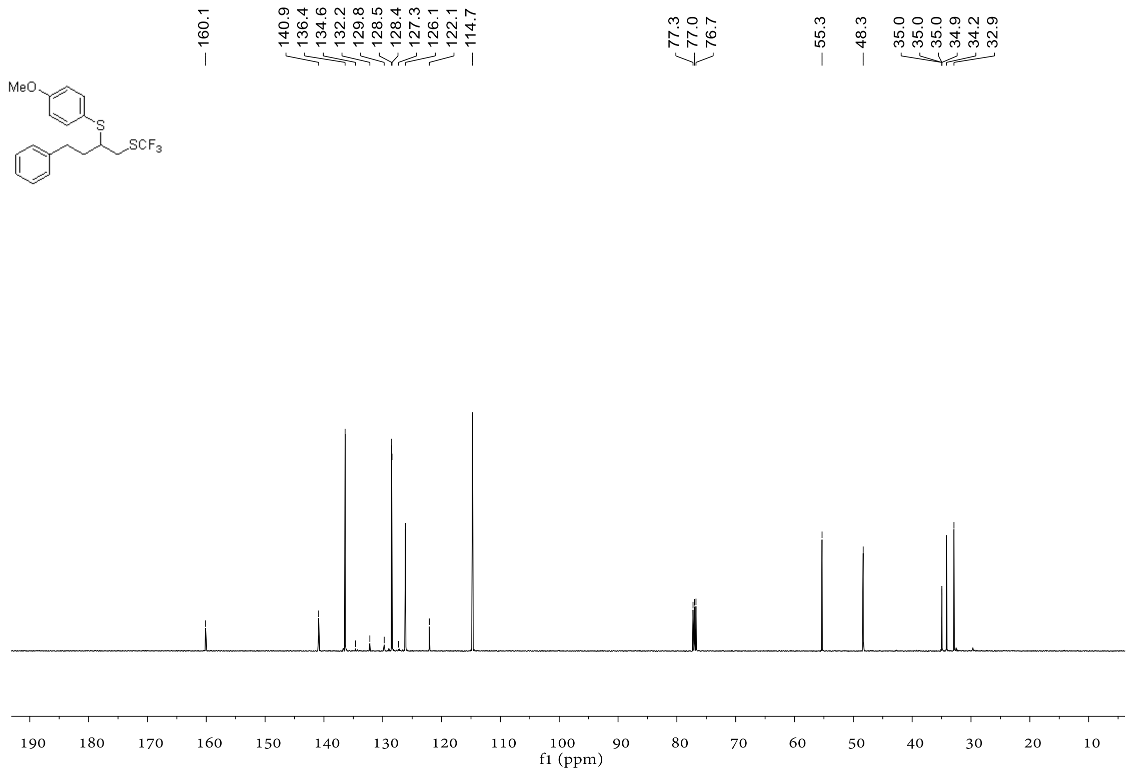

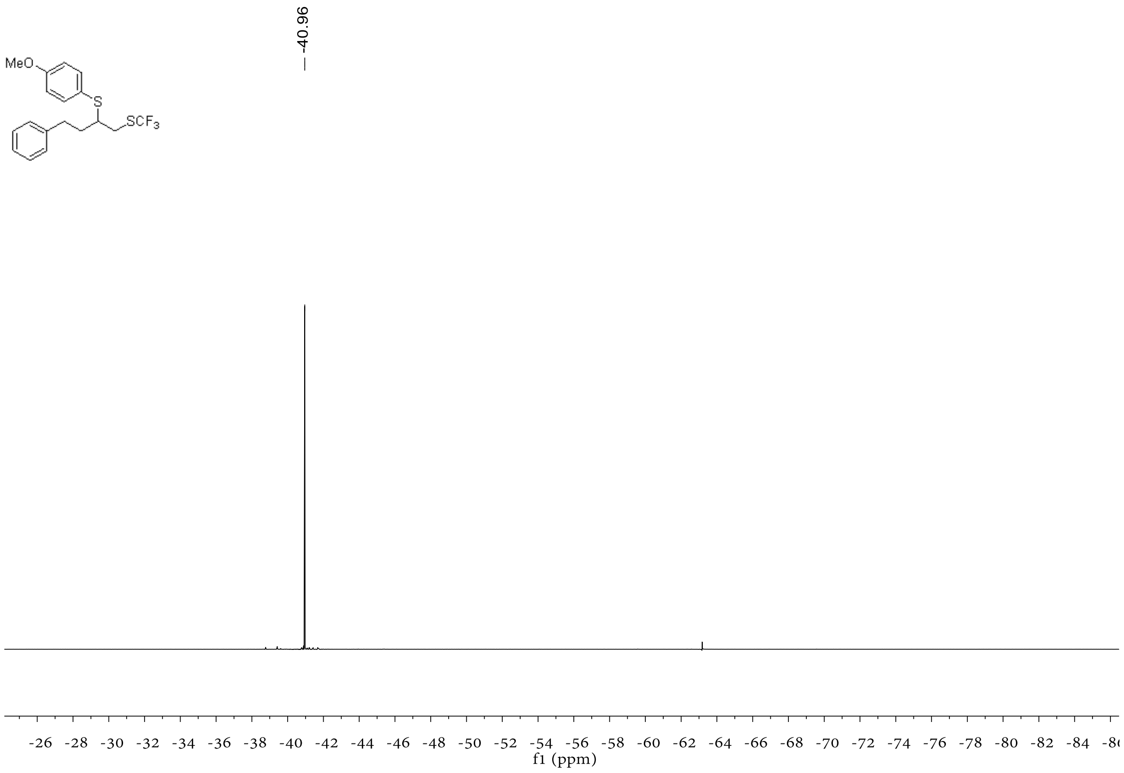

2-(4-(Phenylthio)-5-((trifluoromethyl)thio)pentyl)isoindoline-1,3-dione (5j)

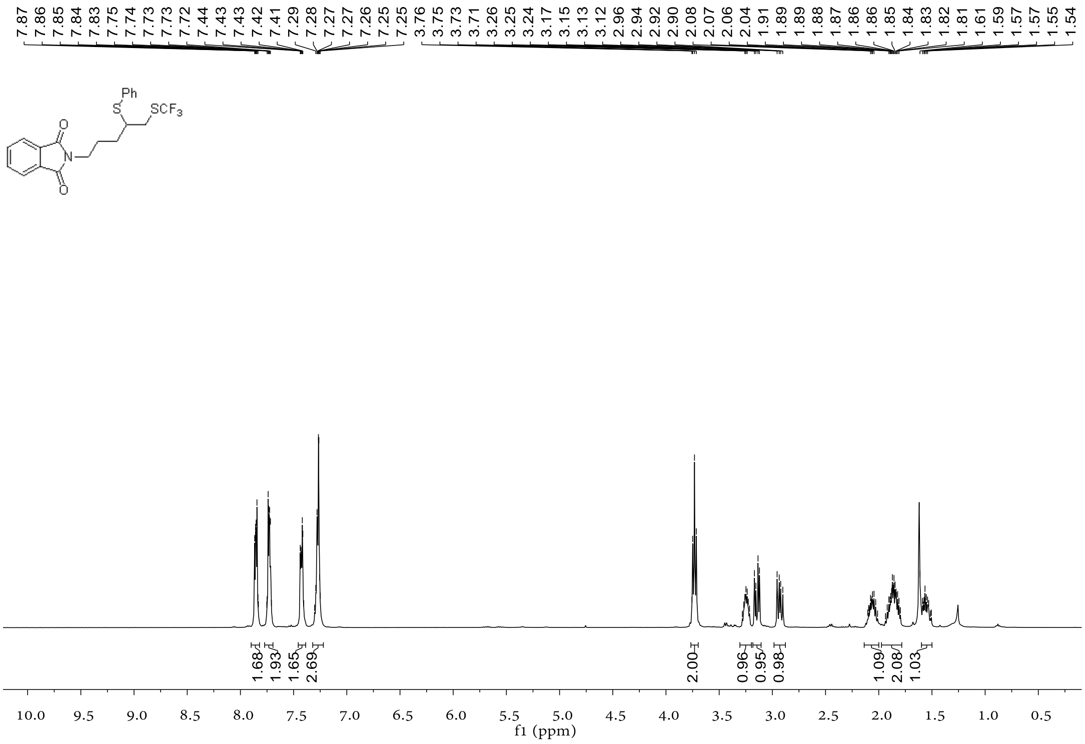

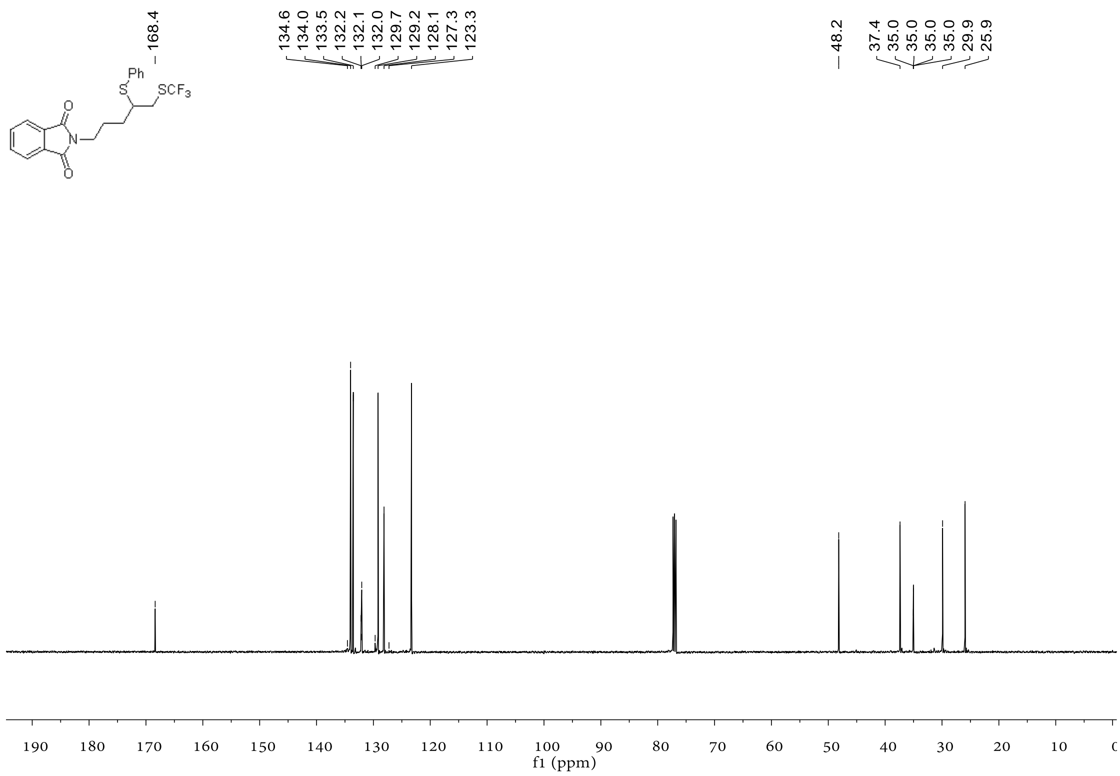
168.4
134.6
134.0
133.5
132.2
132.1
132.0
129.7
129.2
128.1
127.3
123.3
48.2
37.4
35.0
35.0
35.0
35.0
29.9
25.9
Ph
S
SCF3
O
N
O
190 180 170 160 150 140 130 120 110 100 90 80 70 60 50 40 30 20 10
f1 (ppm)

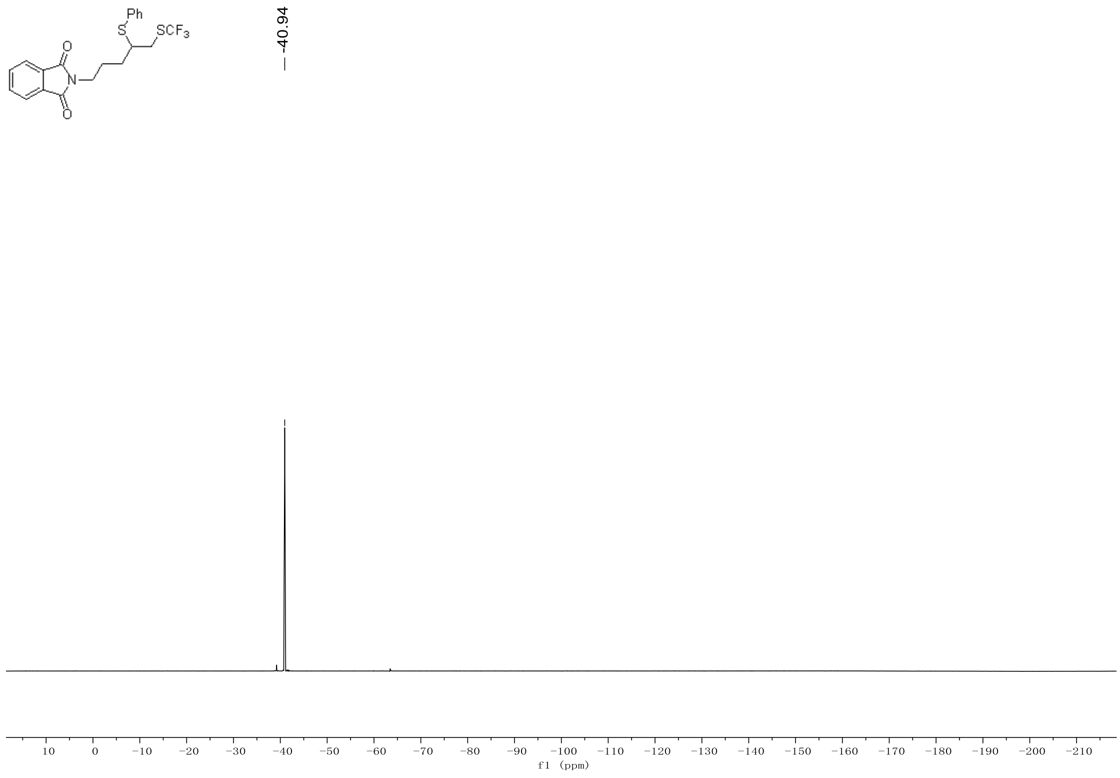
Ph
S
SCF3
O
N
O
-40.94
10 0 −10 −20 −30 −40 −50 −60 −70 −80 −90 −100 −110 −120 −130 −140 −150 −160 −170 −180 −190 −200 −210
f1 (ppm)

5-(Phenylthio)-6-((trifluoromethyl)thio)hexyl 4-methylbenzenesulfonate (5k)

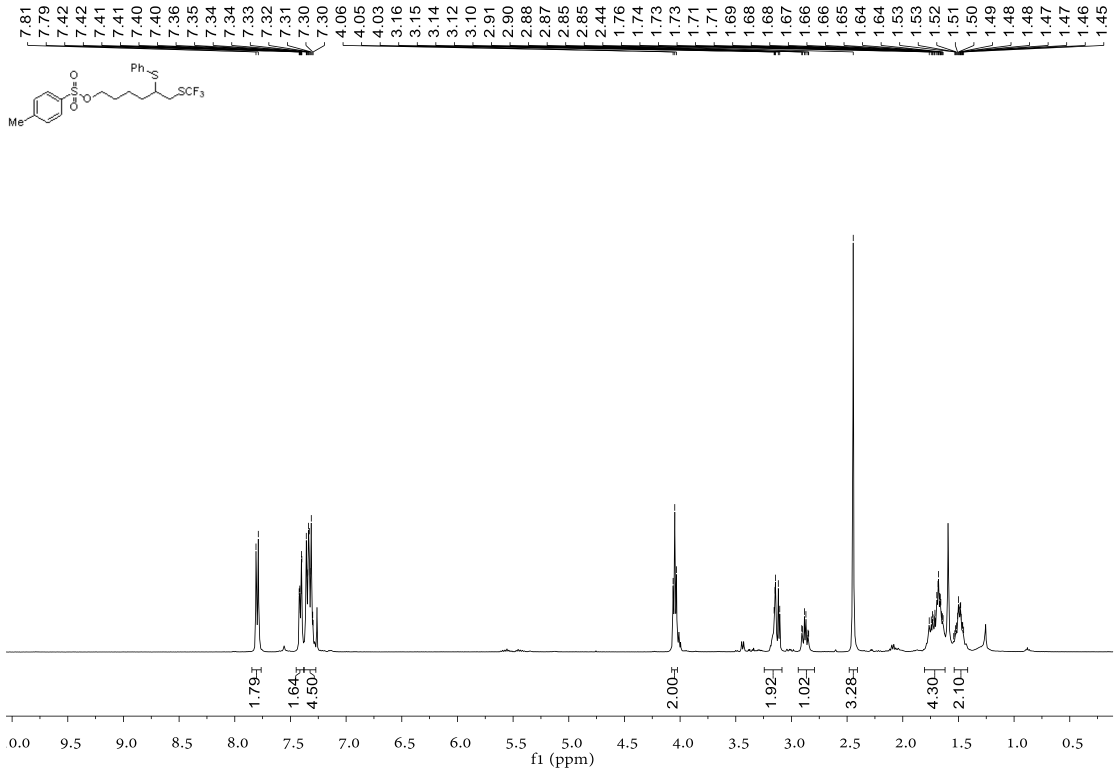

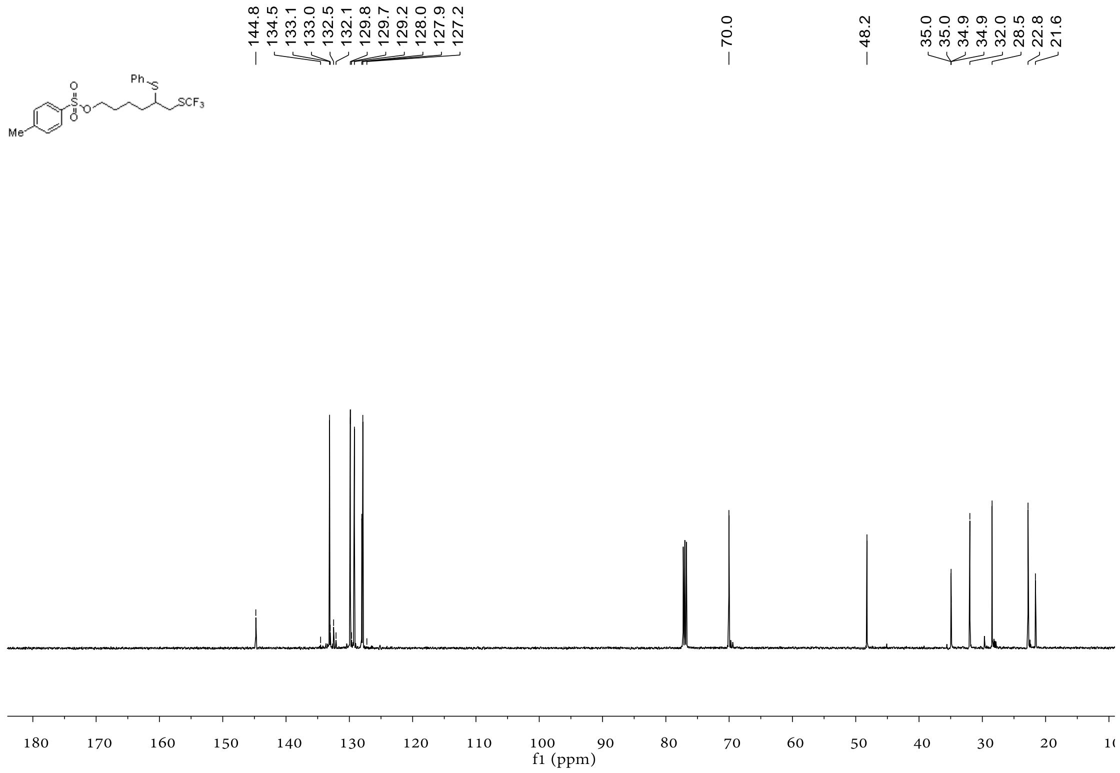

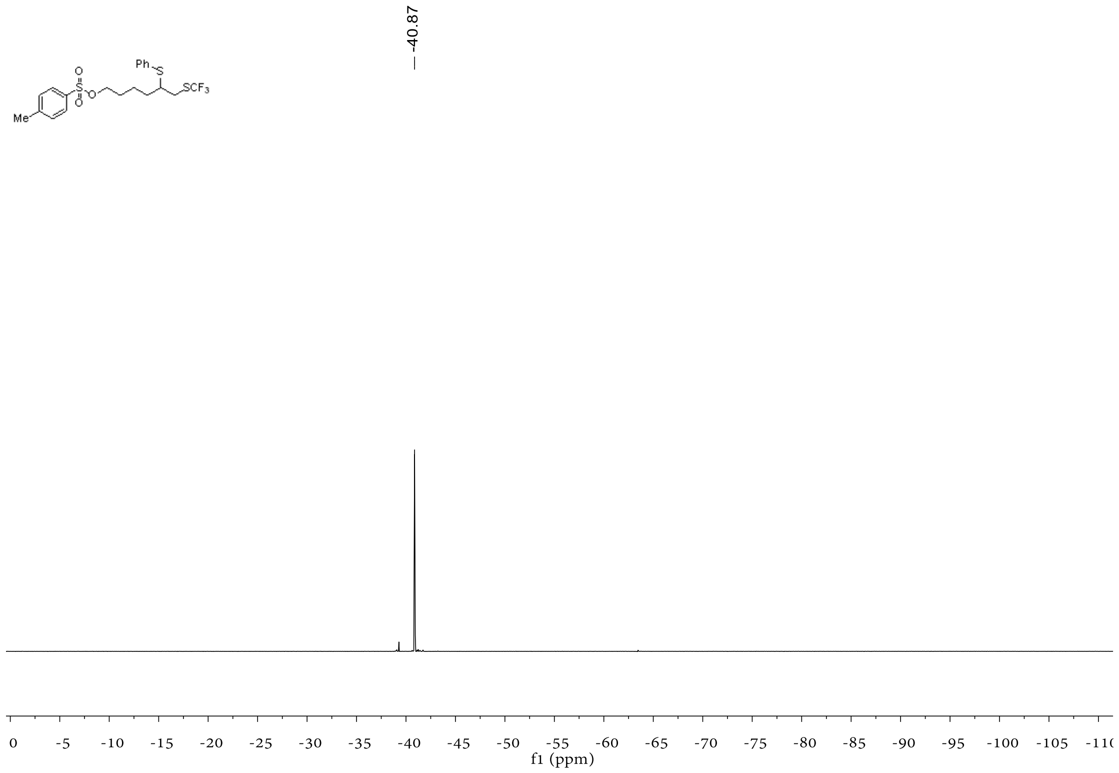

Ph
S
O
S
O
O
SCF3
Me
-40.87
0
-5
-10
-15
-20
-25
-30
-35
-40
-45
-50
-55
-60
-65
-70
-75
-80
-85
-90
-95
-100
-105
f1 (ppm)

(8*R*,9*S*,10*R*,13*S*,14*S*)-10,13-dimethyl-3-oxo-6,7,8,9,10,11,12,13,14,15,16,17-dodecahydro-3*H*-cyclopenta[*a*]phenanthren-17-yl 10-(phenylthio)-11-((trifluoromethyl)thio)undecanoate (5l)

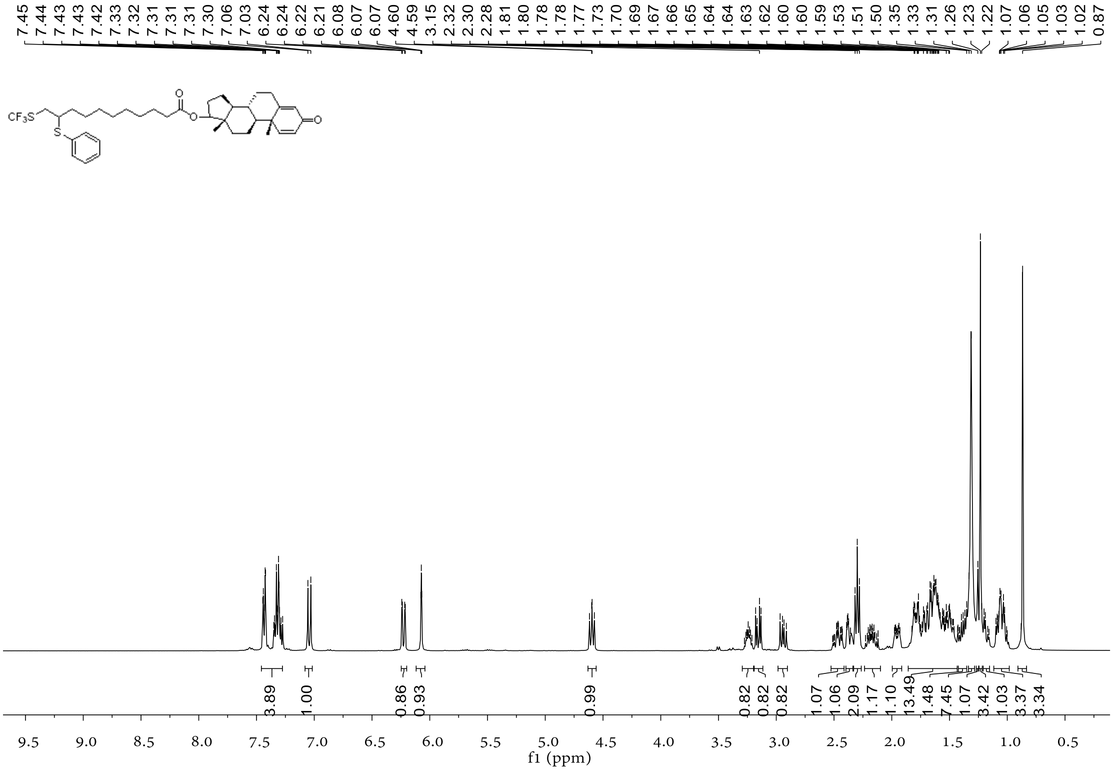

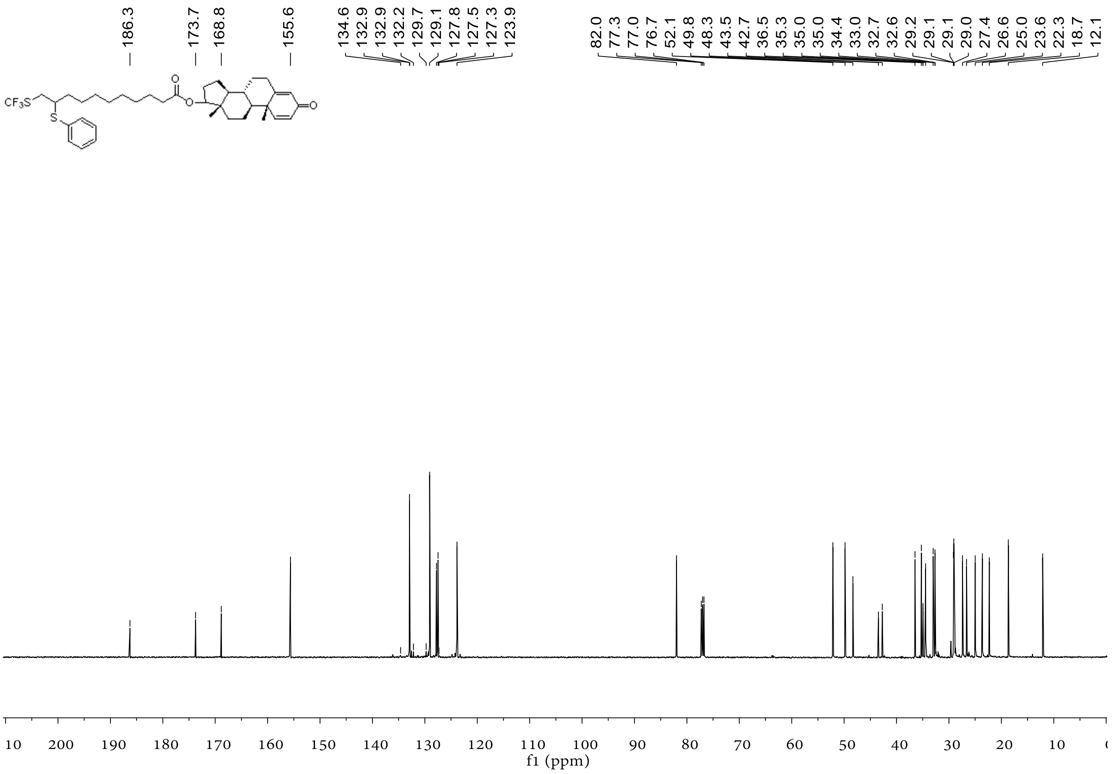

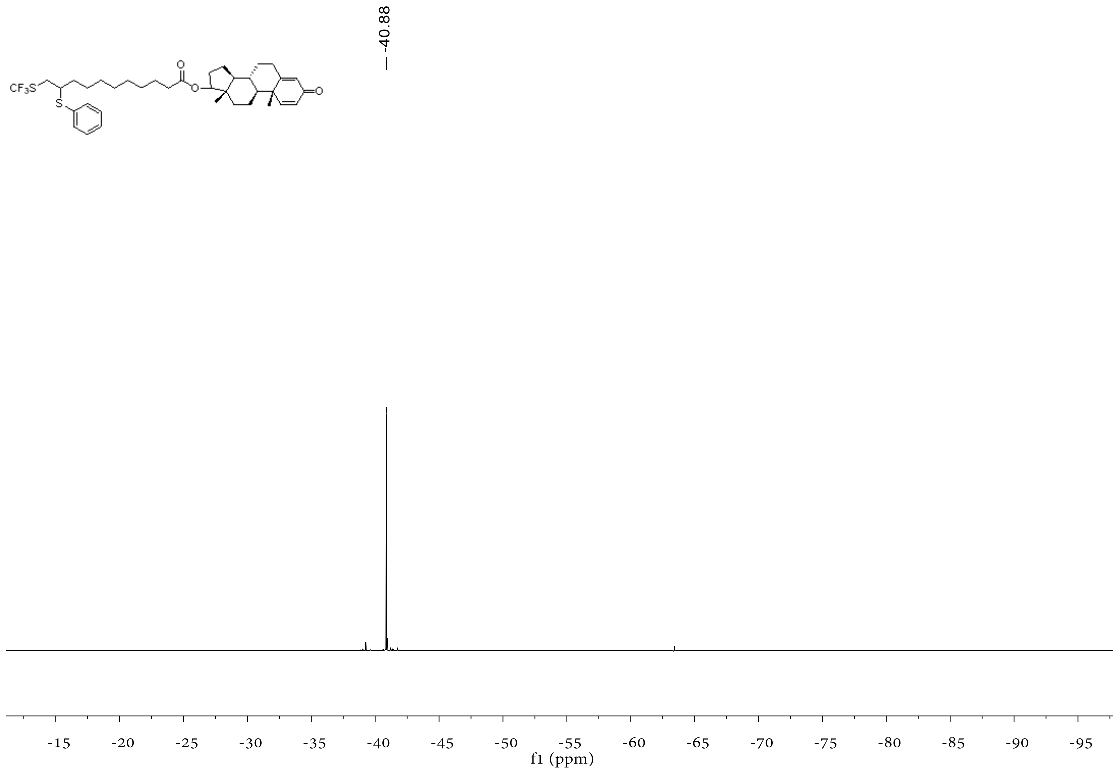

-40.88
CF3S
S
O
O
O
-15
-20
-25
-30
-35
-40
-45
-50
-55
-60
-65
-70
-75
-80
-85
-90
-95
f1 (ppm)

(3a*S*,5*S*,6*R*,6a*S*)-5-((*S*)-2,2-Dimethyl-1,3-dioxolan-4-yl)-2,2-dimethyltetrahydrofuro[2,3-*d*][1,3]dioxol-6-yl 4-(phenylthio)-5-((trifluoromethyl)thio)pentanoate (5m)

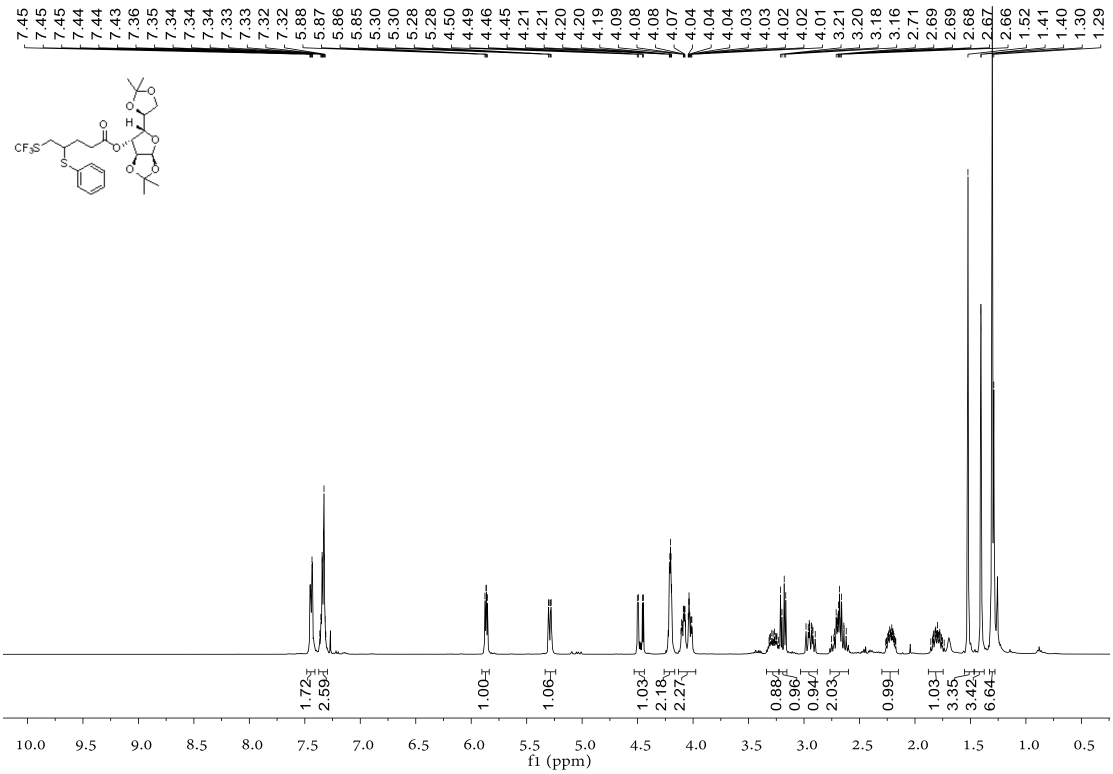

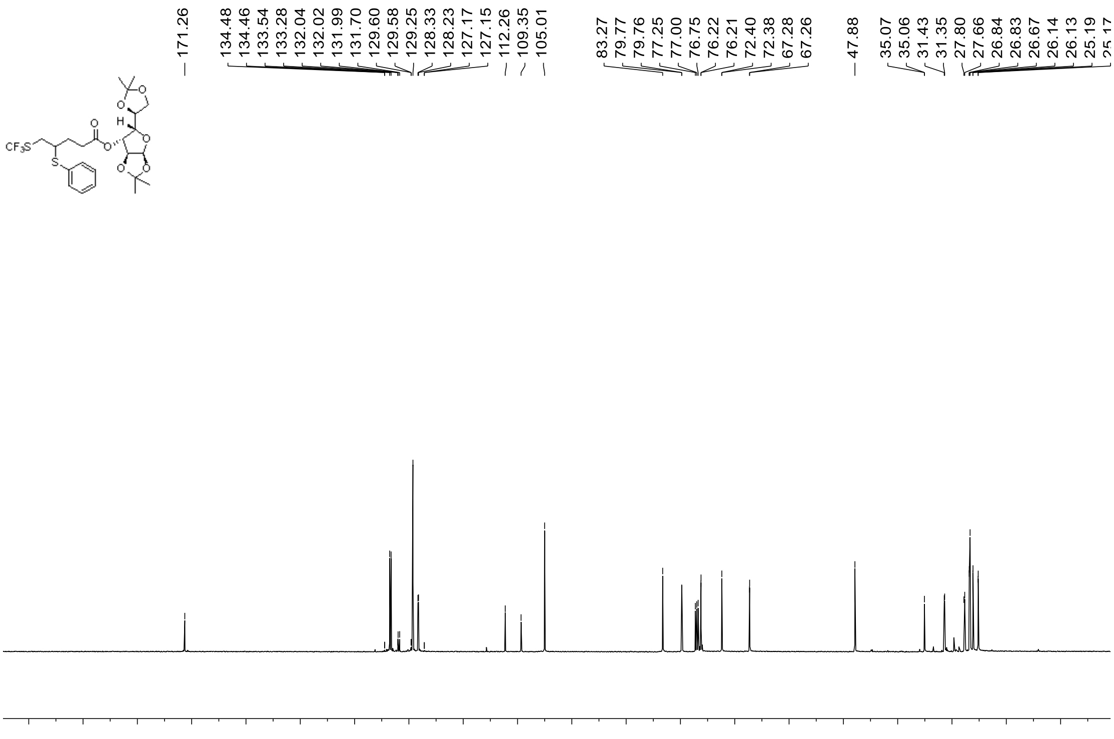

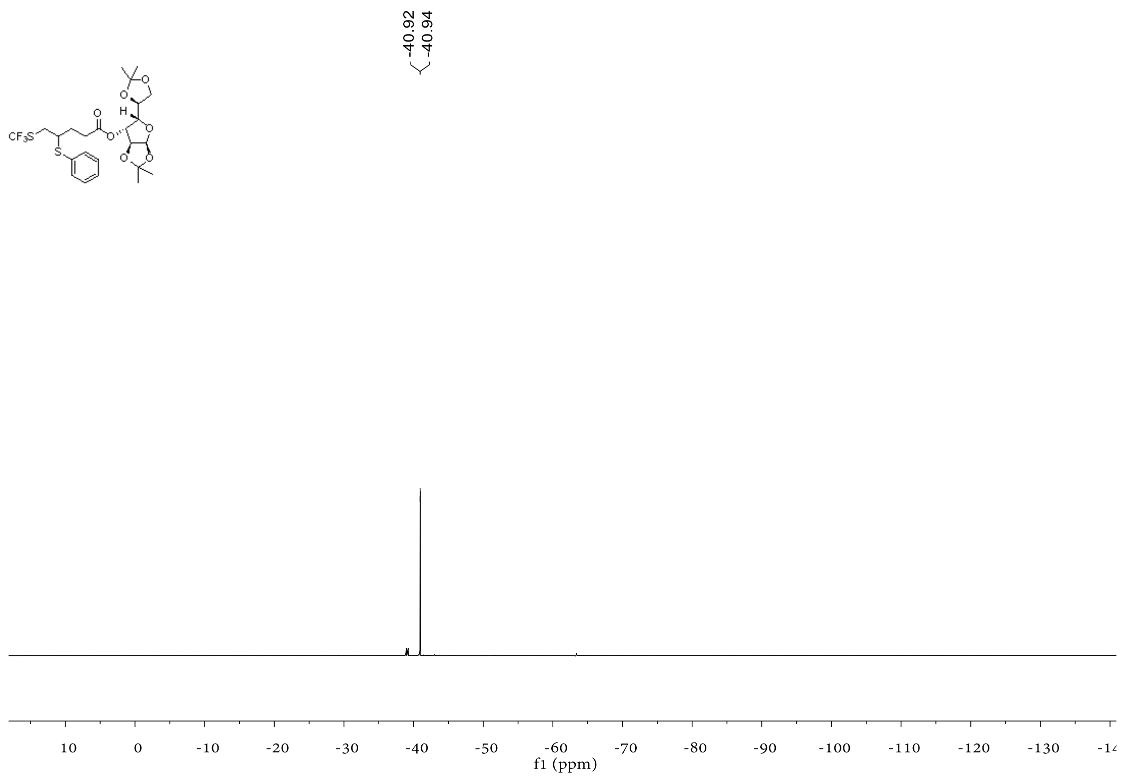
-40.92
-40.94
CF3S
H
O
S
f1 (ppm)
10
0
-10
-20
-30
-40
-50
-60
-70
-80
-90
-100
-110
-120
-130

References

[1] J. Xu, Y. Fu, D.-F. Luo, Y.-Y. Jiang, B. Xiao, Z.-J. Liu, T.-J. Gong, L. Liu, *J. Am. Chem. Soc.* **2011**, *133*, 15300–15303.
[2] B. H. Lipshutz, S. Ghorai, W. W. Y. Leong, *J. Org. Chem.* **2009**, *74*, 2854–2857.
[3] T. J. Barker, D. L. Boger, *J. Am. Chem. Soc.* **2012**, *134*, 13588–13591.
[4] Y. Xie, P. Sun, Y. Li, S. Wang, M. Ye, Z. Li, *Angew. Chem. Int. Ed.* **2019**, *58*, 7097–7101.
[5] X. Wu, L. Chu, F.-L. Qing, *Angew. Chem. Int. Ed.* **2013**, *52*, 2198–2202.
[6] S. Munavalli, D. K. Rohrbaugh, D. I. Rossman, F. J. Berg, G. W. Wagner, H. D. Durst, *Synth. Commun.* **2000**, *30*, 2847–2854.
